# Time-restricted feeding modulates the DNA methylation landscape, attenuates hallmark neuropathology and cognitive impairment in a mouse model of vascular dementia

**DOI:** 10.7150/thno.71815

**Published:** 2022-03-21

**Authors:** Sharmelee Selvaraji, Motakis Efthymios, Roger Sik Yin Foo, David Y. Fann, Mitchell Kim Peng Lai, Christopher Li Hsian Chen, Kah Leong Lim, Thiruma V. Arumugam

**Affiliations:** 1 Memory Aging and Cognition Centre, Department of Pharmacology, Yong Loo Lin School of Medicine, National University of Singapore, Singapore.; 2 Integrative Sciences and Engineering Programme, NUS Graduate School, National University of Singapore.; 3 Cardiovascular Research Institute, National University Health System, Singapore.; 4 Cardiovascular Translational Research Programme, National University of Singapore, Singapore.; 5 Genome Institute of Singapore, Agency for Science, Technology and Research, Singapore.; 6 Department of Biochemistry, Yong Loo Lin School of Medicine, National University of Singapore, Singapore.; 7 Healthy Longevity Translational Research Program, Yong Loo Lin School of Medicine, National University of Singapore, Singapore.; 8 Centre for Healthy Longevity, National University Health System (NUHS), Singapore.; 9 Department of Psychological Medicine, Yong Loo Lin School of Medicine, National University of Singapore, Singapore.; 10 Lee Kong Chian School of Medicine, Nanyang Technological University, Singapore.; 11 School of Pharmacy, Sungkyunkwan University, Suwon, Republic of Korea.; 12 Centre for Cardiovascular Biology and Disease Research, Department of Microbiology, Anatomy, Physiology and Pharmacology, School of Agriculture, Biomedicine and Environment, La Trobe University, La Trobe University, Bundoora, VIC, Australia.

**Keywords:** Intermittent Fasting, DNA Methylation, White Matter Lesion, Chronic Cerebral Hypoperfusion, Vascular Cognitive Impairment, Vascular Dementia

## Abstract

**Objective:** Vascular dementia (VaD) is the second most common cause of dementia worldwide. The increasing contribution of lifestyle-associated risk factors to VaD has pointed towards gene-environment interactions (i.e. epigenetics). This study thus aims to investigate the DNA methylation landscape in a chronic cerebral hypoperfusion (CCH) mouse model of VaD. As a nexus between the gene-environment interaction, intermittent fasting (IF) was introduced as a prophylactic intervention.

**Methods:** Bilateral common carotid artery stenosis (BCAS) was used to induce CCH by placing micro-coils of 0.18 mm in each common carotid artery of the mice. The coils were left in the mice for 7, 15 and 30 days to study temporal differences. IF was introduced for 16 h daily for 4 months prior to BCAS. Reduced Representation Bisulfite Sequencing (RRBS) was used to study the DNA methylation landscape. Cognitive impairment was measured using Barnes Maze Test. White matter lesions (WML) and neuronal loss were measured using Luxol fast blue staining and cresyl violet staining respectively.

**Results:** IF mice subjected to CCH displayed significantly better cognitive learning ability and memory, improved neuropathological alterations with reduced WMLs and neuronal loss. Modulation of DNA methylation patterns in the cortex of AL CCH mice was re-modelled and signs of reversal was observed in IF CCH mice across all three timepoints.

**Conclusions:** These findings provide an understanding of how IF may protect the brain against damage caused by CCH and show promise in offering potential beneficial effects in mitigating the neuropathology and cognitive deficits in VaD.

## Introduction

Vascular dementia (VaD) is a progressive neurodegenerative disease that results in cognitive impairment and memory loss, and is the second leading cause of dementia worldwide 1. It has a rapid, stepwise decline in disease progression and hence an associated high mortality rate 2. With age being a major risk factor, coupled with a rapidly aging population, VaD is of increasing concern warranting the need to address the issue urgently 1. Some of the clinical characteristic features of VaD include mainly reduced cerebral blood flow (CBF), increase in white matter hyperintensities and impaired blood brain barrier integrity 1. While the hallmarks of VaD pathology are well described, the underlying pathological mechanisms have yet to be fully elucidated. This has resulted in the treatments for VaD to be purely symptomatic or focused on stroke prevention. Nevertheless, the risk factors for VaD which are rather well described, aid in the diagnosis of patients due to the role played in disease onset and its progression. These risk factors include age, diabetes, hypertension, hypercholesterolemia, smoking, alcohol consumption and stress 3. With most risk factors being attributed to lifestyle-associated factors, there are also several lines of evidence which show that the environment plays a key role in driving the pathogenesis of VaD. Post-mortem and case studies of dementia patients suggest that gene-environment interactions may underlie dementia 4, but its regulatory role in VaD remains uncharacterised.

These gene-environment interactions, otherwise known as epigenetic changes, involves alterations of gene expression without any changes made to the deoxyribonucleic acid (DNA) sequence itself 5. There are three main epigenetic mechanisms, namely DNA methylation, histone modifications and non-coding RNA 6. Of these three, DNA methylation is the most widely studied which plays a key role in regulating cellular processes 5 and is the most upstream in the epigenetic hierarchy as well. It involves the covalent transfer of a methyl group to the C5 position of the cytosine ring of DNA 7. DNA methylation is generally associated to transcriptional repression of gene expression. In terms of the existing evidence exploring the role of the DNA methylation landscape in animal models of VaD, there are only two existing studies 5, 8. However, in both these studies, the animal model used is an occlusion model whereby there is a complete blockage of blood vessels and hence blood supply, thus incurring acute damage. This does not represent the CCH state observed in VaD patients and also results in damage to the visual pathway, which does not occur in patients. Hence, there is an impending need to study the DNA methylation landscape in a suitable model of VaD. The Bilateral Common Carotid Artery Stenosis (BCAS) is a CCH model which is known to be the most promising model of VaD 1. BCAS very closely mimics the conditions in VaD patients with increased white matter lesions, impaired blood brain barrier integrity, increased inflammatory response and reduced brain metabolism alongside a chronic reduction in CBF.

Given the implications of the gene-environment interaction in VaD and the contribution of lifestyle-associated risk factors, changes to diet and lifestyle could prove to be useful. With the metabolic syndrome playing a key role in driving disease pathogenesis, metabolic regulation in the form of altered dietary patterns could perhaps help to slow down if not prevent disease progression. Of the different dietary regimens, intermittent fasting (IF) has been shown to be protective against age-related neurological diseases 9. While, a few studies which show that IF physiologically modulates DNA methylation 10-12, further validation and extensive studies are required to assess its benefits especially in a pathological state such as that in VaD. Essentially, the presence of, if any, DNA methylation landscape changes and their regulations under CCH remain uncharacterised. The potential of IF as a prophylactic therapeutic intervention also remains untapped. Therefore, to address these gaps, this study for the first time, 1) investigates the effect of IF on the DNA methylation landscape under physiological conditions, 2) investigates how the DNA methylation landscape changes in a CCH mouse model of VaD, and 3) explores the role of prophylactic IF as a modifier of the DNA methylation landscape in a pathological state. Here we show that the DNA methylation landscape is altered in a CCH mouse model of VaD. Mapping of the DNA methylation patterns under CCH in the AL-fed and IF mice provided insights into the landscape present under the diseased state compared to when a prophylactic intervention is introduced. Furthermore, IF mice subjected to CCH displayed significantly better learning ability and reduced latency and significant protection against CCH-induced neuropathological alterations.

## Methods

### Experimental Animals and Intermittent Fasting

All *in vivo* experimental procedures were approved by the National University of Singapore, Singapore Animal Care and Use Committee and performed according to the guidelines set by the National Advisory Committee for Laboratory Animal Research (NACLAR), Singapore. All experiments in the manuscript were performed and reported according to Animal Research: Reporting *In vivo* Experiments (ARRIVE) guidelines. Eight weeks old male C57BL/6 mice were obtained from *In vivo*s, Singapore. Mice were housed in individual cages under standard laboratory conditions. Efforts were taken to minimise the number of animals used and to minimise their suffering during procedures. All mice had free access to food and water ad libitum (AL) for the first week during the acclimatisation period. The experimental groups consisted of 15-16 mice in each group. Mice were randomly assigned to either AL or intermittent fasting (IF) dietary conditions. Mice under IF conditions had their food pellets removed for 16 h every day with food available from 0700 to 1500 for four months before any form of surgical interventions were performed. Water was available for 24 h under both AL and IF conditions. There were a total of 14 different experimental groups: AL wildtype, IF wildtype, AL BCAS 7 days, AL BCAS 15 days, AL BCAS 30 days, AL Sham 7 days, AL Sham 15 days, AL Sham 30 days, IF BCAS 7 days, IF BCAS 15 days, IF BCAS 30 days, IF Sham 7 days, IF Sham 15 days and IF Sham 30 days. The three different timepoints indicate the duration the mice were subjected to BCAS surgery as detailed in the subsequent sections.

### Blood Glucose and Ketone Measurements

Approximately 1 to 2 mm of tissue was cut from the mouse tail and the blood obtained from direct flow was collected on blood glucose (Optium, Abbott, UK) and blood β-ketone (Optium Ketone, Abbott, UK) test strips, respectively. The measurements were subsequently made using an Optium Xceed diabetes monitoring system (Abbott, UK). The blood glucose and ketone levels were measured once every two weeks for 18 weeks.

### Bilateral Common Carotid Artery Stenosis (BCAS) Mouse Model

Animals were anaesthetized with isoflurane and subjected to BCAS. BCAS involved using microcoils that were specially designed for the mice (Piano wire diameter 0.08 mm, internal diameter 0.18 mm, coiling pitch 0.5 mm, and total length 2.5 mm; Sawane Spring Co Ltd, Japan). BCAS was performed by exposing the left and right common carotid arteries (CCAs) one-by-one, freed from their sheaths, and twining a microcoil by rotating it around each CCA. Sham surgeries were performed as controls where the site of surgery was opened and the CCAs were gently touched using the forceps without insertion of microcoils. The site of surgery was subsequently closed using surgical glue, and the mice were observed and taken care of post-surgery until conscious and recovered to freely access food and water ad libitum. All animals were euthanized via inhalation of carbon dioxide gas at their respective time points (7, 15 and 30 days respectively) after BCAS for subsequent analysis.

### Cerebral Blood Flow (CBF) Measurements

High-resolution Laser Speckle Contrast Imager (PSI system, Perimed Inc.) was used to image cerebral blood perfusion and record cerebral blood flow (CBF) before insertion of the microcoils (baseline), immediately after the insertion of the microcoils and finally at their respective endpoints of BCAS. Body temperature of the mice was maintained at 37 ± 0.5°C. The skull of the mice were shaved and exposed by a midline skin incision. The skull was cleaned gently with 1x sterile phosphate buffered saline (PBS) using a cotton applicator. The image area was kept moist and a non-toxic silicon oil was applied on the skull, improving the imaging. Perfusion images were acquired using the PSI system with a 70 mW built-in laser diode for illumination and a 1388 x 1038 pixels CCD camera installed 10 cm above the skull (speed 19 Hz, and exposure time 6 ms). Analyses of CBF changes were done on the acquired images.

### Behavioural Assessments

***Open Field Test:*** Locomotor activity was measured using the open field test. Each mouse was placed at the centre of the open field apparatus (25x25x46 cm); Clever Sys Inc., VA, USA). The total distance travelled (in mm) and average velocity (in mm/s) were tracked and recorded via TopScan (Clever Sys Inc.; VA, USA). Data was collected for a period of 30 min.

***Barnes Maze Test:*** Barnes maze test was performed in 8-months-old C57BL/6 male mice (3 weeks post-BCAS operation). The test was performed on an elevated, circular platform which is 90 cm in diameter with 16 holes equidistant around the perimeter 13. Under one of the holes, an escape box is placed. Spatial cues were placed around the maze and were kept constant throughout the study period. During the *Habituation phase*: The mice were acclimatised to the room for at least 1 h prior to start of testing. During the *Acquisition phase*: On the first day of the test, the mouse was placed in the escape box for 1 min before being placed at the centre of the maze inside a black chamber. The chamber was removed after 10 s, upon which light (400 lux) was turned on and the mouse was allowed to freely explore the maze for 3 min or until the mouse entered the escape tunnel. Light was turned off once the mouse entered the tunnel and the hole was covered for 15 s. This test was subsequently repeated once daily for 4 days. The latency to enter the target was scored. The platform was moved by 90^o^ every day to prevent any odour cue but the spatial cues and tunnel position was kept constant. During the *Probe trial* (long term retention phase): On the 5^th^ day, the probe test was conducted where the target hole was blocked. The escape tunnel was removed and each mouse was allowed to explore the maze for 3 min to assess their spatial memory. The time spent in the target quadrant and time taken to sniff the target were scored. During the *Remote probe test* (spatial memory retrieval): On the 15^th^ day, the mice were retested, similar to the probe trial and time spent in target quadrant and frequency of visits to target were recorded. All measurements were tracked and recorded via TopScan (Clever Sys Inc.; VA, USA).

### Luxol Fast Blue (LFB) and Cresyl Violet Staining

Mouse brain sections were fixed in 4% paraformaldehyde in 1x phosphate-buffered saline (PBS) and then embedded into paraffin wax blocks. 4.5 µm thick coronal sections were obtained through microtome sectioning. To visualise and quantify the severity of white matter lesions, LFB was performed. Briefly, the sections were de-waxed, rehydrated and immersed in LFB solution (Abcam, UK) at 37 ^o^C overnight. Excess staining was removed using 95% ethanol and subsequently washed with deionised water. Sections were exposed to 0.05% aqueous lithium carbonate (Abcam, UK) for 20 s to initiate differentiation of grey and white matter followed by 70% ethanol till the nuclei are decolorised. Following which, the sections were immersed in Cresyl Violet solution (Abcam, UK) for 5 min, washed in deionised water and dehydrated before cleared in xylene and mounted for microscopy. The LFB staining were visualised using the Olympus upright Fluorescence Microscope BX53. The white matter lesions were evaluated in the caudoputamen and corpus callosum (Median and Paramedian) regions. The severity of the lesions were given a grade as either normal (Grade 0), disarrangement of nerve fibres (Grade 1), formation of marked vacuoles (Grade 2) or disappearance of myelinated fibres (Grade 3). Quantification of the lesions was done by averaging the grade scores provided by 5 blinded examiners.

### DNA Methylation Analysis

***Sample Collection:*** Upon euthanising the mice via administration of a lethal dose of inhaled carbon dioxide, the brains were harvested. The cerebral cortex was immediately separated and were kept frozen in the -80^o^C freezer for DNA methylation and biochemical analysis (n=7-8 in each experimental group).

***DNA Extraction and Validation:*** Total dsDNA was extracted from frozen cerebral cortex tissue samples using a micro-tube tissue homogeniser (Bel-Art, Wayne, NJ, USA) and GF-1 tissue nucleic acid extraction kit (Vivantis, Malaysia) following the manufacturer's instructions. 20 mg of cerebral cortex tissue was used to extract about 4-9 µg of DNA. To ensure that the DNA extracted is pure and of sufficient amount, quality control experiments were performed. 2 µl of DNA extracted from each sample was placed in a Nanodrop ND-1000 (Thermo Fisher Scientific, Waltham, USA) to measure and assess the purity and concentration of samples. A 260/280 ratio of absorbance of ~1.8 was considered to be pure. The DNA fragments were separated by size and the absence of RNA contamination was ensured through gel electrophoresis (1.0% agarose gel). The dsDNA concentration was quantified using QuantiFluor dsDNA system dye (Promega, USA) using the Quantus^TM^ Fluorometer (Promega, USA) as per the manufacturer's instructions.

***Reduced Representation Bisulfite Sequencing (RRBS):*** Extracted DNA samples were sent to Novogene (China) for RRBS. The DNA samples were digested using methylation-insensitive restriction enzyme Mspl. All the cytosines were subjected to methylation-modified sequencing adaptors, DNA fragments with insert lengths within the range of 40-220 base pairs were cut from the gel and bisulfite treatment was carried out using an EZ DNA Methylation Gold Kit (Zymo Research). PCR amplification was then performed to obtain the final DNA library. After quality checks of the DNA library, the samples were sent for Illumina HiSeq 2500/4000 sequencing.

***Bioinformatics Pipeline:*** Image analysis and base calling were performed with Illumina CASAVA pipeline and 125bp/150 base pairs paired-end reads were generated. FastQC (fastqc_v0.11.5) was used to check the quality of the raw reads. Trim Galore was used to trim the adaptor sequences and to automate quality. Bowtie2 was used to map the sequences to a reference genome and for alignment. Bismark software (version 0.16.3) was also used for alignment of bisulfite-treated reads to a reference genome and to filter the reads after alignment. The methylation score for each CpG site is represented as a β-value which ranges between 0 and 1 where the former indicates no methylation and the latter 100% methylation at the specific CpG site measured. These β-values were used to calculate relative differences between samples and experimental groups to reflect the methylation status. Higher values corresponded to greater level of methylation (hypermethylation) and lower values corresponded to a lower level of methylation (hypomethylation) in relative terms. The significance of a differentially methylated region (DMR) is weighted by AreaStat which is the sum of t-statistic values of all CpG sites within each DMR. The significant DMRs were identified through BiSeq package and DSS software. The DMRs were then sorted by C-context (i.e. CG, CHG or CHH), promoter region and the associated genes were annotated using the R-package: methylKit (version 1.20.0).

***Heatmap Generation and Enrichment Analysis:*** Heatmaps of differentially methylated genes were created using the R packages gplots and heatmap2. Gene Ontology (GO) enrichment analysis of genes was performed via GOseq R Package with gene length bias corrected for. GO terms provide information about the biological domain of a set of genes based on molecular function, cellular component and biological function. The KEGG database was used to identify potential pathways the genes would be involved in.

***STRING Database Analysis:*** The potential protein-protein interactions (PPI) among the hyper- and hypo-methylated genes were identified using the STRING database 14. STRING has a rich coverage of 24,584,628 proteins from 5090 organisms where it performs a computational prediction of PPI based on previous knowledge in primary databases, automated text mining, computational interaction predictions of co-expression, high-throughput lab experiments and genomic context predictions.

### Immunoblot Analysis

Cerebral cortex tissues were homogenised in lysis buffer (Thermo Fisher Scientific, #78510), and combined with protease inhibitors (Thermo Fisher Scientific, #78443) and phosphatase inhibitors (Thermo Fisher Scientific, #78428) to prevent proteolysis and dephosphorylation of proteins respectively during extraction. 2x Laemelli buffer (Bio-Rad Laboratories, Inc., Hercules, CA, USA) was then added to it. The protein samples were subsequently subjected to sodium dodecyl sulfate-polyacrylamide (5 to 12.5%) gel electrophoresis using Tris-glycine running buffer. The gels were then electro-blotted using a transfer apparatus (Bio-Rad Laboratories, Inc., Hercules, CA, USA) in transfer buffer containing 0.025 mol/L Tris base, 0.15 mol/L glycine and 10% (v/v) methanol for approximately 1 h 40 min at 350 mA onto nitrocellulose membrane. Next, the nitrocellulose membranes were incubated with the following primary antibodies: MRP4/ABCC4 (Cell Signalling Technology, #12705), GNAS (Abcam, ab83735), ACTN1 (Abcam, ab18061), DNMT1 (Cell Signalling Technology, USA, #5032), DNMT3A (Cell Signalling Technology, USA, #2160), DNMT3B (Cell Signalling Technology, USA, #44145), TET1 (Abcam, ab191698), GFAP (Cell Signalling Technology, USA, #12389S), IBA1 (Abcam, ab5076), MBP (Cell Signalling Technology, USA, #78896S), Vinculin (Cell Signalling Technology, USA, #13901S) and β-actin (Sigma-Aldrich, A5441) overnight at 4 ^o^C with agitation. Following primary antibody incubation, membranes were washed three times with 1xTBST, each time for 10 min, before incubating with horseradish peroxidase (HRP)-conjugated secondary antibodies (Goat Anti-Rabbit - Cell Signaling Technology, Danvers, MA, USA; Goat Anti-Mouse- Sigma Aldrich, St Louis, MO, USA) for 1 h at 24 ^o^C with agitation. After the secondary antibody incubation, membranes were washed three times with 1xTBST, each time for 10min. Enhanced chemiluminescence (Bio-Rad Laboratories, Inc., Hercu.es, CA, USA), the substrate for HRP, was applied before the membranes were imaged using the ChemiDocXRS+ imaging system (Bio-Rad Laboratories, Inc., Hercules, CA, USA). Quantification of proteins was done using the Image J software (Version 1.46; National Institute of Health, Bethesda, MD, USA), where protein densitometry was expressed relative to the densitometry of the corresponding Vinculin or β-actin levels.

### Statistical Analysis

All experimental data were analysed using GraphPad Prism 8.01 (GraphPad Software, San Diego, CA, USA). All values are expressed as mean ± standard error of mean. Statistical significance was calculated using Student's t-test (p<0.05). The Mann Whitney Test was used for behavioural data analysis. A p-value <0.05 (95% confidence interval) was considered to be statistically significant.

## Results

### Intermittent fasting (IF) induces a metabolic switch and improves working memory and modulates the DNA methylation landscape under physiological conditions

To validate the effectiveness of implementing IF, the body weight, blood glucose and ketone levels of the mice were regularly measured. The body weight of IF mice were significantly lower than the ad libitum (AL) fed mice (Figure S1A). This was consistently observed during the intervention period of 6 months. The possibility of caloric restriction (CR) as a confounder for the observed weight loss was minimised by monitoring the food consumption of the AL fed and IF mice where the latter showed a 9.6% reduction (Figure S1A), less than the range of 20-40% reduction which defines CR 15. The blood glucose and ketone levels were also monitored in the mice to determine the effectiveness of the IF regimen. Evidently, the blood glucose levels were observed to be generally lower in the IF mice then the AL-fed mice (Figure S1B). The ketone levels were significantly higher in the IF mice as opposed to the AL-fed mice (Figure S1B), thus indicating a metabolic switch.

**Figure 1:**
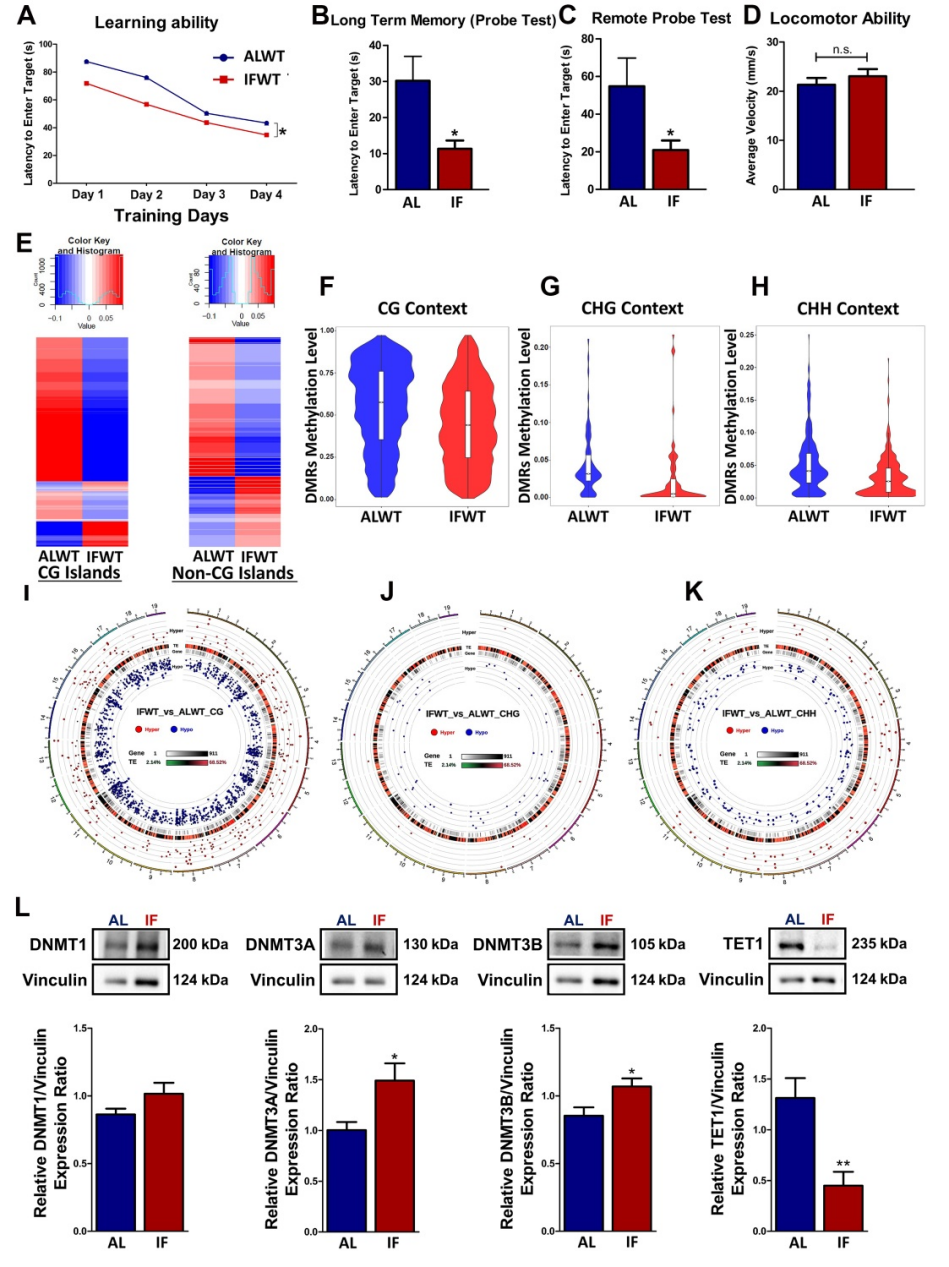
** Intermittent fasting (IF) improves cognitive function and modulates the DNA methylation landscape under physiological conditions.** Quantification of Barnes maze test conducted on AL and IF mice under wildtype (WT) conditions at the 30-day timepoint (n=8-10 mice per experimental group). (A) The learning ability of IFWT mice was measured using the latency to enter the target, which were significantly lower than the ALWT mice in the acquisition phase of 4 days. (B) The long-term memory and (C) remote probe test (spatial memory retrieval) measured on the 5^th^ and 15^th^ day respectively showed a significant reduction in the latency to enter the target in the IFWT compared to the ALWT mice indicating a better long-term retention phase and spatial memory retrieval respectively. (D) The mice were subjected to an open field test to ensure the absence of locomotor disabilities to eliminate its confounding effect on the reference memory probe test. No significant difference was observed across the experimental groups in terms of average velocity of the mice during the open field test. An Unpaired t-test with Bonferroni correction was conducted, *p<0.05 compared to ALWT. (E) Heatmap of differentially methylated genes in the ALWT compared to the IFWT mice under CG-dinuleotide rich islands (CGI) and under non-CGI such as CHG and CHH contexts. (F-H) Violin plots illustrating a general decline in methylation levels in IFWT compared to ALWT mice in all three contexts of CG, CHG and CHH. (I-K) Circos representation graphs building on the violin plot data to show differentially methylated genes being more hypomethylated in the IFWT mice compared to the ALWT mice in all three CG, CHG and CHH contexts as represented by the blue dots in the internal rim of the graphs. (L) Representative immunoblots and quantification illustrating significantly higher levels of DNA methyltransferases (DNMT1, DNMT3A, DNMT3B) protein abundance in the IF WT mice compared to the AL CCH mice at the 30-day timepoint. The protein levels of TET1 was significantly lower in the IFWT compared to ALWT mice. Vinculin was used as a loading control. Data are represented as mean ± SD. n=5-7 mice in each experimental group. Unpaired t-test, Bonferroni correction, *p<0.05 when compared with ALWT.

To investigate whether IF improves cognitive function and modulates the DNA methylation landscape under normal physiological condition, behavioural assessment and reduced representation bisulfite sequencing (RRBS) were performed and compared between AL wildtype (ALWT) and IFWT mice respectively. IFWT mice showed significantly improved learning ability (Figure 1A), lower latency to enter the target quadrant during the probe test (Figure 1B) and remote probe test (Figure 1C). These changes in latency were not due to any locomotor disabilities as confirmed by the open field test (Figure 1D).

The global DNA methylation levels in ALWT and IFWT mice were visualised using heatmaps, violin plots and circos representation graphs. While DNA methylation is found in three different contexts: CG, CHG and CHH, the role of CHG and CHH remain unknown. Therefore, the effect of IF on the DNA methylation landscape was analysed in all three different contexts for preliminary investigations. The global DNA methylation pattern in the IFWT mice exhibited distinct deviation from the ALWT mice as observed in the heatmap plotted for differentially methylated genes (DMGs) both in CG nucleotide-rich islands (CGIs) and non-CGIs (Figure 1E). This deviation in methylation status in IFWT mice was towards the hypomethylated state. The differentially methylated regions (DMRs) in the IFWT mice had a generally lower global methylation level compared to the ALWT mice as represented using violin plots for all three contexts (Figure 1F-H). Moreover, the circos representation graphs (Figure 1I-K), which provide a visual representation of the hypo- and hyper-methylated genes sorted by respective chromosomes, gives a quantitation of the violin plots by illustrating the increased number of hypomethylated genes marked by blue dots in the IFWT compared to ALWT mice. The breakdown of the number of hypo- and hyper-methylated genes in all gene regions (Figure S2A) and those in the promoter region (Figure S2A) are analysed. In addition, how these respective hypo- and hyper-methylated genes are linked is investigated by clustering them based on their potential protein-protein interaction networks (Figure S2B-C). The Gene Ontology (GO) terms and KEGG pathway analyses were also performed to better understand and interpret the potential biological role of the hypo- and hyper-methylated genes analysed (Figure S3A-F). To investigate the role of DNA methyltransferases (DNMT) and demethylases in modulating methylation of genes, the abundance of DNMT1, DNMT3A, DNMT3B and Ten-eleven translocation 1 (TET1) were studied (Figure 1L). DNMT3A and DNMT3B showed a statistically significant increase in abundance while TET1 showed a statistically significant decrease in IF WT mice compared to ALWT mice. DNMT1, however showed similar trends but without statistical significance. This finding shows that DNMTs and TET1 play a role in modulating the expression of DMGs in a physiological state.

### Intermittent fasting (IF) ameliorates cognitive impairment and hallmark neuropathology incurred by chronic cerebral hypoperfusion

Firstly, to validate the effectiveness of BCAS surgery in inducing a chronic cerebral hypoperfused (CCH) state, proof-of-concept experiments were conducted in the presence and absence of IF. The post-BCAS reduction in cerebral blood perfusion of approximately 25% was both visually and statistically evident after BCAS induced CCH in both AL-fed and IF mice across all the three different timepoints (Figure S4A-B). On the other hand, the sham mice showed no difference in cerebral blood perfusion as expected as there was no stenosis of the common carotid arteries.

**Figure 2:**
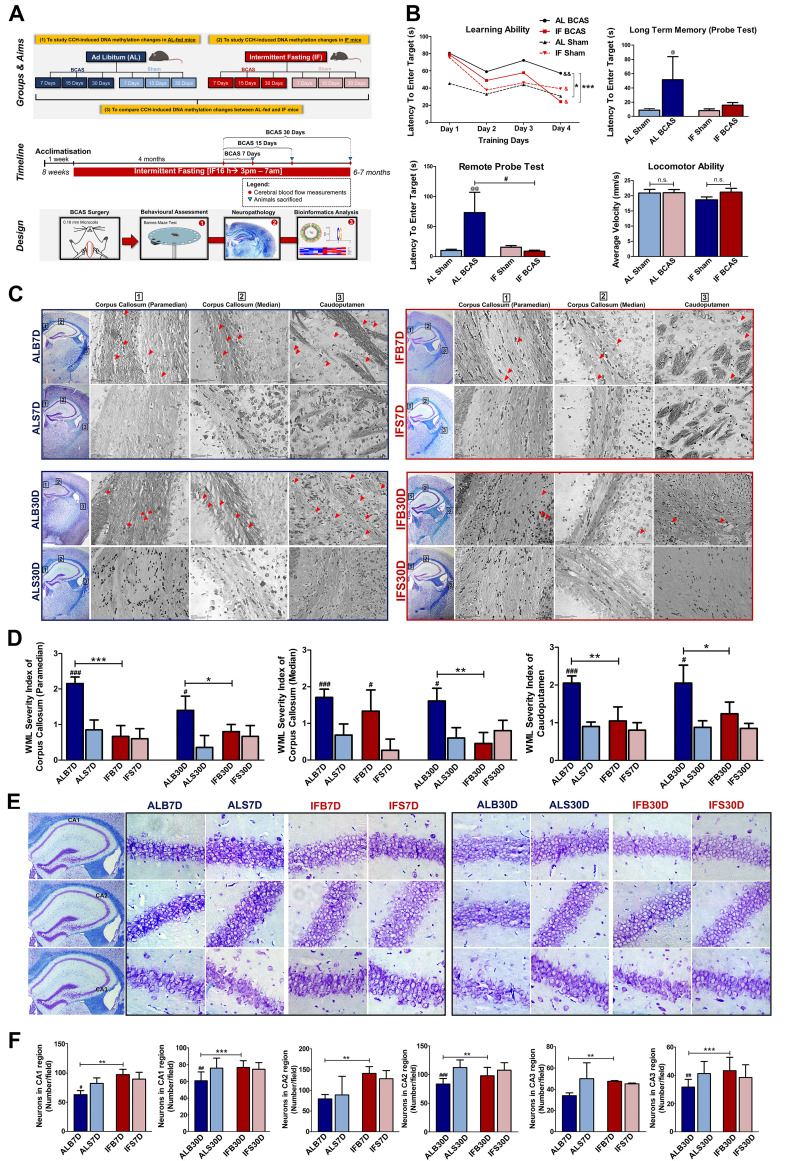
** Intermittent fasting (IF) attenuates cognitive impairment and neuropathological alterations under chronic cerebral hypoperfusion (CCH).** (A) Schematic outline of experimental groups and aims, timeline and design. The mice were divided into two main groups of ad libitum (AL)-fed and IF mice and further divided into mice that underwent Bilateral Common Carotid Artery Stenosis (BCAS) surgery and sham surgery respectively. The three main study aims are to (1) study CCH-induced DNA methylation changes in AL-fed mice (AL CCH vs AL Sham mice), (2) study CCH-induced DNA methylation changes in IF mice (IF CCH vs IF Sham mice) and (3) compare CCH-induced DNA methylation changes between AL-fed and IF mice (AL CCH vs IF CCH mice). The IF mice were subjected to 16 h of fasting every day for 4 months before undergoing BCAS surgery for 7, 15 and 30 days accordingly. Similarly, the AL-fed mice underwent BCAS surgery for 7, 15 and 30 days at 6 months of age. Both the AL and IF BCAS mice had timepoint-specific sham groups. The mice were subjected to Sham or BCAS surgery at 6 months of age followed by a Barnes maze test, neuropathological assessments, and DNA methylation sequencing and analysis. (B) Quantification of Barnes maze test conducted on AL and IF mice under CCH at the 30-day timepoint (n=8-10 mice per experimental group). The learning ability of IF mice was measured using the latency to enter the target, which were significantly lower than the AL CCH mice in the acquisition phase of 4 days. The long-term memory and remote probe test (spatial memory retrieval) were measured on the 5^th^ and 15^th^ day that showed a significant reduction in the latency to enter the target in the IF CCH mice compared to the AL CCH mice indicating a better long-term retention phase and spatial memory retrieval respectively. The mice were subjected to an open field test to ensure the absence of locomotor disabilities so as to eliminate its confounding effect on the reference memory probe test. No significant difference was observed across the experimental groups in terms of average velocity of the mice during the open field test. An Unpaired t-test with Bonferroni correction was conducted, ^&^p<0.05, ^&&^p<0.01 compared with the respective experimental group's Day 1 latency measuring learning ability, *p<0.05, ***p<0.001, ^#^p<0.05 and ^@^p<0.05, ^@@^p<0.01 compared to AL Sham. (C) Representative Luxol fast blue stained images illustrating white matter lesions (WML) in the corpus callosum paramedian (CCP), corpus callosum median (CCM) and caudoputamen (CP) regions at the 7 and 30-day timepoints of the AL-fed CCH, IF CCH and the respective sham mice. A distinct decrease in WML severity was observed in the IF compared to the AL mice when subjected to CCH. The WML severity index was subsequently quantified through a grading score: Grade 0 = no disarrangement of nerve fibres (normal), Grade 1 = disarrangement of nerve fibres, Grade 2 = formation of marked vacuoles and Grade 3 = disappearance of myelinated fibres. Red arrow heads indicate marked vacuoles. (D). Quantification of the WML severity index showed a statistically significant decrease in the IF CCH mice compared to the AL CCH mice at the 7-day timepoint in the CCP and CP regions. A consistent significant decrease in WML severity index was observed in the IF CCH mice compared to the AL CCH mice in all regions at the 30-day timepoint. (E) Representative cresyl violet images illustrated increased Nissl positively stained neurons in hippocampal CA1, CA2 and CA3 regions of IF CCH mice (IFB) compared to AL CCH mice (ALB) against their respective sham groups IFS and ALS. (F) Quantification of neuronal counts in cresyl violet images showed a statistically significant increase in neurons that were consistently observed in all three hippocampal regions of IF CCH mice compared to AL CCH mice at the 7-day timepoint. A significant increase in neuronal count was observed only in the CA2 hippocampal region of IF CCH mice compared to the AL CCH mice at the 30-day timepoint. Magnification x60. Data are represented as a mean ± SD. n= 4-7 mice in each experimental group. ^#^p<0.05, ^###^p<0.001 compared to ALS7D or IFS7D accordingly. *p<0.05, **p<0.01, ***p<0.001.

In order to investigate the effect of IF in the CCH model of VaD, cognitive function and neuropathology were assessed (Figure 2A). AL-fed mice subjected to CCH (AL BCAS) showed significantly poorer learning ability and longer latency to enter the target quadrant as compared to the respective sham controls (Figure 2B). By contrast, IF mice subjected to CCH (IF BCAS) displayed significantly better learning ability and reduced latency to reach the desired target quadrant compared to the AL BCAS mice (Figure 2B). It is notable that there was no significant difference between the latency to reach the target quadrant between the IF Sham and IF BCAS mice. These changes in latency were not due to any locomotor disabilities as confirmed by the open field test (Figure 2B). Cognitive impairment is thus observed under CCH and prophylactic IF ameliorates this impairment to levels comparable to that of the respective sham mice.

White matter lesions (WMLs) in the caudoputamen and corpus callosum regions were visualised through Luxol fast blue staining of myelinated axons 16. The WMLs observed in the AL BCAS mice were significantly higher than in the AL Sham mice across all different timepoints (Figure 2C-D). It is notable that the WMLs observed in the IF BCAS mice was significantly reduced than in the AL BCAS mice suggesting improvement in the neuropathological alterations (Figure 2C-D). CCH-induced neuronal loss was evident in the hippocampal CA1, CA2, and CA3 regions in AL BCAS mice, whereas sham controls showed normal neuronal cell bodies with distinct nuclei, nucleoli, and densely packed neurons in all three hippocampal areas (Figure 2E-F). With IF, the BCAS mice sustained less neuronal loss (Figure 2E-F). In addition, inflammatory markers such as IBA1 was significantly lower while GFAP was significantly higher in the IF BCAS group compared to AL BCAS mice (Figure S5). Finally, a marker for myelin integrity, myelin basic protein (MBP), was determined to be significantly higher in the IF BCAS group in comparison to AL BCAS mice (Figure S5).

### Altered global DNA methylation landscape under CCH is further modified with prophylactic intermittent fasting (IF)

**Figure 3:**
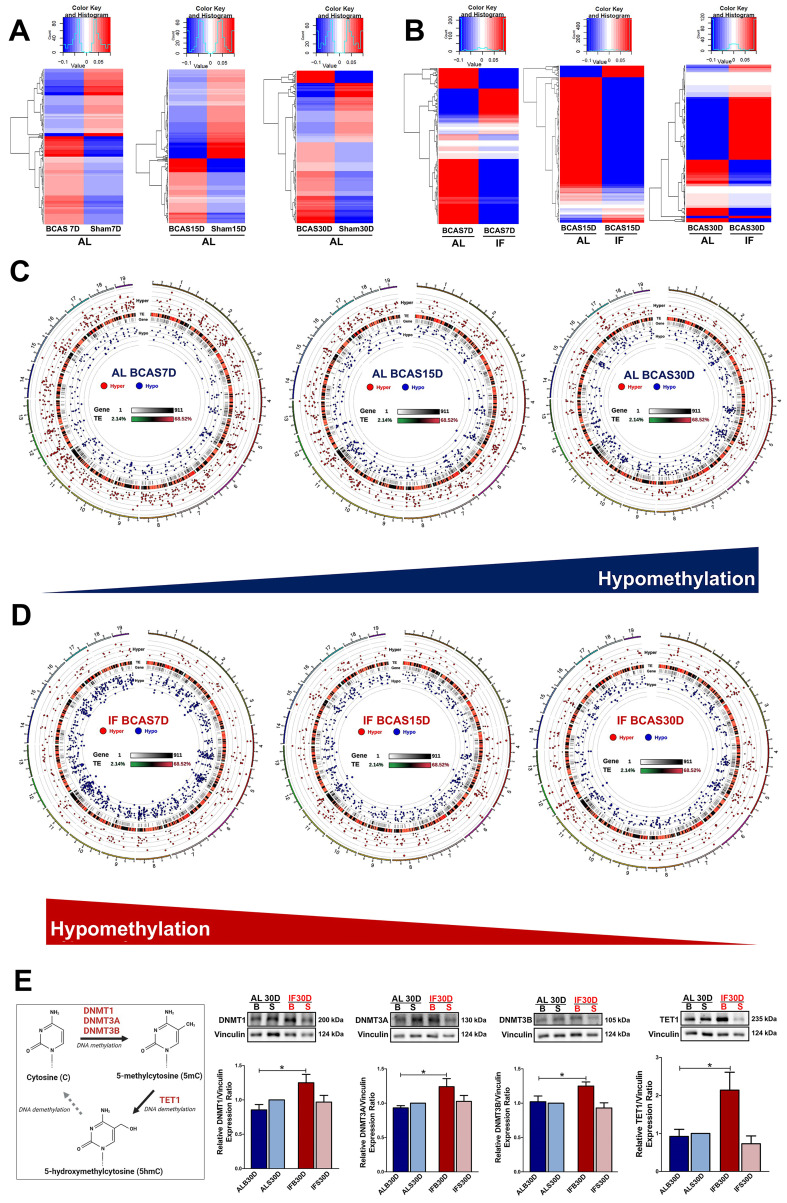
** Intermittent fasting (IF) modulates aberrant changes in the global DNA methylation landscape under chronic cerebral hypoperfusion (CCH).** (A) Heatmap of differentially methylated genes in the ad libitum (AL) CCH mice (BCAS) compared to the respective AL control mice (Sham) at the corresponding timepoints of 7, 15 and 30 days. Global changes in the gene methylation patterns were observed to deviate in the AL CCH mice compared to its respective Sham controls. (B) Heatmap of differentially methylated genes in the IF CCH mice compared to the respective AL CCH mice at the corresponding timepoints of 7, 15 and 30 days. The global trends in the gene methylation patterns was consistently distinct between the two groups as represented by the heatmap. Hypomethylated genes are indicated in blue while hypermethylated genes in red. The colour scale represents the mean methylation levels. (C) The circos representation graphs of the AL CCH mice across the 3 different timepoints showed a temporal increase in hypomethylated status as represented by the blue dots in the internal rim of the graphs. The methylation status of the differentially methylated genes was compared to the methylation levels of the respective time-specific AL Sham group. (D) The circos representation graphs of the IF CCH mice showed a temporal decrease in hypomethylated status with decreasing number of blue dots represented in the internal rim of the graphs. (E) A simplified diagram to capture the DNA methylation process and expression of DNA methyltransferases and TET1. Representative immunoblots and quantification illustrating significantly higher levels of DNA methyltransferases (DNMT1, DNMT3A, DNMT3B) and TET1 protein abundance in the IF CCH mice compared to the AL CCH mice and their respective Sham mice at the 30-day timepoint. The relative protein expression was all normalised to AL Sham samples. Vinculin was used as a loading control. Data are represented as mean ± SD. n=5-7 mice in each experimental group. Unpaired t-test, Bonferroni correction, *p<0.05 when compared with ALB30D.

**Figure 4:**
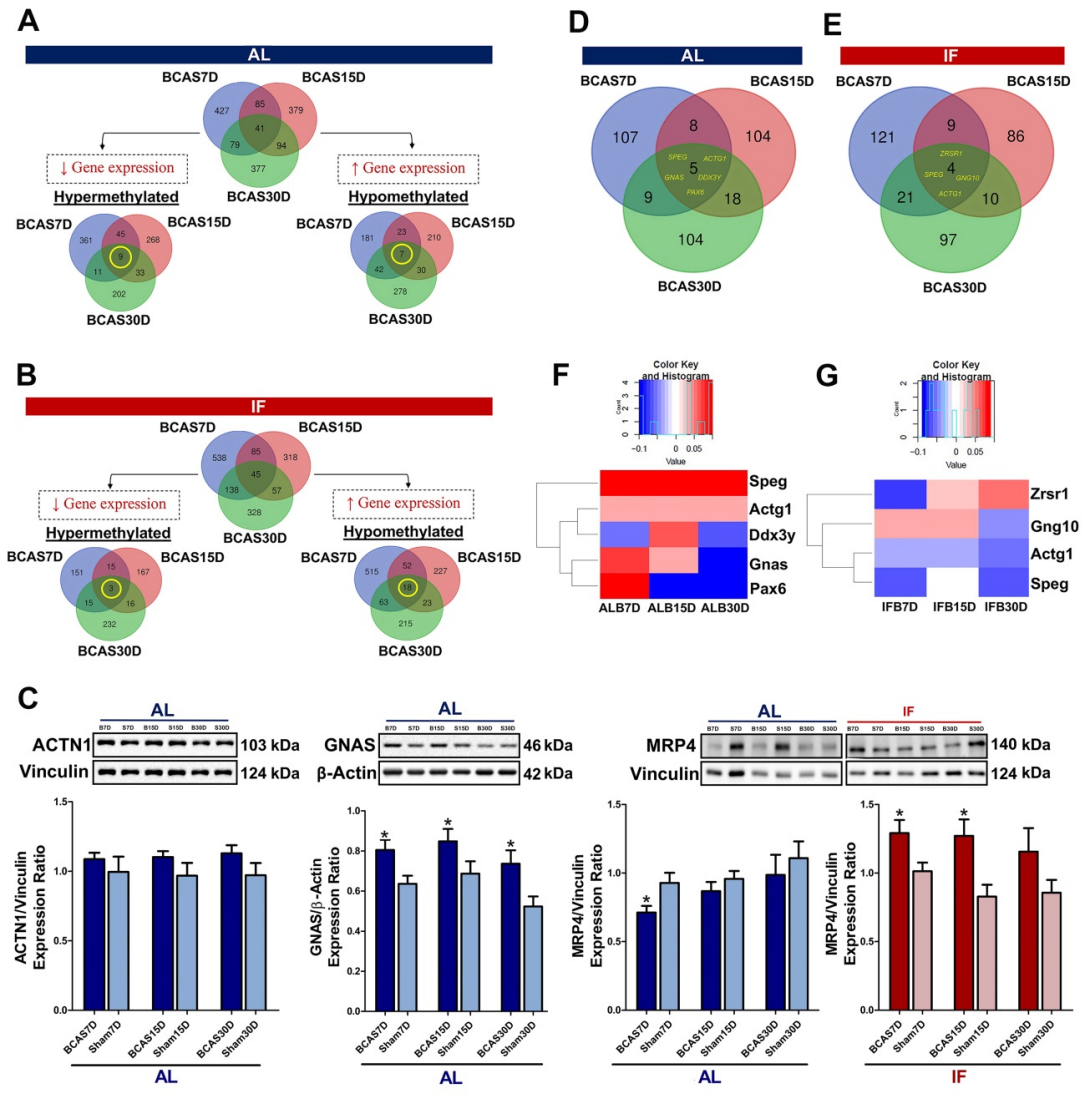
** Intermittent fasting (IF) suggests reversal of DNA methylation status in the promoter region under chronic cerebral hypoperfusion (CCH).** The methylation status of the differentially methylated genes were compared to the methylation levels of the respective time specific IF Sham group. Venn diagram of differentially methylated genes across all gene regions in the AL BCAS mice compared to AL Sham mice (A) and in the IF BCAS mice compared to AL BCAS mice (B) with respect to their corresponding Sham groups. The breakdown of specific methylation trends of differentially methylated genes were represented in Venn diagrams. A total of 9 hypermethylated genes and 7 hypomethylated genes were identified to be overlapping across all three timepoints in the AL BCAS mice and a total of 3 hypermethylated and 18 hypomethylated genes were identified to be overlapping across all three timepoints in the IF BCAS mice. (C) Representative immunoblots and quantification of overlapping differentially methylated genes to validate findings in DNA methylation sequencing. This includes protein abundance of ACTN1 in AL CCH compared to AL Sham mice, GNAS in AL CCH compared to AL Sham mice, MRP4 in AL CCH compared to AL Sham mice and IF CCH compared to IF Sham mice. Vinculin and β-actin were used as loading controls. Data are represented as mean ± SD. n=5-7 mice in each experimental group. Unpaired t-test, Bonferroni correction, *p<0.05 compared to ALB30D. Analysing specific differentially methylated genes in the promoter region by presenting the spread in the form of a Venn diagram in the (D) AL BCAS mice compared to the AL Sham mice and (E) IF BCAS mice compared to the AL BCAS mice. (F) 5 differentially methylated genes were identified to be overlapping across three different timepoints in the AL BCAS mice. The methylation levels of the 5 genes were illustrated in the form of a heatmap to show its temporal regulation which was present in Ddx3y, Gnas and Pax6. (G) 4 differentially methylated genes were identified to be overlapping across the three different timepoints in the promoter region of the IF BCAS mice. The methylation levels of the 4 genes were illustrated in the form of a heatmap to show the temporal regulation which was present in Zrsr1, Gng10, Actg1 and Speg.

To study the DNA methylation landscape under CCH, RRBS was performed and the data was analysed (Figure 3). The global DNA methylation pattern in the AL BCAS mice exhibited distinct deviation from the respective sham controls as observed in the heatmap plotted for DMGs (Figure 3A). This deviance in differential methylation from the control group was consistently observed across all three timepoints of the AL BCAS mice. With IF, the BCAS mice showed deviance in methylation profile from the AL BCAS mice (Figure 3B). The circos representation graphs (Figure 3C) show a consistent increase in trend of hypomethylation in the genes across the three different timepoints of the AL BCAS mice. Apart from these visual evidences, there were a total of 427, 379 and 377 differentially methylated genes that were unique to the 7, 15 and 30-day timepoints of AL BCAS mice respectively (Figure 4A, Table S1). In addition, 41 genes were commonly differentially methylated across the three different timepoints. These results confirm that the global DNA methylation landscape is altered under CCH state.

In contrast to the AL-fed mice, there was a consistent decrease in trend of hypomethylation observed across all three timepoints of the IF mice subjected to CCH (Figure 3D) suggesting a reversal in methylation trends. There were a total of 538, 318 and 328 DMGs that were unique to the 7, 15 and 30-day timepoints of IF BCAS mice respectively (Figure 4B, Table S1). 45 genes were commonly differentially methylated across the three different timepoints. Hence, the DNA methylation landscape that is altered under CCH is evidenced to be further modified with the introduction of prophylactic IF.

Furthermore, to determine whether DNA methyltransferases and demethylases play a role in modulating the differential methylation of genes in a CCH patholgocial state, the abundance of DNMT1, DNMT3A, DNMT3B and TET1 were studied. All three DNMTs and TET1 showed a statistically significant increase in IF BCAS mice at the 30-day timepoint compared to the AL BCAS mice (Figure 3E). This finding highlights that DNMTs and TET1 play a role in modulating the expression of DMGs under CCH with the introduction of IF.

### Identification of all differentially methylated genes (DMGs) under CCH with intermittent fasting (IF)

We separately analysed the hyper- and hypo-methylated genes to narrow down their potential functions under CCH (Table S2). A total of 929 genes were hypermethylated and 771 genes were hypomethylated in AL conditions (Figure 4A). To analyse the temporal changes in the methylation patterns, the overlapping hyper- and hypo-methylated genes across the three timepoints under AL BCAS conditions were identified (Table S3A-B). The 9 hypermethylated genes were ATP-binding cassette sub-family C member 4 (ABCC4), Guanine Nucleotide binding protein, Alpha Stimulating (GNAS), Striated Muscle Enriched Protein Kinase (SPEG), Dynamin 3 (DNM3), Actin Gamma 1 (ACTG1), Meningioma 1 (MN1), GM20388, Insulin-like growth factor 2 receptor (IGF2R) and MDS1 And EVI1 Complex Locus (MECOM). The 7 hypomethylated genes were Genetic Suppressor Element 1 (GSE1), Guanine Nucleotide binding protein, Alpha Stimulating (GNAS), Nuclear Factor I X (NFIX), Cap Binding Complex Dependent Translation Initiation Factor (CTIF), GM20388, Cap Binding Complex Dependent Translation Initiation Factor (CUX2) and Actinin Alpha 1 (ACTN1). While the functions of these respective genes are summarised in Tables S3A and S3B, it is notable that all these genes overlapping across the three different timepoints are each associated to a neurodegenerative disease which includes but is not limited to AD, Parkinson's disease and frontotemporal dementia which highlights the disease significance present. The gene expression of ACTN1 and GNAS has been validated with the methylation status (Figure 4C).

Similarly, the hyper- and hypo-methylated genes were identified in the mice subjected to prophylactic IF in comparison to the AL-fed mice to elucidate their potential biological functions during CCH (Table S4). A total of 599 genes were hypermethylated and 1113 genes were hypomethylated (Figure 4B). The differential methylation of the overlapping genes across the three different timepoints was identified (Table S5A-B). The three hypermethylated genes were GM20388, Ephrin type-A receptor 4 (EPHOR4) and BCL6 Corepressor (BCOR). The 18 hypomethylated genes were Striated Muscle Enriched Protein Kinase (SPEG), ATP-binding cassette sub-family C member 4 (ABCC4), Cap Binding Complex Dependent Translation Initiation Factor (CUX2), Insulin-like growth factor 2 receptor (IGF2R), GM20388, Mitotic Arrest Deficient 1 Like 1 (MAD1L1), Genetic Suppressor Element 1 (GSE1), Actin Gamma 1 (ACTG1), Zinc finger DHHC domain-containing protein 18 (ZDHHC18), Calmodulin Binding Transcription Activator 1 (CAMTA1), Endothelin Converting Enzyme 1 (ECE1), Nucleus Accumbens-associated protein 2 (NACC2), Disks large-associated protein 2 (DLGAP2), Arachidonate 5-lipoxygenase-activating protein (ALOX5AP), PEAK1-related kinase-activating pseudokinase 1 (PRAG1), Fibroblast growth factor receptor 2 (FGFR2), Tensin 2 (TNS2), and RAS P21 Protein Activator 3 (RASA3). The functions of these genes overlapping across the three different timepoints are summarised (Tables S5A-B). It is notable that most of the genes are associated to dementia or other neurodegenerative diseases based on literature evidence. The gene expression of MRP4 has been validated with the methylation status (Figure 4C).

### Prophylactic intermittent fasting (IF) suggests reversal of methylation status in the promoter region under CCH

To study the effect of IF on the DNA methylation landscape, the differentially methylated genes (DMGs) were analysed under both a CCH pathological state and when subjected to prophylactic IF. For the same purpose, the DMGs in the promoter region were specifically studied as they are crucial regulators of gene expression (Table S6). A total of 355 DMGs were identified in the CCH state, of which 107, 104 and 104 DMGs that were unique to the 7, 15 and 30-day timepoints respectively and 5 DMGs overlapped across the three different timepoints (Figure 4D). A total of 348 DMGs were identified in the mice subjected to prophylactic IF as compared to the AL-fed mice under CCH. Of these DMGs, 121, 86 and 97 DMGs were unique to the 7, 15 and 30-day timepoints respectively and 4 DMGs overlapped across the three timepoints (Figure 4E).

The overlapping DMGs were further analysed for their methylation status to observe temporal regulation, if any (Figure 4F-G). The 5 overlapping DMGs identified under CCH are Striated muscle Preferentially Expressed protein kinase (SPEG), Acting Gamma 1 (ACTG1), Dead-Box Helicase 3 Y-linked (DDX3Y), Guanine Nucleotide binding protein, Alpha Stimulating (GNAS) and Paired box 6 (PAX6) where the first is associated with mild cognitive impairment and the latter are all associated with AD. SPEG is a marker for differentiated vascular smooth muscle cells where it regulates growth and differentiation. ACTG1 provides instruction for making gamma-actin which is responsible for force transduction and transmission in muscle cells. DDX3Y is an ATP-dependent RNA helicase which plays a role in RNA metabolism and translational regulation. GNAS provides instructions for making stimulatory alpha subunit, G-protein which act as signal transmitters or switches. PAX6 is a transcription factor crucial for coordination of differentiation and proliferation. The 4 overlapping DMGs identified in the IF mice subjected to CCH state are Zinc finger (CCCH type), RNA-binding motif and serine/arginine-rich 1 (ZRSR1) that is associated with Parkinson's disease, G Protein Subunit Gamma 10 (GNG10) associated with mild cognitive impairment and AD, ACTG1 associated with AD and SPEG associated with mild cognitive impairment. ZRSR1 plays a critical role in alternative splicing, mainly in recognising U12-type introns. GNG10 is a modulator of various transmembrane signalling systems.

Analysing the methylation levels of these overlapping DMGs under a CCH pathological state and when subjected to prophylactic IF revealed temporal regulation (Figure 4F-G). The temporal regulation of the gene's methylation level is unique to each gene and in some cases (i.e. DDX3Y, GNAS, PAX6, ZRSR1) the change is significant enough to switch from a hypermethylated to hypomethylated state or vice versa. It is notable that SPEG and ACTG1 genes are present in the overlapping genes across the timepoints under the diseased state and when subjected to IF. Both these genes were hypermethylated across all three timepoints under the AL-fed state (Figure 4F). However, when subjected to IF, both these genes were observed to be hypomethylated across all three timepoints (Figure 4G) suggesting a reversal of the methylation status upon intervention with prophylactic IF.

## Discussion

The gene-environment interaction in the context of VaD has been implicated with the observation of environmental factors such as diet, stress and smoking may drive the pathogenesis of the disease 17,18. However, this implication has yet to be substantiated with molecular reasoning and underpinnings. This study, therefore, focused on the DNA methylation landscape in the brain under CCH in a mouse model of VaD. In addition, a prophylactic intervention of IF was introduced to study its modulation of the DNA methylation landscape. Proof-of-concept experiments were conducted to ensure that CCH was successfully induced in the BCAS mouse model utilised. A reduction in body weight is a distinct phenomenon in IF conditions where it is speculated to be due to lowered insulin levels and increased growth hormone release which result in catabolism of fats, thus lowering body weight 19. Under fasting conditions, the body shifts its metabolic activity from lipid synthesis and fat storage to mobilising breakdown of fats via fatty-acid oxidation and production of fatty-acid derived ketones 20. This flip in metabolic activity was observed in the IF mice as well.

While the beneficial effects of IF have been established, the role it plays in modulating the DNA methylation landscape per se, if any, is not well explored. So far, there are only a few studies that have investigated the altered DNA methylation landscape in an alternate-day fasting regimen in liver and skeletal muscle 12,21,22. However, to our knowledge, the effect of IF (16 h regimen) on the DNA methylation landscape of mouse brain under physiological conditions is not characterised. The ALWT vs IFWT analysis thus serves as evidence for an altered DNA methylation profile with the administration of prophylactic IF. The DNA methylation landscape was analysed according to three different contexts (CG, CHG and CHH). This was in view of the unknown role played by the non-canonical methylation sites (i.e. CHG and CHH contexts) which could provide insights into the modulation of gene expression 23. It is notable that the global DNA methylation favours hypomethylation. However, the abundance of DNMTs are significantly higher in IFWT mice which implies greater hypermethylation due to the addition of methyl groups. This difference could be attributed to the gene region at which the methylation occurs. Increased methylation in non-promoter regions is less likely to affect modulation of gene expression, thus resulting in a hypomethylated state despite the increase in DNMT levels. IF showed improvement in the long term retention phase of memory and better spatial retrieval memory highlighting the prolonged effect of IF on working memory, which is consistent with exisiting findings 24,25.

The CBF readings serve as an immediate indication of the reduced blood supply to the brain. In addition to serving as a validation, these proof-of-concept experiments highlighted the effect of IF at a pathophysiological level as well. The WML, for instance, is a hallmark of VaD. Persistent hypocapnia has been demonstrated to inflict damage in the caudoputamen region 26 and WML in the corpus callosum region are distinct features of VaD 27, thus explaining the focus of studying these regions. They were assessed for disarrangement of nerve fibres, marked vacuoles and/or disappearance of myelinated fibres as characterised by Wakita and colleagues (1994) 28. While the increased lesions under CCH was observed as expected, the reduced marked vacuoles in the IF mice suggested improvement in neuropathological severity. The amelioration of cognitive impairment in IF mice under CCH is evidence for the promise IF holds as a prophylactic intervention. Notably, a differential effect of reduced IBA1 levels and elevated GFAP levels were observed with the introduction of prophylactic IF. In general, astrocytes have been shown to be more resistant to ischemia than neurons and microglia 29-31. Consistent with the literature, it is observed that in our study, under AL-fed chronic cerebral hypoperfusion conditions, the GFAP levels were not significantly different from the respective sham group (i.e. ALB30D vs ALS30D) (Figure S5). However, IBA1 levels showed a significant difference between ALB30D and ALS30D groups (p<0.05; not included in the figure). Hence, it can be interpreted that the ischemic event impacts the microglia more than the astrocytes, thus resulting in intermittent fasting to lower the IBA1 levels to mitigate the damage. On the contrary, the elevated GFAP levels in the IFB30D mice compared to the ALB30D mice can be attributed to the fact that astrocyte reactivity is dynamic and has highly variable outcomes. Reactive astrocytes have been demonstrated to play a protective role under ischemic conditions 32. Studies have shown that astrocytic activation can stimulate two types of astrocytes, the A1 astrocytes which are neurotoxic and A2 astrocytes which have neuroprotective properties 33. In fact, ischemia is known to induce A2 astrocytes which in turn have been observed to induce the production of cytokines such as TNF-α. While TNF-α has been well established as a proinflammatory cytokine, it has been recently found to have an anti-inflammatory role as well. Notably, under ischemic conditions, TNF-α inhibits pro-inflammatory cytokines and it has also demonstrated the ability to stimulate development of synapses and ensuring the survival of neurons 34. IF has been shown to elicit beneficial effects which include inducing anti-inflammatory responses or attenuating pro-inflammatory responses 35, 36. Hence, the increase in GFAP levels in IFB30D mice could be due to the increase in neuroprotective A2 astrocyte phenotype.

Mapping of the DNA methylation patterns under CCH in the AL fed and IF mice provided insights into the landscape present under the diseased state versus when an intervention is established. Sorting of gene regions to be primarily from the CG context stems from the fact that CpG islands (CGIs) with high nucleotide density is where DNA methylation predominantly occurs in mammals 37. There are two main layers of findings elucidated from the DNA methylation landscape observed. One is the deviation of methylation profile in a CCH state from baseline. The other is the deviation of methylation profile in IF mice under CCH compared to the AL fed mice. While the deviation from point of comparison highlights the modulatory changes, the exact trend and its implications are determined based on the DEGs identified.

The analysis of overlapping DEGs in the hypermethylated and hypomethylated states provides understanding of the functional implications regarding the changes in the methylation profile. Both AL-fed and IF mice which underwent CCH had overlapping DEGs respectively which were all associated to at least one neurodegenerative disease, specifically under the umbrella term of dementia. This includes diseases like AD, Frontotemporal dementia (FTD), Parkinsonism, VaD and mild cognitive impairment (MCI). The presence of dementia-related genes in this mouse model of CCH serves as preliminary validation to mimic the conditions observed in patients. While there are different permutations of analysis that can be performed, the overlapping DEGs were first studied as they would facilitate the understanding of temporal regulation of the genes. In addition, the sorting of the overlapping DEGs is important to distinguish the hypermethylated and hypomethylated genes. Knowing the functions of these genes is more important as they provide an understanding of their role in the system involved. For instance, *ABCC4* is hypermethylated in AL-fed mice and hypomethylated in IF mice when subjected to CCH. *ABCC4* plays a key role in the vascular system by ensuring cyclic adenosine monophosphate homeostasis 38. Under physiological conditions, *ABCC4* functions as an active transporter at the membrane to eliminate drugs and endogenous molecules and when its expression is inhibited, promotion of cardiac hypertrophy and induction of thrombopathy have been observed 38. Hence, the hypomethylation of *ABCC4* in IF mice under CCH in contrast to the hypermethylated state in AL-fed mice suggests possible restoration of function with increased gene expression. Similarly, the gene *SPEG* is hypermethylated in AL-fed mice and hypomethylated in IF mice under CCH, which translates to a reduction and and increased gene expression respectively. It has been shown that *SPEG* gene is crucial for cardiac development and function and downregulation of its expression is observed in human failing hearts and cardiac dysfunctions in mouse models 39. Moreover, genes such as *ECE1* and *FGFR2* which are hypomethylated in IF mice under CCH have been shown to confer neuroprotection. A genetic association study showed that *ECE1* is expressed in human cerebral cortex and that it potentially confers protection against AD 40. *FGFR2*, on the other hand, has been established to play a crucial role in neuroprotection, nrogenesis, nerve repair and hippocampal learning 41-43. Hence, this trend of differential methylation between AL-fed and IF mice highlights the promise IF holds in countering the methylation changes induced under CCH.

It is notable that in both these above-mentioned set of analyses, the hypo- and hypermethylated status were contributed by the cumulative methylation levels of the different gene regions such as exons, introns, promoters and transcription start and end sites. Hence, there is a need for a focused approach to fully comprehend the effect of the methylation changes on the expression of the respective genes. In particular, methylation at the promoter region was investigated as their functional importance is emphasised by the CpG islands associated with the promoter region being conserved in both humans and mice and with approximately 70% of the gene promoters situated within CpG islands 44. *SPEG* and *ACTG1* genes were present in both AL-fed and IF mice under CCH which allows for analysis of two confounders- temporal regulation and the effect of prophylactic intervention. The *ACTG1* gene's function is critical in cell movement and maintaining the cytoskeleton such as playing an important role synapse morphology 45. Moreover, *ACTG1* expression has been reported to be correlated with the Montreal Cognitive Assessment score for AD patients and has been found to be associated with reduced levels in the blood and brain tissue of mice with AD 45. The completely opposing methylation status, which is temporally consistent, for the *SPEG* and *ACTG1* genes between the AL and IF mice highlights the potential of IF to reverse the methylation landscape. As for the *ZRSR1* gene, a gender-based mouse study was suggestive of the role it played in controlling behaviour through the hypothalamic cell network (46). In particular, it was shown that the *ZRSR1* mutant male mice exhibited increased social interaction in contrary to the female mutant mice that showed aggressive behaviour. With an initial hypomethylation but subsequent temporal switch to a hypermethylated status in the 15 and 30-day timepoints after cerebral hypoperfusion highlights the downregulation of *ZRSR1* expression and hence improved behavioural control. *GNG10* expression has been associated with cognitive decline in a gene-based association study but its exact role remains unexplored 47.

DNMTs, specifically DNMT1, DNMT 3A and 3B, have a role to play in the methylation status of the above discussed genes and hence were investigated. DNMT1 serves as the main DNA methyltransferase in mammalian cells and is highly conserved between humans and mice. Several studies have reported observations of a global decrease in DNA methylation levels and hence an associated decrease in DNMT expression levels in the brains of humans and mice in the context of aging and neurodegenerative diseases 48, 49. Apart from the observations being replicated in this study with a temporal decrease in DNMTs in the AL-fed mice, a reversing trend was observed in the IF mice. The increase in DNMTs in the IF mice was pronounced at the 30-day timepoint after cerebral hypoperfusion highlighting the promise IF holds as a prophylactic intervention.

Overall, this study demonstrates for the first time that BCAS-induced CCH altered the DNA methylation landscape in the brain of a suitable mouse model of VaD. Furthermore, the potential of prophylactic IF in modulating the DNA methylation landscape under physiological conditions and pathological conditions of CCH was demonstrated for the first time in this study as well. In fact, IF has shown its potential to slow down the disease progression in CCH by modulating the DNA methylation landscape (Figure S6). Hence, the addition of IF as a potential prophylactic intervention and the protection against the aberrant changes in the DNA methylation landscape provides value and novelty to the study. Due to the nature of the study involving sequencing data with a wealth of information, there is still much to be explored in terms of gene clustering, narrowing down of pathways and identifying key genes that play a pivotal role in the methylation changes incurred. For instance, it is evident that the oxidative stress induced by the reduced CBF has been shown to stimulate the production and release of pro-inflammatory mediators and initiate neuroinflammation in VaD 50. Neuroinflammation is an important area of focus for an upcoming study from our group. Nevertheless, this study has stimualted a new avenue to unravel the immense potential IF holds as a prophylactic intervention by modulating the DNA methylation landscape in a CCH mouse model of VaD.

## Supplementary Material

Supplementary figures and tables.

## Abbreviations

VaD: vascular dementia
CCH: chronic cerebral hypoperfusion
IF: intermittent fasting
BCAS: bilateral common carotid artery stenosis
RRBS: reduced representation bisulfite sequencing
WML: white matter lesion
DNA: deoxyribonucleic acid
RNA: ribonucleic acid
NACLAR: national advisory committee for laboratory animal research
ARRIVE: animal research reporting *in vivo* experiments
AL: ad libitum
CCA: common carotid artery
CBF: cerebral blood flow
PBS: phosphate buffered saline
CCD: charged couple device
LFB: luxol fast blue
dsDNA: double-stranded DNA
DMR: differentially methylated region
GO: gene ontology
KEGG: Kyoto Encyclopedia of Genes and Genomes
PPI: protein-protein interaction
DNMT: DNA methyltransferase
MBP: myelin basic protein
TBST: tris-buffered saline with 0.1% Tween® 20 detergent
HRP: horseradish peroxidase
CR: calorie restriction
WT: wildtype
TET: ten- eleven translocation 1
DMG: differentially methylated gene
GFAP: glial fibrillary acidic protein
IBA1: ionized calcium binding adaptor molecule 1
ABCC4: ATP-binding cassette sub-family C member 4
GNAS: guanine nucleotide binding protein, alpha stimulating
SPEG: striated muscle enriched protein kinase
DNM3: dynamin 3
ACTG1: actin gamma 1
MN1: meningioma 1
IGF2R: insulin- like growth factor 2 receptor
MECOM: MDS1 And EVI1 Complex Locus
GSE1: genetic suppressor element 1
NFIX: nuclear factor I X
CTIF: cap binding complex dependent translation initiation factor
CUX2: cap binding complex dependent translation initiation factor
ACTN1: actinin alpha 1
AD: Alzheimer's disease
PD: Parkinson's disease
EPHOR4: ephrin type-A receptor 4
BCOR: BCL6 Corepressor
MAD1L1: mitotic arrest deficient 1 Like 1
ZDHHC18: zinc finger DHHC domain-containing protein 18
CAMTA1: calmodulin binding transcription activator 1
ECE1: endothelin converting enzyme 1
NACC2: nucleus accumbens-associated protein 2
DLGAP2: disks large-associated protein 2
ALOX5AP: arachidonate 5-lipoxygenase-activating protein
PRAG1: PEAK1-related kinase-activating pseudokinase 1
FGFR2: fibroblast growth factor receptor 2
TNS2: tensin 2
RASA3: RAS P21 protein activator 3
MRP4: multidrug resistance protein 4
DDX3Y: dead-box helicase 3 Y-linked
PAX6: paired box 6
ZRSR1: zinc finger (CCCH type), RNA-binding motif and serine/arginine-rich 1
GNG10: G protein subunit gamma 10
TNF-α: tumour necrosis factor alpha
CGI: CpG island
DEG: differentially expressed genes
