# Supplementary material for: Time-restricted feeding modulates the DNA methylation landscape, attenuates hallmark neuropathology and cognitive impairment in a mouse model of vascular dementia
Source: Theranostics. 2022 Mar 21;12(7):3007–23. doi: 10.7150/thno.71815 (PMC9065201; doi:10.7150/thno.71815)
Supplement: Supplementary file 1 — Supplementary figures and tables. [file thnov12p3007s1.pdf]

Time-restricted feeding modulates the DNA methylation landscape, attenuates hallmark neuropathology and cognitive impairment in a mouse model of vascular dementia

Sharmelee Selvaraji[1,2,*], Motakis Efthymios[3,4,5], Roger Sik Yin Foo[3,4,5], David Y. Fann[6,7,8], Mitchell Kim Peng Lai[1], Christopher Li Hsian Chen[1,9], Kah Leong Lim[10], Thiruma V. Arumugam[11,12,*]

[1] Memory Aging and Cognition Centre, Department of Pharmacology, Yong Loo Lin School of Medicine, National University of Singapore, Singapore.
[2] Integrative Sciences and Engineering Programme, NUS Graduate School, National University of Singapore.
[3] Cardiovascular Research Institute, National University Health System, Singapore.
[4] Cardiovascular Translational Research Programme, National University of Singapore, Singapore.
[5] Genome Institute of Singapore, Agency for Science, Technology and Research, Singapore.
[6] Department of Biochemistry, Yong Loo Lin School of Medicine, National University of Singapore, Singapore.
[7]Healthy Longevity Translational Research Program, Yong Loo Lin School of Medicine, National University of Singapore, Singapore.
[8]Centre for Healthy Longevity, National University Health System (NUHS), Singapore.
[9] Department of Psychological Medicine, Yong Loo Lin School of Medicine, National University of Singapore, Singapore.
[10] Lee Kong Chian School of Medicine, Nanyang Technological University, Singapore.
[11] School of Pharmacy, Sungkyunkwan University, Suwon, Republic of Korea.
[12] Centre for Cardiovascular Biology and Disease Research, Department of Microbiology, Anatomy, Physiology and Pharmacology, School of Agriculture, Biomedicine and Environment, La Trobe University, Bundoora, VIC, Australia.

*Correspondence: Thiruma V. Arumugam, Department of Microbiology, Anatomy, Physiology and Pharmacology, School of Agriculture, Biomedicine and Environment, La Trobe University, Bundoora, VIC, Australia. E-mail: g.arumugam@latrobe.edu.au; Sharmelee Selvaraji, Memory Aging and Cognition Centre, Department of Pharmacology, Yong Loo Lin School of Medicine, National University of Singapore, Singapore. E-mail: e0358056@u.nus.edu

Supplementary Figures

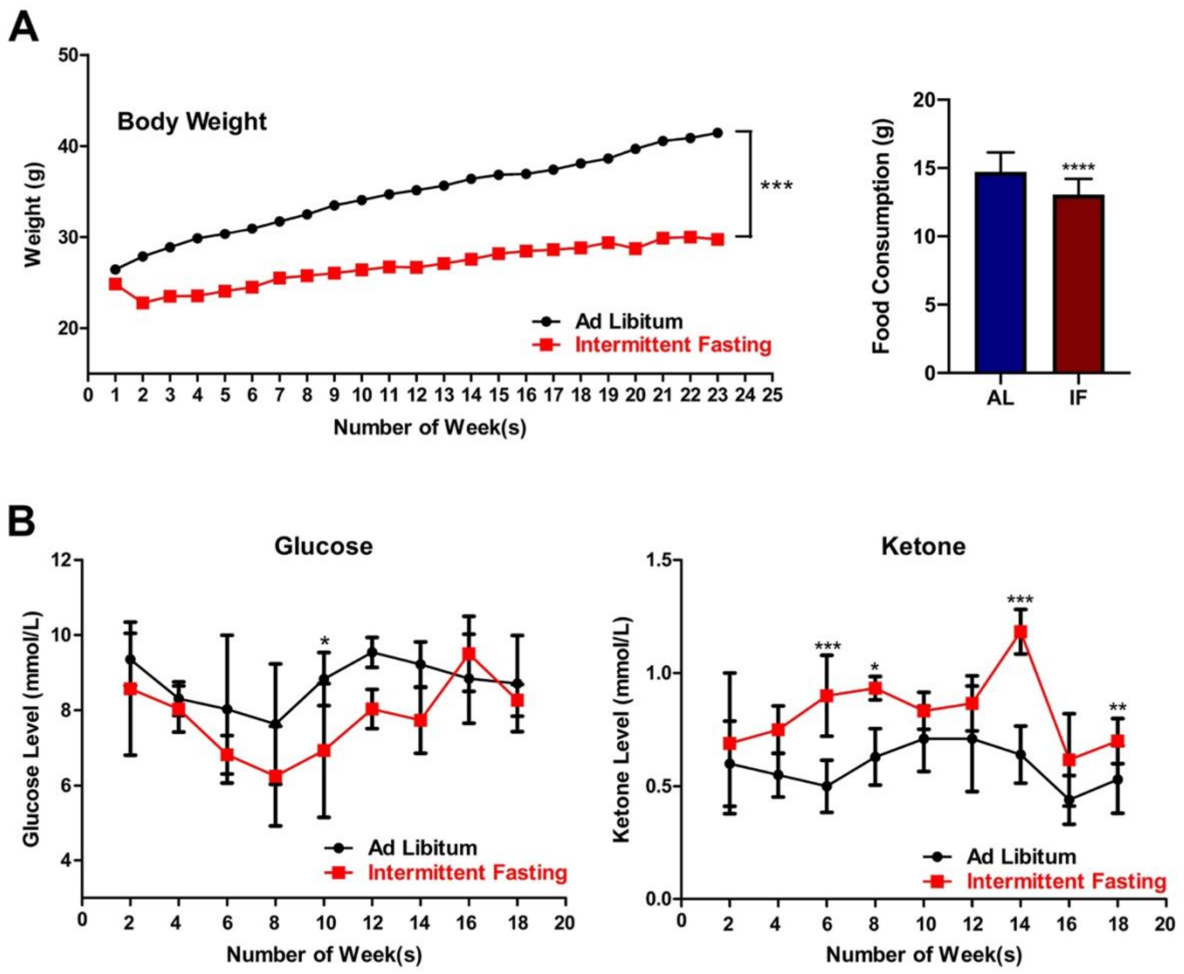

Figure S1. Effects of intermittent fasting (IF) on physiological parameters under chronic cerebral hypoperfusion (CCH). (A) Mean weekly body weight measurements. IF mice had a significantly lower body weight compared to the AL mice. A significant reduction in food consumption of IF mice compared to AL mice was apparent although it was below the range defined to be calorie restriction. (B) Mean bi-weekly blood glucose and ketone level analyses. A significant increase in ketone levels was observed in the IF mice compared to the AL mice where a metabolic switch from glucose to ketone in the IF mice was notable. Unpaired t-test, Bonferroni correction, *p<0.05, **p<0.01, ***p<0.001 when compared with AL.

A

Number of hypermethylated and hypomethylated gene regions in CG, CHG, CHH context respectively.

	CG	CHG	CHH
Hypermethylated	493	30	130
Hypomethylated	2459	80	235
Total	2952	110	365

Number of hypermethylated and hypomethylated **promoter genes** in CG, CHG, CHH context respectively.

	CG	CHG	CHH
Hypermethylated	24	1	12
Hypomethylated	167	5	14
Total	192	6	26

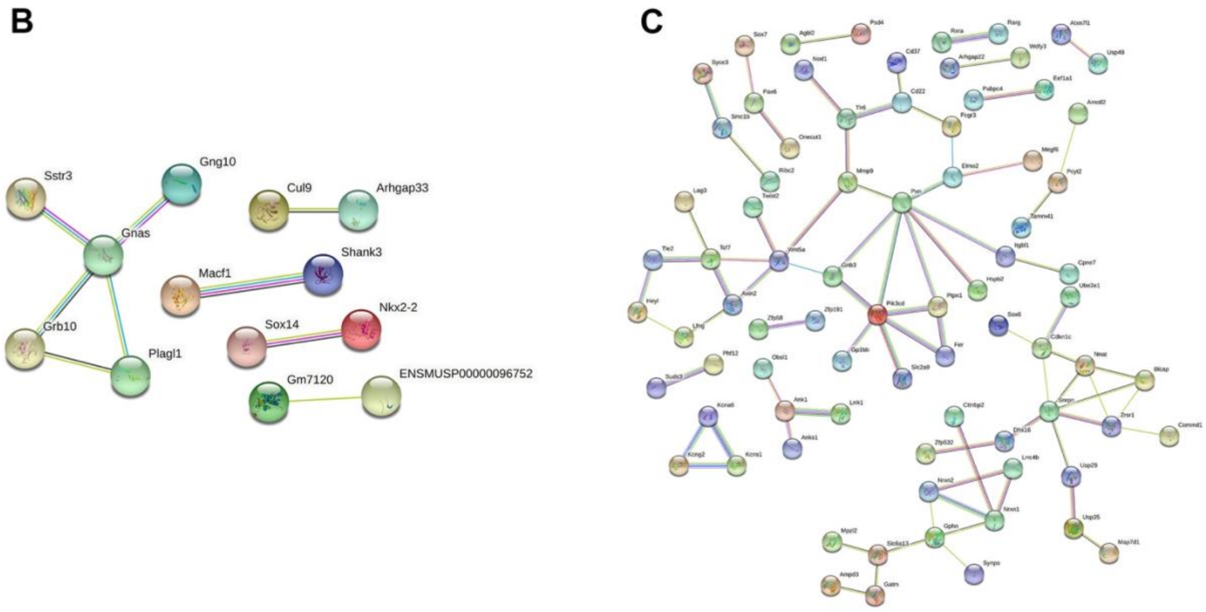

Figure S2. Potential biological functional link between hyper- and hypo-methylated promoter genes in IFWT compared to ALWT mice. (A) Summary of number of hypo- and hyper-methylated gene regions in CG, CGH and CHH contexts respectively in all regions and in promoter region respectively. (B-C) Clustering of genes by protein-protein interaction networks using STRING database for all the hyper- and hypomethylated promoter genes in the CG context.

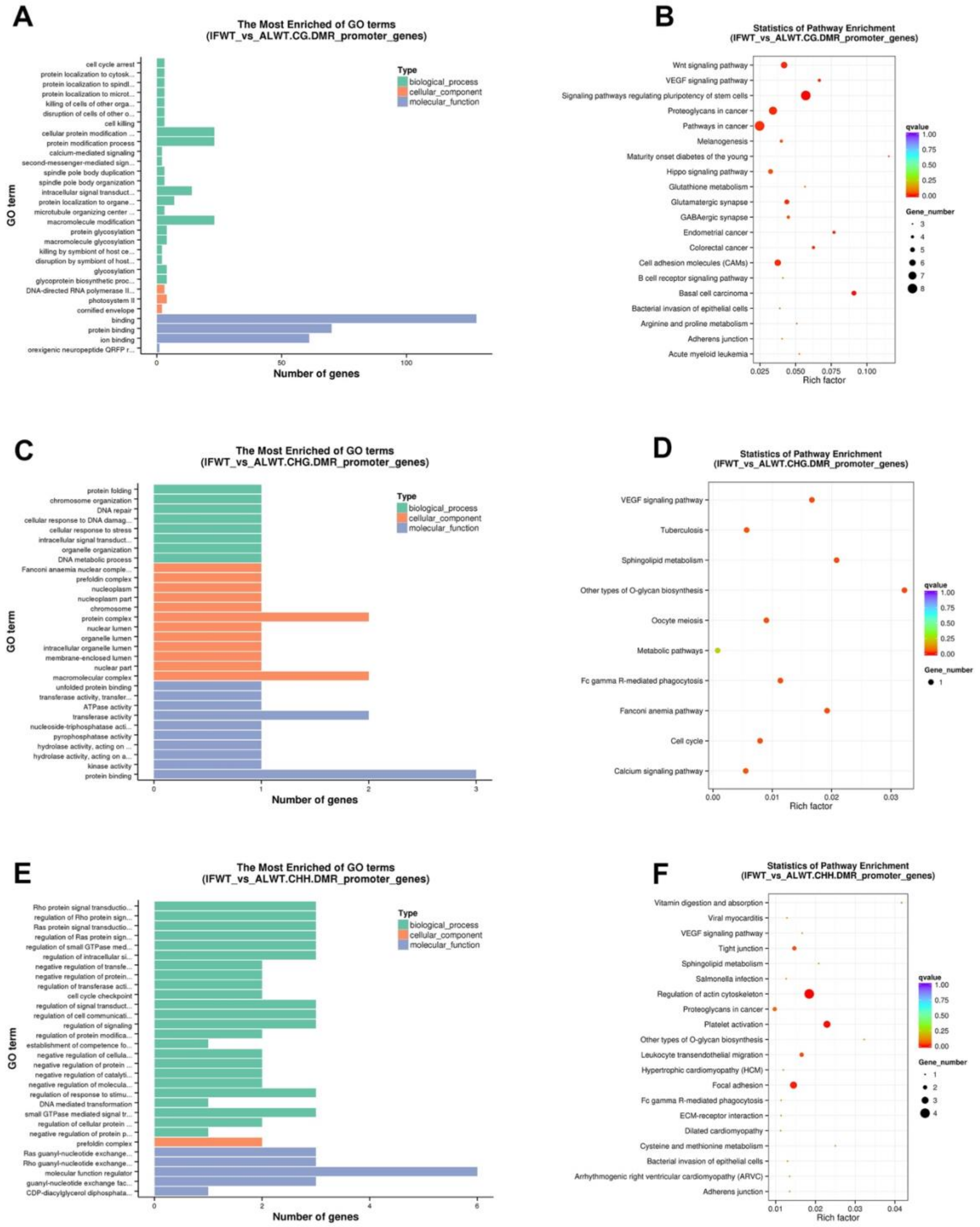

Figure S3. Potential biological functions of differentially methylated genes in the IFWT mice. (A-B) GO term analysis and KEGG pathway analysis for differentially methylated genes in the CG context, (C-D) CHG context and (E-F) CHH context respectively.

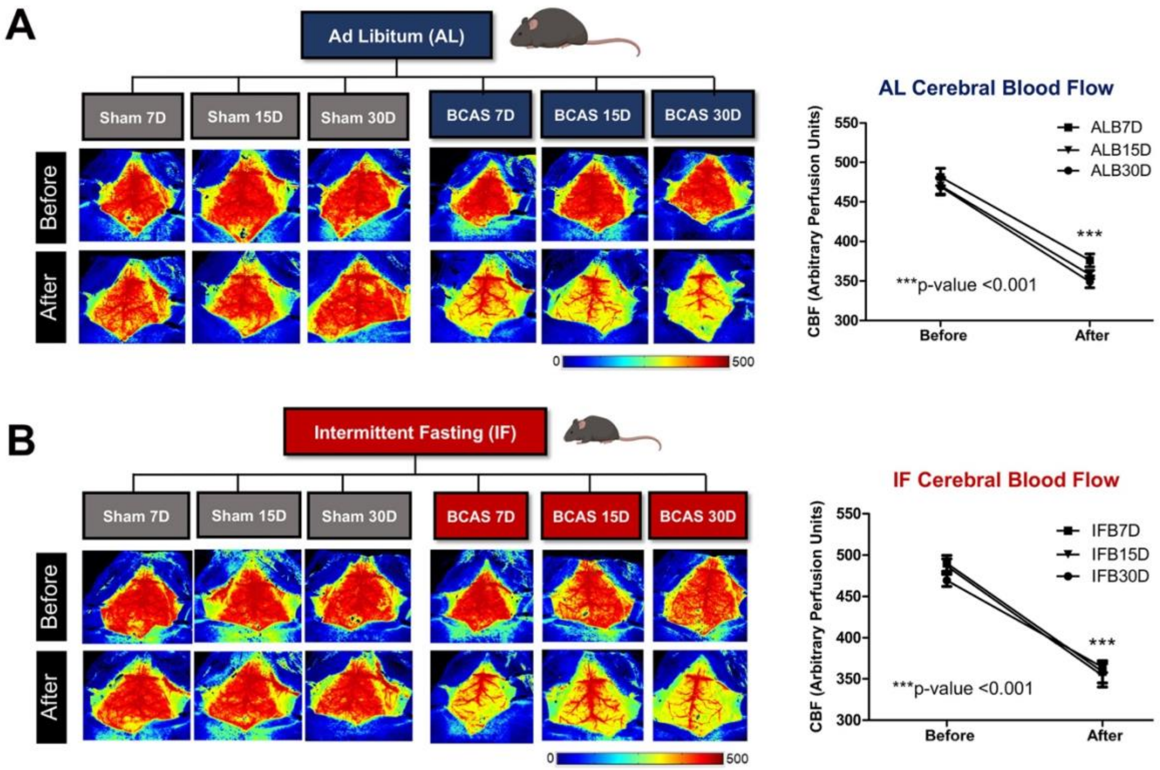

Figure S4. Reduction in cerebral blood flow (CBF) under chronic cerebral hypoperfusion (CCH). (A-B) Representative images of cerebral blood flow (CBF) data before and immediately after BCAS surgery for AL CCH (ALBCAS) and IF CCH (IFBCAS) mice respectively. Quantification of the CBF data for the respective timepoints are significantly decreased upon BCAS surgery when compared to the respective sham controls. The relative CBF levels are presented in arbitrary perfusion units. n=8-10 mice per experimental group were used for CBF analyses. ***p<0.001 when compared with AL, two-way analysis of variance (ANOVA), Bonferroni post-hoc test. All values are mean ± SD.

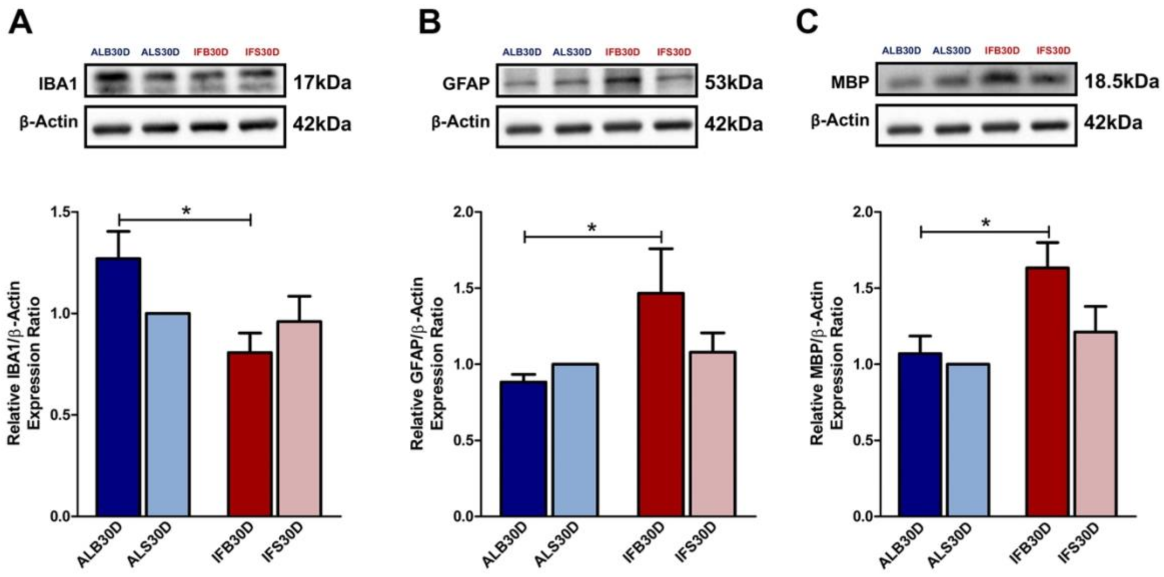

Figure S5. Effects of intermittent fasting (IF) on glial activation and white matter integrity in the cortex under chronic cerebral hypoperfusion (CCH). Representative immunoblots and quantification illustrating significantly reduced levels of IBA1 (Microglial marker) and significantly increased levels of GFAP (Astrocyte marker) and MBP (myelin integrity marker) in the IF CCH mice compared to the AL CCH mice and their respective Sham mice at the 30-day timepoint. The relative protein expressions are all normalised to AL Sham samples. β-actin was used as a loading control. Data are represented as mean ± SD. n=5-7 mice in each experimental group. Unpaired t-test, Bonferroni correction, *p<0.05 when compared with ALB30D.

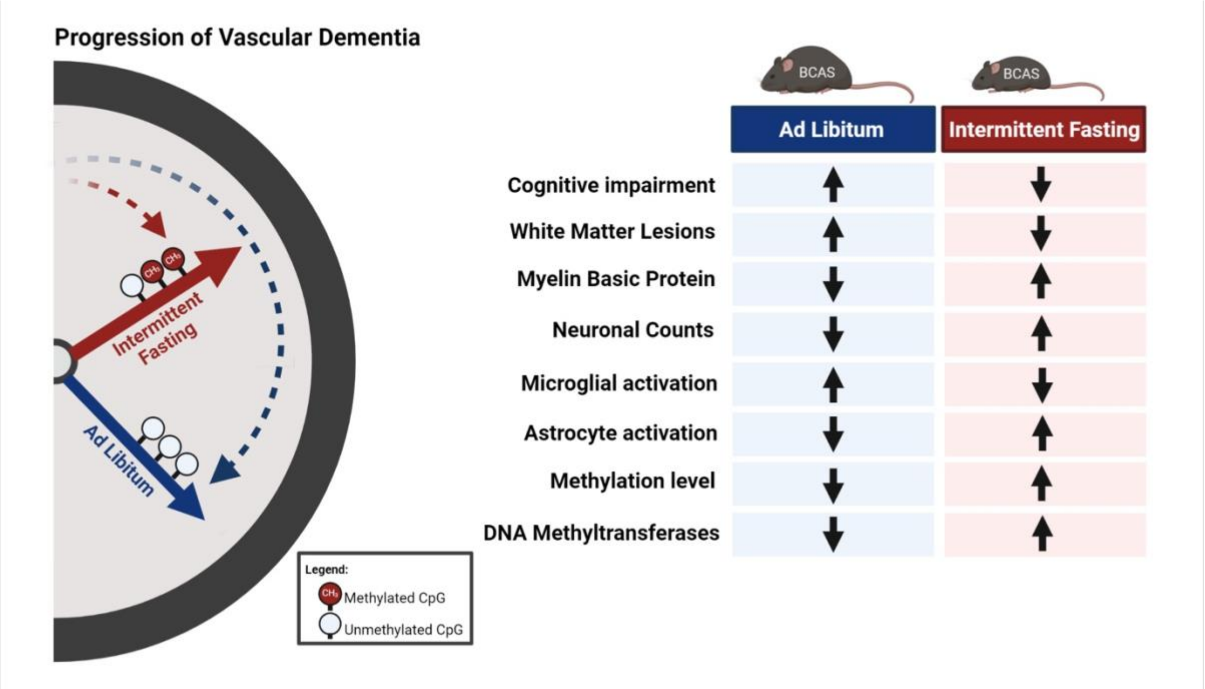

Figure S6. Summary figure illustrating the effects of intermittent fasting (IF) in mitigating the progression of vascular dementia (VaD). VaD results in a chronic cerebral hypoperfusion (CCH) state that is induced in mice through bilateral common carotid artery stenosis (BCAS) surgery. The progression of VaD is illustrated as a DNA methylation clock where the ad libitum (AL) dietary regimen is shown to be progressing faster than the IF dietary regimen 'hand' of the clock. The methylated groups (CH_3 groups) on the IF 'hand' of the clock represents the observation of increased hypermethylation as opposed to the AL condition as per the global DNA methylation sequencing data under CCH. The specific changes to cognitive impairment, neuropathological alterations and DNA methylation landscape in the AL BCAS mice as opposed to the IF BCAS mice were briefly summarised in the table. CpG refers to CG nucleotide rich sites.

Table S1. Full list of differentially methylated genes across the three different timepoints under AL CCH and IF CCH conditions respectively.

AL BCAS7D	AL BCAS15D	AL BCAS30D	IF BCAS7D	IF BCAS15D	IF BCAS30D
Abat	Abcc4	Abca16	Abcc12	Abat	Aatk
Abcc4	Abi2	Abcb9	Abcc4	Abcc4	Abca16
Abl1	Abi3	Abcc4	Abi3	Abhd8	Abca4
Ablim1	Acsf2	Abi3	Abl1	Acbd6	Abcc4
Actg1	Acss1	Acer2	Ablim3	Actg1	Abi3
Actn1	Actg1	Acin1	AC149090.1	Adam12	Acap2
Acvr1	Actn1	Acot1	Acbd4	Adamts1	Acsf2
Adam12	Acvr2b	Acta1	Acot1	Adamts20	Actg1
Adam33	Ada	Actg1	Acsf3	Adra1b	Actn1
Adamts17	Adam32	Actn1	Acss1	Agap3	Adamts14
Adap2	Adamts1	Acvr2b	Acta1	Ahdc1	Adamts3
Adcy4	Adamts20	Adam11	Actg1	AI854703	Adcy5
Adgrb2	Adamtsl3	Adcy5	Actn1	Ak7	Adgrl4
Adora1	Adcy6	Adgrb2	Adam12	Akap6	Adnp2
Adora2a	Adra1b	Adgrg6	Adamts17	Akap9	Adra1d
Adra1b	Adra1d	Aff3	Adamts2	Alox5ap	Aff3
Adra2c	Aen	Afmid	Adamts6	Amotl2	Afmid
Ahdc1	AI464131	Agap1	Adamtsl2	Angel2	Agap1
Akap6	Ajap1	Agap2	Adamtsl5	Ank	Agap2
Aldh18a1	Akap6	Agbl4	Adcy2	Ank2	Akap6
Alk	Akap7	Ahdc1	Adcy5	Ankrd13b	Akr1d1
Alox5ap	Akap9	AI464131	Adgra2	Ankrd44	Alk
Ank2	Akp3	Ak5	Adgrl4	Anks1b	Alox5ap
Ank3	Aldh8a1	Akr1b8	Adra1b	Antxr2	Anapc4
Anks1	Als2	Akt1	Afap1	App	Ank2
Anks6	Amn	Aldh1l1	Aff3	Aqp6	Anks1b
Ano4	Amotl2	Alk	Agap2	Arhgap27	Apba2
Anp32a	Ank3	Alox5ap	Agap3	Arhgdia	Apbb1
Ap3b1	Ankrd34b	Amn	Agbl5	Arhgef28	Appl2
Apbb2	Anks1b	Ank	Agt	Arx	Arhgap21
Apoh	Ano2	Ankrd35	Ahdc1	Asap1	Arhgap31
Arhgap15	Ano6	Ap3b2	Ahnak2	Asic1	Arhgap39
Arhgap22	Ap1b1	Aqp1	Akap1	Atf3	Arhgap9
Arhgef10	Ap1m2	Arhgap21	Alk	Atp9a	Arhgef18
Arhgef2	Apba2	Arhgap8	Alox5ap	B3gat2	Arhgef28
Arhgef28	Arhgap21	Arhgef10	Amn	B3gnt2	Arid1b
Arx	Arhgap26	Arhgef28	Amotl1	B4galt2	Arid2
Asap1	Arhgef10	Arhgef3	Ank	Bahcc1	Arpp21
Atf6b	Arhgef28	Arid2	Ankrd6	Bahd1	B3gat2
Atp6v1g2	Arl15	Arl14	Anks1b	Basp1	B3gnt3
Azi2	Arrdc5	Arl14epl	Anp32a	BC017158	B4galnt4
B230104I21Rik	Aspg	Arrdc5	Anxa8	Bcl6	Bach2

B3gat2	Atg7	Arsg	Arhgap15	Bcl9l	Barhl1
Bach2	Atn1	Asic1	Arhgap22	Bcor	BC067074
Bahcc1	Atp4a	Atg7	Arhgap23	Bdh1	Bckdhb
Bahd1	Atp6v0a1	Atp11b	Arhgap27	Bin3	Bcl11b
Barhl1	Atp6v0a2	Atp1a3	Arhgap28	Bmp2k	Bcl2
Barx2	Atp9a	Atp5o	Arhgap31	Bop1	Bcor
Basp1	Atxn1	Atxn1	Arhgap39	Bptf	Begain
Bcam	Axin2	B3gat2	Arhgap9	Bri3	Best2
Bcar3	B3gat2	B3gnt3	Arhgef10	Btbd9	Bop1
Bckdha	B3gnt3	Bag3	Arhgef10l	C530008M17Rik	Brd1
Bcl11b	B3gnt8	Banp	Arhgef17	C77080	Brd2
Bcor	B4galt1	Baz2b	Arhgef2	Cacna1a	Bst2
Begain	B4galt2	BC048507	Arhgef40	Cacnb4	C2cd4c
Bicc1	Banp	Bcan	Arid1b	Cacng8	Cabin1
Bicra	Basp1	Bcar3	Arrdc5	Cadm1	Cacna1a
Blcap	BC017158	Bcl11b	Arx	Camta1	Cacna1b
Bmp7	BC048507	Bcor	Asap1	Casc1	Cacna1c
Brd4	Bcan	Bdkrb2	Asic1	Cav2	Cacna2d2
Bri3	Bcar3	Best2	Atf7ip2	Cbll1	Cacng7
Bsn	Bcl11b	Bok	Atg7	Cbx6	Cadm1
Btbd16	Bcl3	Bop1	Atxn1	Ccdc88b	Camkk2
Btbd3	Bcl7c	Brd1	B3gnt7	Ccdc9b	Camta1
Btbd9	Bcor	Bsn	Bag3	Ccl1	Card6
C3	Begain	Cacna1h	Bahcc1	Ccnd1	Caskin2
CAAA01147332.1	Bicra	Cacna1i	Baiap2	Ccny	Cbln1
Cables1	Boll	Cacng7	Baiap3	Cdkn1c	Cbx6
Cacna1c	Bpgm	Cadm1	BC067074	Cdx4	Ccdc120
Cacna1e	Brinp3	Camta1	Bcar3	Celsr1	Ccdc177
Cacna1i	Bsn	Card10	Bcl7a	Cep128	Cdc20b
Cacna2d2	Btrc	Casc3	Bcor	Cep95	Cdh22
Cadm1	C1qtnf4	Catsperz	Begain	Cers1	Cdk16
Camta1	C330007P06Rik	Cbln1	Bglap2	Cfap46	Cdk8
Capn2	Cacna1a	Ccdc114	Bicc1	Cfap61	Celf4
Capn9	Cacna1c	Ccdc151	Bin2	Chn2	Celf5
Carmil1	Cacna1h	Ccdc22	Brd1	Chst14	Cenpu
Cass4	Cacng7	Ccdc60	Bri3	Cit	Chad
Casz1	Cacng8	Cd151	Brs3	Clcn4	Chd5
Cbarp	Cadm1	Cd55b	Bsn	Clip2	Chd9
Cbfa2t3	Camk4	Cdc20b	Btbd17	Clybl	Chrd
Ccdc110	Cap2	Cdkn1c	Btrc	Cmtm7	Chsy3
Ccdc177	Casz1	Cela1	C1qtnf5	Col11a1	Cib4
Ccdc180	Cbln1	Cerk	C2cd4c	Commd1	Clasrp
Ccdc22	Ccdc121	Chchd3	C3	Cpsf4l	Clpsl2
Ccdc60	Ccdc30	Chd9	Cacna1c	Cradd	Cmip
Ccdc81	Cd80	Chrd	Cacna1i	Crb2	Col26a1
Ccdc88b	Cdc42bpb	Chsy3	Camk2g	Crip3	Col4a2

Ccnd2	Cdh2	Clca3a1	Camk2n1	Crlf2	Col6a6
Ccr9	Cdh7	Clic4	Camkk1	Csmd1	Col9a1
Cd276	Cdk8	Clip1	Camta1	Ctdspl	Commd1
Cd93	Cebpa	Clip2	Capn8	Cuedc1	Comp
Cdc14a	Cela1	Clmn	Card11	Cux2	Coro2b
Cdca7l	Celf4	Clmp	Casz1	Cx3cl1	Cped1
Cdh22	Celf6	Clstn2	Cbarp	Cxxc5	Cpne7
Cdkn1b	Cfap53	Clybl	Ccdc177	Cyp26b1	Crb2
Celf4	Cfap97d2	Col26a1	Ccdc30	Cyp26c1	Crocc
Cep57l1	Chad	Cped1	Ccdc60	Cyp46a1	Crybg3
Cfap53	Chchd3	Cpne7	Ccnd2	Cyth1	Csf2
Cflar	Chd7	Crb2	Ccnd3	D13Ertd608e	Csf2ra
Chp2	Chd9	Creb5	Ccsap	Dbn1	Ctif
Chst13	Chn2	Crybb3	Cd276	Dclk2	Cux2
Chtf8	Chst13	Csf2	Cd93	Ddx3y	Cyld
Cic	Cilp2	Ctdspl	Cdc20b	Ddx5	Cyp24a1
Cilp2	Cit	Ctif	Cdc42bpb	Dgki	Dact3
Cit	Ckb	Ctnnd2	Cdc42bpg	Dlgap2	Dbn1
Cks1b	Clcn4	Cuedc1	Cdca7l	Dnm3	Dbp
Clcn4	Cldn15	Cul1	Cdh2	Dnmt3a	Dcaf8
Cldn14	Clybl	Cul9	Cdh22	Dtnb	Dcbld2
Clstn1	Cmklr1	Cux2	Cdh23	Dusp4	Dcdc2c
Clstn2	Cmss1	Cxxc5	Cdh4	Dync1li2	Dclk1
Clybl	Cnnm3	Cygb	Cdk19	Dysf	Deaf1
Cmip	Cntnap5a	Cyp26c1	Cdkn1b	E030030I06Rik	Dgkh
Cnbd2	Col18a1	Cyp46a1	Cdkn1c	Ebf1	Dixdc1
Col11a1	Col27a1	D430019H16Rik	Cers1	Ece1	Dlgap1
Col26a1	Col6a2	Dag1	Cfdp1	Ect2l	Dlgap2
Commd1	Commd1	Dcaf4	Chd9	Efna1	Dlx1
Coro1c	Coro2b	Dctd	Chil1	Ehbp1	Dmtn
Cpne2	Cpne2	Ddah1	Chn1	Elfn2	Dnase2a
Cpne5	Cpsf4l	Ddx3y	Chp2	Enc1	Dnm3
Cpxm1	Cradd	Dennd2a	Chrd	Endou	Dock9
Crb2	Crim1	Des	Chst8	Epha4	Dok7
Crb3	Crip3	Dhdds	Chtf8	Erf	Dst
Creb3l3	Crlf1	Dip2c	Cldn14	Errfi1	Dusp4
Creb5	Csf2	Disc1	Cldn4	Esrp1	Dusp6
Crebbp	Csgalnact1	Dlgap3	Clec3b	Esyt1	Ebf1
Crocc	Csk	Dll1	Clstn2	Etl4	Ebf2
Cry2	Ctif	Dnah6	Clybl	Etv6	Ece1
Ctif	Ctnna3	Dnajc18	Cmip	Evpl	Efhd2
Cuedc1	Cux2	Dnase1l2	Cmklr1	Exph5	Egln1
Cux2	Cxxc5	Dnm3	Cmss1	Ext2	Elfn2
Cyp26a1	Cycs	Dnmbp	Cntfr	Extl3	Epha4
Cyp26b1	Cyp46a1	Dot1l	Col11a1	Faim2	Ephb1
Cyp26c1	D13Ertd608e	Dpp9	Col26a1	Fam129b	Ephb4

D13Ertd608e	Dact1	Dusp6	Col2a1	Fam171a2	Eps15l1
D430019H16Rik	Dazl	Dync1h1	Commd1	Fam172a	Eras
D630039A03Rik	Ddn	Dysf	Coro1a	Fam19a4	Evx1
Dab1	Ddx3y	E4f1	Coro1c	Fam19a5	Exoc3l2
Dab2ip	Dennd1a	Ebf1	Coro7	Fam32a	Ext2
Dap	Dgkh	Edaradd	Cpne5	Farp1	Fads1
Dcun1d4	Dio1	Efhd2	Cradd	Fbxo10	Fam171a2
Ddn	Dip2a	Eif2s3y	Crb2	Fbxw7	Fam189b
Ddx3y	Dlc1	Eif4e3	Creb3l2	Fcgr3	Fam19a1
Dedd	Dlgap2	Elfn1	Crtc3	Fgf16	Fam20b
Dgkz	Dll1	Elk3	Cry2	Fgfr2	Fam222a
Dguok	Dll3	Enc1	Csf1r	Fig4	Fam32a
Diexf	Dnah10	Endou	Ctcfl	Fktn	Fam53b
Dip2b	Dnah6	Eng	Ctdspl	Fmnl1	Fam83h
Disc1	Dnajc12	Eps15l1	Cuedc1	Fos	Fbrsl1
Dkk2	Dnajc7	Erf	Cux2	Foxj3	Fbxl13
Dlg2	Dnm3	Erg28	Cxxc5	Foxn3	Fezf2
Dlgap1	Dnmt3a	Erich2	Cygb	Foxo6	Fgfr2
Dlgap2	Drc1	Esrrg	Cyp26b1	Frem2	Fhod1
Dlgap4	Dtx2	Etv6	Cyp26c1	Frmd4a	Fkbp7
Dmd	Dym	Extl1	Cyp4x1	Fry	Flt4
Dmpk	Dysf	Fam126a	Cyth4	Fryl	Fmod
Dmrt1	Ebf2	Fam160b2	D13Ertd608e	Fstl4	Fntb
Dmrt3	Ect2l	Fam171a2	D16Ertd472e	Gabbr2	Foxl1
Dmrta1	Edem1	Fam19a4	Ddr1	Gadl1	Foxn4
Dnah7b	Egln1	Fam20b	Dennd3	Galm	Foxo6
Dnaja3	Egr4	Fam83h	Dennd4a	Galnt2	Foxp4
Dnm3	Elmo1	Fat4	Des	Gas7	Frmd6
Dock9	Elp4	Fbf1	Dgkh	Gjb1	Frmd7
Dok5	Epas1	Fbrs	Dgkz	Glt1d1	Frmd8
Dot1l	Epb41l1	Fbxo24	Dhh	Gm10800	Frmpd1
Dst	Epcam	Fbxw19	Diexf	Gm1110	Fst
Dtnb	Epha4	Fbxw9	Dip2b	Gm13090	Fstl4
Dusp5	Epha6	Fezf2	Disc1	Gm14295	Fto
Dym	Ephb1	Fibp	Dlg2	Gm14399	Fut4
Dync1i1	Ephb2	Fig4	Dlgap2	Gm20388	Fut7
Dzip1	Errfi1	Fktn	Dlgap3	Gm20503	Fyn
E2f7	Esyt1	Foxo6	Dmpk	Gm20671	Fzd9
Eci2	Etv6	Fpr3	Dmrt3	Gm21863	Gabbr2
Efcab6	Evl	Frmd4a	Dmrta1	Gm28048	Gata2
Egr3	Evpl	Frmd4b	Dnaaf1	Gm3264	Gatm
Eif4e	Evx1	Frmd6	Dnaic1	Gm37240	Gcn1l1
Eif4g3	Faim2	Fry	Dnaja3	Gm43738	Gipc1
Elf2	Fam102a	Fto	Dnajc11	Gm8281	Git2
Elmsan1	Fam160a1	Fut4	Dnajc18	Gm9945	Gli1
Eml4	Fam167a	Fut7	Dnmt3a	Gng10	Gm10800

Emx2	Fam171a2	Gata5	Dock3	Gp1bb	Gm14295
Endou	Fam32a	Gcn1l1	Dock8	Gpd1	Gm20388
Enoph1	Fancd2os	Ggt5	Dpf3	Gprc5b	Gm20503
Enox1	Farp1	Glt1d1	Dpysl2	Grb10	Gm20517
Epcam	Fbxo30	Gm10800	Drd2	Grik4	Gm20708
Eps8l1	Fbxo46	Gm128	Dscam	Grin2b	Gm28048
Ern2	Fcrl5	Gm13090	Dst	Grip1	Gm37013
Errfi1	Fer1l6	Gm14295	Dtx2	Grk5	Gm37388
Esrrb	Fezf2	Gm14399	Dusp8	Grm2	Gm38393
Esrrg	Fgf12	Gm20388	Dyrk3	Grtp1	Gm42416
Esyt3	Fgfr1	Gm20481	Dysf	Gse1	Gm8108
Etl4	Fgfr2	Gm20503	E130114P18Rik	Gtsf1	Gm9857
Etnk2	Filip1l	Gm20517	E2f3	H2-Q6	Gnas
Etv4	Fktn	Gm20696	Ece1	Hcls1	Gng10
Etv5	Fosl1	Gm20708	Ect2l	Hcrtr2	Gp1bb
Ext2	Foxn3	Gm37013	Efna2	Heg1	Gpc1
Fam171a2	Foxn4	Gm37388	Egln1	Hic1	Gpc6
Fam32a	Foxo6	Gm42416	Egln2	Hip1	Gpr153
Fam53b	Frmd6	Gm45140	Egr3	Hivep2	Gpx8
Fancd2os	Frmd7	Gm45711	Eif4e	Hk3	Gramd1b
Fat1	Frrs1	Gm5475	Elf2	Hpca	Grik3
Fbf1	Fryl	Gm5617	Elfn2	Hs2st1	Grik4
Fbxo21	Frzb	Gnas	Elk3	Hs3st3b1	Grin1
Fbxo28	Fstl4	Gng10	Emx2	Hunk	Grm8
Fbxo46	Fut4	Gng7	En1	Hyal1	Gse1
Fer1l6	Fzd5	Gpc6	Enc1	Hydin	Gys1
Fes	Gas7	Gpd1	Epha4	Ica1	H2-Q6
Fezf2	Gata2	Gphn	Ephb1	Igf1r	Hcn1
Fgfr2	Gata5	Gpx8	Epm2aip1	Igf2r	Hcn3
Fignl2	Gata6	Grap2	Epn1	Igsf21	Henmt1
Fkbp10	Gfap	Grb10	Epn3	Il1r1	Hic1
Fmnl1	Ggn	Grin1	Erg28	Il3ra	Hivep3
Foxn3	Gja4	Grin3b	Errfi1	Impdh1	Hk1
Foxp4	Gli1	Grk1	Esco1	Inava	Hk3
Frmd5	Gli2	Gse1	Esrrb	Irf2	Hmg20b
Fxn	Glyctk	Gsx2	Esyt3	Irs1	Hmga2
Fzd5	Gm10295	Gtf2i	Etv6	Irs2	Hnf4g
Gabbr2	Gm10717	H13	Ext2	Irx3	Hpn
Gapvd1	Gm10718	Hcn2	Eya2	Islr2	Hs3st3a1
Gfpt2	Gm10775	Hdac4	Ezh1	Itpk1	Hs3st3b1
Gfra1	Gm10800	Hdac5	Fam102b	Itpr3	Hsd17b11
Ggnbp1	Gm14295	Hivep3	Fam110a	Jmy	Hsp90ab1
Ggta1	Gm14399	Hk2	Fam129a	Jph2	Hspa12a
Gjb2	Gm20388	Hk3	Fam169b	Jph3	Hspb1
Glp1r	Gm20503	Hs3st3a1	Fam171a2	Junb	Hspg2
Gltpd2	Gm28048	Hsd17b11	Fam184a	Kalrn	Iars2

Gm10775	Gm37240	Hsp90ab1	Fam213a	Kcns3	Igf2r
Gm14295	Gm45234	Hspa1a	Fam220a	Khsrp	Ildr1
Gm16867	Gm8281	Htr1d	Fam32a	Kiss1r	Immp2l
Gm17018	Gm9945	Hydin	Fam49a	Kit	Inpp5f
Gm20388	Gnas	Iffo2	Fam78b	Klf15	Inpp5j
Gm28048	Gng10	Igf2r	Fam81a	Klhl29	Insrr
Gm3264	Gpld1	Impact	Fam83h	Klk13	Iqsec3
Gm37013	Gpr37l1	Inpp5f	Fblim1	Lama1	Islr2
Gm37388	Gpsm1	Ipo13	Fbxl17	Lamb1	Itgb5
Gm38393	Grb10	Iqsec3	Fbxo21	Larp1	Itpka
Gm42416	Grid2	Irx1	Fbxo31	Lca5l	Itpkb
Gm42878	Grik3	Irx4	Fezf2	Lmf1	Jak3
Gm49322	Grik4	Isyna1	Fgf4	Lmntd1	Jph3
Gnas	Grin1	Itpka	Fgfr2	Lnx2	Jph4
Gng12	Grip1	Itpr3	Fhod1	Loxl1	Kbtbd11
Gramd1b	Grm2	Itsn1	Fignl2	Ltbp4	Kcnc4
Grasp	Gse1	Jak3	Fkrp	Lyzl4	Kcnh6
Grb10	Gstt2	Jph2	Flt1	Lzts3	Kcnip3
Grid1	Hcn1	Kat2a	Fndc7	Mad1l1	Kcnj14
Grik3	Hcn2	Kazn	Foxf1	Mael	Kif18b
Grip1	Hcrtr2	Kcnd3	Foxn4	Maml2	Kif26a
Gsc	Hes7	Kcng1	Foxo6	Maml3	Kif5c
Gse1	Hexim1	Kcnh6	Foxp4	Manf	Kifc1
Gsg1l	Hhipl1	Kcnip3	Frmd4b	Map3k4	Kirrel3
Gstm5	Hip1	Kcnj11	Frmd6	Map3k7cl	Kit
Haao	Hivep2	Kcnk4	Frrs1	Map7d1	Klf16
Has3	Hk3	Kcns1	Fry	Mapk15	Klhdc7b
Hdac5	Hlcs	Kctd3	Frzb	Mark1	Lhx6
Hhipl1	Hmg20b	Kdm4a	Fto	Marveld1	Lingo2
Hivep3	Hmga2	Kdm6b	Furin	Mast4	Lipo2
Hk1	Homer2	Kif13a	Galm	Mcoln2	Lipo3
Hlx	Hs3st1	Kif6	Galnt10	Mcoln3	Lmx1b
Hpca	Hs3st3a1	Kmt5a	Gata2	Mcu	Lnx2
Hrk	Hs6st3	Krt35	Gata6	Megf6	Lpcat3
Hs3st3b1	Hspg2	Ksr2	Gcnt4	Mex3a	Lrp8
Iffo2	Htr2a	Lca5l	Gfap	Mgll	Lrrc32
Ifrd2	Hunk	Lefty1	Gfpt2	Mn1	Lrrc69
Igdcc3	Hydin	Lekr1	Gfra2	Mrc2	Lrrc8d
Igf2r	Igf2r	Lhfp	Ggt1	Mtcl1	Lzts2
Il17ra	Il3ra	Lhx2	Ggt5	mt-Cytb	Lzts3
Ildr1	Impdh1	Lpp	Gjb1	Mtmr7	Mad1l1
Impact	Inava	Lrba	Gk	mt-Nd5	Man1c1
Insyn1	Inf2	Lrfn1	Glg1	Myl4	Map4k1
Irs2	Iqcg	Lrg1	Gli2	Myo15	Map7
Irx5	Itga9	Lrr1	Gli3	Myo1e	Mapk11
Itga9	Itpk1	Lrrc29	Gm11032	Naa80	Mapk15

Itm2c	Itpr3	Lrrc32	Gm14295	Nacc2	Matn4
Itpk1	Jcad	Lrrc43	Gm20388	Nadk2	Mcph1
Itpkb	Jph2	Lrrc69	Gm20503	Nav2	Mdga1
Itpr1	Jph3	Lsm4	Gm26938	Ncoa3	Me3
Jakmip1	Kalrn	Lzts2	Gm27021	Ndrg4	Med26
Jarid2	Kbtbd11	Mamstr	Gm28048	Ndst1	Megf6
Jcad	Kcnip1	Man1c1	Gm28308	Nectin4	Mfap3l
Kalrn	Kcnj4	Map9	Gm37013	Nek5	Mfsd12
Kazn	Kcnk2	Mark1	Gm37240	Nfix	Mgat5b
Kbtbd11	Kcnq4	Marveld2	Gm37388	Nipal3	Mical3
Kcna5	Kctd15	Mb	Gm38393	Nipbl	Mmp2
Kcnc1	Kirrel3	Mcts2	Gm42416	Nkain1	Mmp24
Kcnk12	Klhl29	Mcu	Gnal	Nkx2-2	Mn1
Kdm2b	Krt80	Mdga1	Gnas	Nmral1	Mpl
Kif13a	Krtap17-1	Mecom	Gnaz	Nol4l	Mrps27
Kif1a	L3mbtl1	Med13l	Gne	Notch1	Msl3
Klf14	Large2	Meis1	Gng10	Npcd	mt-Atp6
Kpna1	Lca5l	Meis2	Gpalpp1	Nphs1	mt-Atp8
Krtap17-1	Lgi2	Mettl7a3	Gpd1	Nr2f1	mt-Co1
L3mbtl3	Lhx6	Mex3a	Gpr27	Nr4a2	mt-Co2
Lamb2	Lin28a	Mfsd12	Gpsm1	Nr5a2	Mtf1
Lats2	Lingo1	Mmp11	Gpt	Nrap	mt-Nd1
Lbh	Lmf1	Mn1	Gpx8	Nrg2	mt-Nd2
Ldlrad4	Lpp	Morn1	Grb10	Nrg3	mt-Nd6
Lef1	Lrp5	Mpl	Grb7	Nt5c2	Mtss1l
Lgr5	Lrp8	Mpo	Grin1	Ntn4	Myh3
Lhx4	Lrrc1	Mpp2	Grin3b	Nup188	Mylk
Lhx9	Lrrtm3	Mrm2	Grk5	Nxph1	Myo10
Lin7a	Ltbp3	mt-Atp6	Gse1	Otog	Myrfl
Lingo1	Ltbp4	mt-Atp8	H13	Otop2	Mzf1
Lmo4	Macf1	mt-Co1	Haao	Otx2	Nacc2
Lmtk2	Magi1	mt-Co2	Hdac5	Pabpc4	Nap1l3
Lrfn2	Map2k7	mt-Cytb	Heca	Papolb	Nbeal2
Lrp5	Map3k14	Mtg1	Heg1	Parp1	Nbl1
Lrp8	Map7d1	Mtmr7	Hhipl1	Pax8	Ncaph
Lrrc18	Mapk15	mt-Nd1	Hic1	Pde4d	Necab2
Lrrc4b	Mapk1ip1l	mt-Nd2	Hlcs	Peak1	Nectin4
Lrrc8e	Mapkap1	mt-Nd6	Hmg20b	Peg10	Neurl1a
Ltbp2	Mapt	Mtnr1a	Hmgcl	Peli2	Nfix
Ltbp4	Mcc	Mtss1	Hmgcr	Phactr1	Nhsl1
Macrod1	Me3	Mtss1l	Hnf1a	Phldb1	Nkx2-3
Mafg	Mecom	Mylk	Hnrnpul1	Pih1d1	Nlgn2
Magi1	Meis1	Myo18a	Hoxa3	Pik3r1	Nos3
Map3k10	Meis2	Myo18b	Hoxa5	Pip5k1c	Notch3
Map7	Mesp2	Myo3b	Hs3st3a1	Pisd	Npas3
Mapk10	Mgat5	Myrip	Hs6st2	Pitpnm2	Npcd

Mapkapk5	Mgat5b	Mzf1	Hsf5	Plekhg3	Npffr1
Marveld2	Mgll	Nacc2	Hunk	Plekhh2	Nr2f1
Marveld3	Mmp11	Nap1l3	Iffo2	Plekhh3	Nr4a2
Mdk	Mmp9	Napsa	Igf2	Plpp7	Nradd
Me3	Mn1	Nav2	Igf2r	Plxna4	Nrp2
Mecom	Morn1	Nbn	Il6ra	Plxnd1	Nrxn1
Meioc	Mospd1	Nectin4	Ildr2	Pmepa1	Ntrk2
Meltf	Mpo	Neurl3	Impact	Pnmal2	Nxph4
Mettl14	Mpp2	Nfix	Inava	Polr1d	Obscn
Mex3a	Mrap	Nhej1	Inf2	Pop5	Obsl1
Mfhas1	mt-Atp6	Nkpd1	Inhba	Pou6f2	Olfm2
Mfsd12	mt-Atp8	Nkx2-3	Inpp5a	Ppfia4	Onecut2
Mgmt	Mtcl1	Nod1	Ipcef1	Ppm1h	Otog
Mical3	mt-Co1	Nol4l	Ipo5	Ppp1r26	Otop2
Mier2	mt-Co2	Nos3	Irs2	Ppp2r3d	Oxsr1
Mkrn3	mt-Cytb	Nr2f1	Irx1	Ppp2r5b	Panx1
Mlxipl	mt-Nd1	Nsf	Irx3	Ppp3ca	Papln
Mn1	mt-Nd2	Nt5dc3	Itga9	Ppp4r1	Parp12
Morn1	mt-Nd4	Ntrk2	Itgb3	Prag1	Pars2
Mpo	mt-Nd5	Nudt1	Itgb4	Prex1	Pbx1
Mrc2	Mtss1	Numa1	Itgb5	Prkag2	Pcdh1
Msantd3	Mtx1	Nxph4	Itpkb	Prkcb	Pcdhga1
Mtf1	Muc1	Obscn	Jak3	Prn	Pcdhga10
Mthfsl	Mxi1	Obsl1	Jakmip1	Prnp	Pcdhga11
Mtus2	Myf5	Ocstamp	Jph3	Prox1	Pcdhga12
Mxra7	Myh9	Olfr279	Jup	Prrt1	Pcdhga2
Mylk	Myo18a	Onecut3	Kalrn	Prss44	Pcdhga3
Myo18a	Myo1d	Otud7a	Kank1	Prune2	Pcdhga4
Myo1e	Myo1h	Oxsr1	Kat14	Psrc1	Pcdhga5
Mzf1	Myt1l	Oxt	Katna1	Ptp4a2	Pcdhga6
Ncor2	Nav2	P4htm	Kcna10	Ptprn	Pcdhga7
Nefm	Ncam1	Pacrg	Kcng2	Ptpro	Pcdhga8
Nfatc1	Nckap5l	Pak4	Kcnh2	Ptpru	Pcdhga9
Nfib	Ncor2	Panx2	Kcnh7	Pttg1ip	Pcdhgb1
Nfix	Nedd4l	Pax6	Kcnip1	Ptx3	Pcdhgb2
Nfkbil1	Neurl3	Pcdha1	Kcnn4	Pxn	Pcdhgb4
Nhsl1	Nfatc2	Pcdha11	Kcnq1	Rab19	Pcdhgb5
Nin	Nfic	Pcdha12	Kcp	Rabac1	Pcdhgb6
Nkpd1	Nfix	Pcdha2	Kdm4b	Radil	Pcdhgb7
Nkx2-9	Nhej1	Pcdha3	Kdr	Rai1	Pcdhgb8
Nlrp5	Nhsl1	Pcdha4	Kifc3	Rasa3	Pcdhgc3
Nnat	Nkiras2	Pcdha5	Kirrel3	Rbfox3	Pcdhgc4
Noto	Nkx2-2	Pcdha6	Klf6	Rbm15b	Pcdhgc5
Nova2	Nmral1	Pcdha7	Klf7	Rbm33	Pcolce
Npdc1	Notch4	Pcdha8	Kntc1	Rbpms	Pde11a
Npepl1	Noto	Pcdha9	Krtap17-1	Rcan1	Pde4d

Nphs1	Nova2	Pcdhga1	Lca5l	Rfx4	Pde4dip
Npr3	Npdc1	Pcdhga2	Lef1	Rgl3	Pdx1
Nr1d1	Nptx2	Pcdhga3	Lhx4	Rimklb	Peak1
Nr2f1	Nr2f1	Pcdhga4	Lhx9	Rlbp1	Pear1
Nr2f2	Nrp2	Pcdhga5	Lima1	Rnf144b	Pebp4
Nrp2	Nrtn	Pcdhga6	Lin7a	Rnf157	Peli2
Nrxn2	Nrxn1	Pcdhga7	Lmf1	Rnf217	Pepd
Nxn	Nrxn2	Pcdhga8	Lmna	Rnf39	Phactr3
Nxph1	Ntn4	Pcdhgb1	Lmntd1	Rora	Phospho1
Oacyl	Nup62cl	Pcdhgb2	Lmtk3	Rtl1	Pik3cd
Olfm2	Nxn	Pcdhgb4	Loxhd1	Runx1	Pip5k1c
Olig1	Nxph1	Pcgf3	Lpar3	Runx3	Pitpnc1
Onecut2	Nxph4	Pcmtd1	Lpcat3	Rusc1	Plagl1
Ooep	Nyx	Pcsk6	Lrba	Rusc2	Plcb4
Osbpl1a	Obscn	Pde10a	Lrfn1	Ryr1	Plch2
Otx2	Odf2	Pde4d	Lrp1	Sarm1	Plekhh3
Oxr1	Onecut3	Pde4dip	Lrp6	Sash1	Plekho1
P3h4	Oprd1	Pde7b	Lrp8	Scarf2	Pmepa1
Pacrg	Ostm1	Pfkfb4	Lrrc32	Scrt1	Pnpt1
Pacsin3	Otop2	Pfn3	Lrrc4	Scrt2	Pop5
Panx2	Otx2	Phactr4	Lrrc8d	Scx	Ppp2r3a
Pax6	Pacrg	Phc2	Ltbp4	Sdccag8	Ppp3ca
Pcdh10	Pakap	Phospho1	Luzp2	Sdk1	Ppp3cc
Pcdhga1	Palm2	Pik3cd	Mad1l1	Sdk2	Ppt2
Pcdhga10	Pax5	Pitpnc1	Maml2	Sgcd	Prag1
Pcdhga11	Pax6	Plekhg3	Maml3	Sgce	Prdm16
Pcdhga12	Peg10	Plpp1	Mamstr	Sh3gl3	Prkca
Pcdhga2	Peg12	Plpp3	Man1c1	Sh3pxd2a	Prorsd1
Pcdhga3	Phactr1	Plpp7	Man2a1	Sh3pxd2b	Prox1
Pcdhga4	Phf21b	Pnpt1	Map3k13	Shank2	Prr23a2
Pcdhga5	Phldb1	Pop5	Map3k4	Shroom4	Prr36
Pcdhga6	Phospho1	Ppfia4	Map7d1	Six1	Prrt1
Pcdhga7	Pigk	Ppp2r1b	Mapk10	Six3	Psap
Pcdhga8	Pih1h3b	Ppp3cc	Mapk14	Slain2	Ptchd3
Pcdhga9	Pik3c2b	Pptc7	Mapk15	Slc10a7	Ptchd4
Pcdhgb1	Pik3cd	Prcd	Mapt	Slc11a2	Pter
Pcdhgb2	Pik3r1	Prdm8	Marveld3	Slc16a3	Ptger4
Pcdhgb4	Pla2r1	Prex1	Mast1	Slc22a3	Ptpn11
Pcdhgb5	Plaur	Prickle1	Mb	Slc2a12	R3hdm2
Pcdhgb6	Plch2	Prickle2	Mbnl2	Slc35a1	Rab31
Pcdhgb7	Pld5	Prkar1b	Mcts2	Slc37a1	Rab40c
Pcdhgb8	Plekhg3	Prkce	Mcu	Slc38a10	Rad23b
Pcdhgc3	Plin1	Prr12	Mdga1	Slc38a4	Ralgps1
Pcdhgc4	Pllp	Prr5l	Mdk	Slc39a10	Rap1gap
Pcdhgc5	Plpp1	Prss44	Med18	Slc39a11	Rasa3
Pcsk6	Plpp7	Prune2	Med31	Slc4a4	Rassf3

Pdzd7	Plxna1	Psmc3ip	Mef2d	Slc4a8	Rbfox3
Pebp4	Pou6f2	Ptch1	Mfrp	Slc7a3	Rbm19
Pepd	Ppard	Pter	Mgat5b	Slit3	Rbms3
Pgrmc1	Ppm1h	Ptpn11	Mindy4	Smad1	Rbpjl
Phactr1	Ppp1r13l	Ptprf	Mlh1	Smad6	Rex1bd
Phyhip	Ppp1r3g	Ptprg	Mllt1	Smpx	Rftn1
Pigf	Ppp2r2c	Ptprn2	Mn1	Smurf1	Rgs22
Pik3c2b	Ppp3cc	Ptprr	Mrap	Sorbs3	Rgs3
Pipox	Prdm16	Ptprt	Msi2	Sowaha	Rimklb
Pitpnm3	Prex1	Pyy	Msrb1	Sox13	Rnf135
Plagl1	Prkcb	Rab43	Mtcl1	Sox6	Rnf150
Pld6	Prmt8	Rai1	Mthfd1	Spata31d1d	Rnf220
Plekha6	Prorsd1	Ramp1	Mthfsl	Speg	Rnf26
Plekha7	Proser2	Rap1gap2	mt-Nd1	Spock1	Rspo1
Plekhg4	Prox1	Rasal3	mt-Nd2	Sptbn1	Rtbdn
Plekhh3	Prrt4	Rasip1	Mtss1	Spx	Runx2
Pnmal2	Prss36	Rbfox3	Myl4	Src	Ruvbl2
Pnpt1	Ptges	Rbm7	Myo19	Srgap1	Scimp
Podn	Ptprg	Rhobtb1	Myo1e	Srrm4	Sdk1
Pole4	Ptprr	Rilpl1	Mypn	Srrt	Selenoi
Pou2f1	Pttg1ip	Rims1	Mzf1	St6galnac3	Sema4f
Pou3f1	Ralgds	Rin1	Nacc2	Stpg1	Serinc3
Pou6f1	Ranbp3l	Rlf	Nav1	Strap	Set
Ppargc1b	Rasal3	Rnf121	Nav2	Supt5	Sh2b2
Ppfia4	Rbmxl2	Rnf138rt1	Nbeal2	Synj2	Sh2b3
Ppp1r13l	Rex1bd	Rnf144b	Ncor2	Syt12	Shank1
Prag1	Rgs22	Rnf150	Ndufc1	Szt2	Shank2
Prdm11	Rhoq	Rnf26	Necab2	Tacc1	Shank3
Prdm16	Rnf121	Rtbdn	Neurl3	Tacc2	Shh
Prkca	Rtn1	Rxfp2	Nfatc2	Tanc1	Shisal1
Prkce	Rtn4rl2	Rxra	Nfib	Tbx18	Sik1
Prkd2	Rusc2	Scn1b	Nfil3	Tbxa2r	Sipa1l1
Prr12	Rxrg	Scyl3	Nfix	Tcp11	Sipa1l3
Prr29	Ryr1	Selenoi	Nhsl1	Thegl	Six1
Prrc1	S100a1	Sema4f	Nim1k	Tinagl1	Six3
Prss50	S100a13	Setbp1	Nkain2	Tjp2	Ski
Ptges	Scrt1	Sft2d1	Nkx2-2	Tmc6	Slc13a3
Ptp4a2	Sdccag8	Sh2d3c	Nlrp4e	Tmc8	Slc22a14
Ptprn2	Sdk2	Sh3rf1	Nmral1	Tmem132b	Slc23a2
Ptprr	Sema6c	Shank3	Nos2	Tmem132c	Slc25a37
Pxn	Serinc5	Shisa3	Notch2	Tmem242	Slc25a48
Rab31	Sft2d1	Shroom1	Notch3	Tmem268	Slc2a12
Rai1	Sgcd	Sik2	Noto	Tnfrsf1a	Slc38a10
Ralgapa2	Sgce	Sik3	Npepl1	Tnrc18	Slc39a11
Rap1gap2	Sh2b2	Six1	Nr4a2	Tns2	Slc44a5
Rarg	Sh3gl3	Skida1	Nradd	Tob2	Slc7a1

Rarres1	Sh3pxd2a	Slc10a7	Nrg1	Tox2	Slc9a5
Rbfox3	Sh3pxd2b	Slc11a2	Nrxn1	Tpcn1	Smarcal1
Rbmxl2	Shank3	Slc12a5	Nrxn2	Traf3	Smoc1
Reln	Shisa3	Slc13a2	Nrxn3	Tram2	Smpd5
Rftn1	Shisal1	Slc13a4	Ntng2	Trim26	Snrpn
Rfx4	Sirt1	Slc16a12	Nufip1	Tshz2	Snurf
Rims2	Sis	Slc22a14	Numa1	Tspan14	Socs5
Rmdn1	Six1	Slc23a2	Nup210l	Ttc21b	Sod3
Rnls	Slc12a4	Slc25a37	Nxph4	Ttll11	Sox13
Rora	Slc12a8	Slc26a10	Olfm2	Ube2o	Sox18
Rpl5	Slc14a2	Slc2a9	Onecut2	Uchl1	Sp9
Rps9	Slc16a14	Slc37a1	Opn4	Ucp3	Spaca1
Rtn2	Slc20a2	Slc4a1	Oprd1	Uhrf1bp1	Spatc1
Runx2	Slc25a13	Slc7a14	Oprm1	Ush1g	Speg
Rusc2	Slc36a1	Slc7a3	Ost4	Usp29	Spock1
Rxra	Slc37a1	Slc9a1	Otoa	Utf1	Spry1
Ryr1	Slc41a3	Slit3	Otud7a	Vash2	Spsb1
S1pr5	Slc43a3	Smad3	Oxt	Vasp	Sptb
Scg2	Slc7a3	Smim13	Pacsin3	Veph1	Srgap1
Scn8a	Slc8a1	Smtn	Pak4	Vkorc1l1	Ssbp3
Scrt2	Slco3a1	Sobp	Pak6	Vps18	St3gal4
Sdk1	Smad7	Socs5	Palld	Vstm2l	St8sia1
Sdsl	Smarcc1	Sorcs2	Panx1	Wbp1l	Stat1
Sec14l5	Smg6	Sorl1	Panx2	Wdr95	Stn1
Sec16b	Smoc1	Sp9	Pard6g	Wnt3	Sulf2
Selenom	Smyd4	Spats2l	Parva	Wnt5b	Supt3
Sema4d	Snx4	Speg	Parvb	Wnt7b	Susd4
Sesn1	Sorcs2	Spock1	Pawr	Zbtb16	Sycp2l
Sfrp4	Sowaha	Spsb1	Pax2	Zbtb20	Synrg
Sgcd	Sox6	Sptbn4	Pax6	Zdhhc18	Tac1
Sgsm1	Sp9	Srrm3	Pcbp3	Zfp316	Tamm41
Sh2d3c	Spag16	Ssbp4	Pcdh9	Zfp385c	Tbc1d16
Sh3gl3	Speg	St6galnac3	Pcdhga1	Zfp423	Tbkbp1
Sh3pxd2a	Spidr	Suds3	Pcdhga10	Zfp444	Tbx4
Shank3	Spock1	Sugp2	Pcdhga11	Zfp503	Tcf4
Shc1	Spred3	Sulf2	Pcdhga12	Zfp536	Terb1
Shh	Sptb	Sult2b1	Pcdhga2	Zfp560	Tfr2
Shroom3	Srrm4	Syce3	Pcdhga3	Zfp697	Thpo
Six1	Ssbp3	T2	Pcdhga4	Zfp703	Tirap
Six5	St3gal3	Tacc1	Pcdhga5	Zfp777	Tln2
Skap1	St6galnac3	Taf7l	Pcdhga6	Zfp853	Tlx1
Slc12a4	St6galnac5	Tbc1d16	Pcdhga7	Zfpm1	Tm9sf2
Slc19a2	Stk39	Tbkbp1	Pcdhga8	Zrsr1	Tmcc1
Slc22a3	Stox2	Tbx15	Pcdhga9		Tmem119
Slc25a13	Stx1a	Tcf24	Pcdhgb1		Tmem125
Slc25a21	Suds3	Tcte2	Pcdhgb2		Tmem150c

Slc25a23	Supt5	Tdrd7	Pcdhgb4	Tmem151b
Slc30a7	Synj2	Tead1	Pcdhgb5	Tmem164
Slc32a1	Synpo	Tecr	Pcdhgb6	Tmem240
Slc35e4	Syt6	Tenm3	Pcdhgb7	Tmem267
Slc39a11	Tbc1d1	Tenm4	Pcdhgb8	Tmem28
Slc44a5	Tbk1	Tfr2	Pcdhgc3	Tmem59l
Slc6a20a	Tbx4	Tgfb1	Pcnx2	Tmem80
Slit3	Tbxa2r	Thpo	Pde10a	Tnfaip2
Smad1	Tbxas1	Tiam1	Pde4c	Tnik
Smad6	Tcf4	Ticam2	Pdgfa	Tnk1
Smad7	Tcf7l2	Tjp2	Pdgfd	Tns2
Smurf1	Tenm2	Tlx1	Pdgfra	Togaram2
Snd1	Tex2	Tmcc3	Pdk1	Trappc10
Snrnp70	Tfap2c	Tmed7	Pdyn	Trappc9
Snrpn	Tfr2	Tmem125	Pdzd2	Trp53i11
Sntg2	Tgfb1	Tmem132b	Peak1	Tsc22d4
Snurf	Tgfbr3l	Tmem150c	Pebp4	Tspan7
Snx18	Thegl	Tmem151b	Peg3	Ttc17
Sobp	Thtpa	Tmem164	Pet117	Ttc33
Socs2	Tiam1	Tmem165	Pf4	Ttc7
Socs5	Tln2	Tmem208	Phactr3	Ube3b
Sorcs2	Tmem132c	Tmem241	Phf21b	Ubn2
Sox15	Tmem145	Tmem28	Phlda3	Umodl1
Sox21	Tmem163	Tmem63a	Phldb1	Unc5a
Spata13	Tmem200c	Tmem88	Phospho1	Ush1g
Speg	Tmem267	Tnik	Piezo2	Usp2
Spsb4	Tmem30c	Tnrc18	Pigk	Usp35
Srcin1	Tnik	Tor2a	Pih1d1	Usp49
Srgap1	Tnks1bp1	Tor4a	Pipox	Vstm2l
Srgap3	Tnrc6b	Trappc9	Pkig	Wdr27
Srrm4	Tpcn1	Trim8	Plch2	Wdr6
Ssbp3	Tpgs1	Trp53i11	Plek2	Wipf1
Stat1	Tpm1	Trub1	Plekha2	Wnt10a
Stum	Traf3	Tsc22d1	Plekhg4	Wnt7b
Sugp2	Traip	Tshz2	Plin1	Wnt8a
Supt5	Trak1	Tspoap1	Plp2	Xkr7
Sycp2l	Tram2	Ttc16	Plpp3	Zbtb2
Syt12	Trim17	Ttc28	Plpp4	Zdhhc18
Syt6	Trp73	Ttc41	Plpp7	Zfp316
Tanc1	Trub1	Ttll8	Pnpla1	Zfp341
Tbkbp1	Tsc22d1	Tubb5	Pnpt1	Zfp365
Tbx1	Tshz2	Tubgcp6	Porcn	Zfp46
Tbx2	Tspan15	Ubn2	Pou2f2	Zfp521
Tbx3	Tspan18	Ubr2	Ppard	Zfp560
Tcf4	Tspoap1	Ubxn11	Ppfia4	Zfp608
Tcf7l2	Ttc23	Uroc1	Ppp1r37	Zfp652

Terf1
Tet3
Tfr2
Thbd
Tjp2
Tle3
Tmcc3
Tmem108
Tmem125
Tmem150c
Tmem181a
Tmem242
Tmem268
Tmem54
Tmem59l
Tmem63a
Tnfaip2
Tnfrsf1a
Tnrc18
Tnrc6b
Tns1
Tnxb
Togaram2
Tomm40
Tpcn1
Traf3
Treh
Trim2
Trove2
Tspan17
Ttbk1
Ttc28
Ttc34
Ttc7
Ttll11
Ttn
Tuba8
Tubgcp6
Uck2
Ulk4
Unc5a
Unc5cl
Upf1
Usp15
Utf1
Utp4

Ttc4
Ttll6
Ubac1
Ube2a
Uchl1
Unc5b
Ush1g
Usp15
Usp35
Usp6nl
Vps37b
Vrk3
Vwa8
Wbp1l
Wipf3
Wnk4
Wnt3
Wnt3a
Wwc1
Wwox
Xkr7
Ypel2
Ythdc2
Zadh2
Zfand3
Zfhx2
Zfp316
Zfp36
Zfp444
Zfp473
Zfp503
Zfp579
Zfp652
Zfp777
Zfp853
Zfpm1
Zmat4
Zmiz1
Zmynd8
Zrsr1
Zswim9

Usp2
Usp35
Usp46
Usp49
Usp50
Vav2
Vdr
Vgll4
Vps35l
Vstm2l
Vwf
Wdr66
Wdr86
Wnt7a
Wnt7b
Wnt8a
Wscd1
Wwc1
Xndc1
Xntrpc
Ypel2
Zbtb20
Zfp316
Zfp365
Zfp503
Zfp536
Zfp652
Zfpm1
Zmat4
Zmynd8
Zp3r

Ppp2r1b
Ppp2r2c
Ppp2r5c
Ppp3cc
Prag1
Prcd
Prdm15
Prkca
Prkd2
Prkn
Prr12
Prrc2b
Prrt4
Prss16
Prss50
Pstpip2
Ptch1
Ptger1
Ptgir
Ptgis
Ptp4a2
Ptpn1
Ptprn2
Pttg1ip
Pxk
Pyroxd2
Qrfp
Qtrt2
R3hdm2
Rab11fip4
Rab12
Rab19
Rab31
Rai14
Rara
Rarg
Rarres1
Rasa3
Rasal1
Rasip1
Rassf1
Rbbp7
Rbfox3
Rbm19
Rex1bd
Rgs3

Zfp697
Zfp703
Zfp710
Zfp777
Zfpm1
Zmiz1
Znrf1
Zrsr1
Zscan20

Vstm2b
Vstm2l
Vwf
Wap
Wbp1l
Wdfy4
Wdr66
Wdr86
Wipf1
Wipf3
Wwc1
Xdh
Xkr6
Ypel1
Ypel4
Zbtb7c
Zfand3
Zfhx3
Zfp275
Zfp385a
Zfp423
Zfp444
Zfp462
Zfp532
Zfp536
Zic1
Zic4
Zrsr1
Rgs9
Rhobtb1
Ripor3
Rnf122
Rnf125
Rnf135
Rnf220
Rnf26
Rnf39
Rnmt
Rph3al
Rpl5
Rsph14
Runx2
Rxra
Sall1
Sbk1
Scarf2
Scd2
Scube2
Sdc4
Sdk1
Sec11c
Sec14l1
Selenoi
Selenom
Selp
Sema4f
Sema5a
Sema6c
Sergef
Serinc2
Sez6
Sgk1
Sgpl1
Sgpp2
Sh2b2
Sh3gl3
Sh3pxd2a
Sh3rf2
Shank3
Shb
Shh
Shisal1
Shroom1
Sipa1l1

Sirt4
Six5
Skap1
Ski
Slc19a2
Slc26a2
Slc35e4
Slc44a5
Slc4a1
Slc8a2
Slc9a5
Slit3
Smad1
Smad3
Smg6
Smtn
Smurf2
Smyd4
Snd1
Snrpn
Sntg2
Snurf
Snx18
Snx33
Sobp
Sorbs3
Sorcs2
Sox21
Sox6
Sox7
Spef1
Speg
Srcin1
Srgap1
Srrm3
Ssbp3
St6galnac2
St6galnac5
Stac3
Steap3
Stk24
Sugp2
Supt5
Syk
Syngap1
Syngr3

Taok3
Tbc1d30
Tbk1
Tbx3
Tbx4
Tbxa2r
Tcf15
Tcf4
Tcf7
Tcf7l1
Tcf7l2
Tecpr1
Tenm3
Tenm4
Tet1
Tex2
Thbd
Thegl
Thpo
Thrap3
Tiam1
Tiam2
Tle2
Tm7sf3
Tm9sf3
Tmem119
Tmem125
Tmem132c
Tmem150c
Tmem159
Tmem181a
Tmem184a
Tmem191c
Tmem201
Tmem267
Tmem51
Tnfaip1
Tnfrsf1a
Tnks
Tns2
Tox2
Tpst2
Trak1
Trappc9
Tsc22d4
Tshz1

Tshz2
Tspoap1
Ttbk1
Ttc28
Ttc34
Ttc7
U2af2
Ubash3b
Ube3b
Unc5b
Unc79
Usf2
Ush2a
Usp29
Utp4
Vac14
Vasn
Vav2
Vps8
Vti1a
Vwa5b1
Vwa5b2
Vwf
Wasf2
Wdr66
Wipf1
Wipf3
Wnt10a
Wnt7a
Wnt8a
Wscd1
Wscd2
Wwox
Xkr6
Xkr7
Ylpm1
Ypel4
Zadh2
Zbtb20
Zbtb7c
Zdhhc18
Zfp384
Zfp407
Zfp423
Zfp462
Zfp503

Zfp536
Zfp608
Zfp652
Zfp710
Zfp853
Zfpm1
Zfyve21
Zic1
Zic4
Zkscan17
Zmynd8
Zrsr1

Table S2. Full list of differentially methylated genes across the three different timepoints under AL CCH with the breakdown of list of genes hyper and hypo-methylated respectively.

AL BCAS7D	AL BCAS15D	AL BCAS30D	AL BCAS7D	AL BCAS15D	AL BCAS30D
Abcc4	Abi2	Abcc4	Abat	4931414P19Rik	Abca16
Actn1	Abi3	Abi3	Abcc4	Abcc4	Abcb9
Adora2a	Acsf2	Acot1	Abl1	Actg1	Abcc4
Ahdc1	Acss1	Acta1	Ablim1	Acvr2b	Acer2
Akap6	Actn1	Actn1	Actg1	Ada	Acin1
Alox5ap	Adam32	Acvr2b	Acvr1	Adamts20	Actg1
Anp32a	Adamts1	Adcy5	Adam12	Adamtsl3	Actn1
Ap3b1	Adra1b	Adgrb2	Adam33	Adcy6	Adam11
Apoh	Ajap1	Adgrg6	Adamts17	Adra1d	Aff3
Atf6b	Akap9	Afmid	Adap2	Aen	Agbl4
B230104I21Rik	Amn	Agap1	Adcy4	AI464131	Akr1b8
Bach2	Ank3	Agap2	Adgrb2	Akap6	Alox5ap
Bahd1	Ankrd34b	Ahdc1	Adora1	Akap7	Ankrd35
Barhl1	Ano2	AI464131	Adra1b	Akp3	Ap3b2
Barx2	Ap1m2	Ak5	Adra2c	Aldh8a1	Arhgap8
Bcar3	Arhgef10	Akt1	Aldh18a1	Als2	Arhgef3
Bckdha	Arrdc5	Aldh1l1	Alk	Amotl2	Arid2
Bcl11b	Aspg	Alk	Alox5ap	Anks1b	Atp11b
Bcor	Atg7	Amn	Ank2	Ano6	Atp1a3
Blcap	Atp4a	Ank	Ank3	Ap1b1	Atp5o
Bmp7	Atp6v0a1	Aqp1	Anks1	Apba2	B3gat2
Btbd16	Atp6v0a2	Arhgap21	Anks6	Arhgap21	Bag3
Btbd9	Atxn1	Arhgef10	Ano4	Arhgap26	Banp
C3	Axin2	Arhgef28	Apbb2	Arhgef10	Baz2b
CAAA01147332.1	B3gat2	Arl14	Arhgap15	Arhgef28	BC048507
Cacna1e	B3gnt3	Arl14epl	Arhgap22	Arl15	Bcan
Casz1	B3gnt8	Arrdc5	Arhgef10	Atn1	Bcl11b
Cbarp	B4galt1	Arsg	Arhgef2	Atp9a	Bdkrb2
Ccdc22	Basp1	Asic1	Arhgef28	B4galt2	Best2
Ccdc60	Bcl11b	Atg7	Arx	Banp	Bok
Ccdc81	Begain	Atp5o	Asap1	BC017158	Bop1
Ccdc88b	Boll	Atxn1	Atp6v1g2	BC048507	Bsn
Cdkn1b	Bsn	B3gnt3	Azi2	Bcan	Cacna1h
Celf4	Btrc	Bcar3	B3gat2	Bcar3	Camta1
Cep57l1	C330007P06Rik	Bcor	Bahcc1	Bcl3	Catsperz
Chst13	Cacna1a	Bop1	Basp1	Bcl7c	Cbln1
Cilp2	Cacna1h	Brd1	Bcam	Bcor	Ccdc114
Clstn1	Camk4	Bsn	Begain	Bicra	Ccdc151
Clybl	Casz1	Cacna1i	Bicc1	Bpgm	Cd55b
Col26a1	Ccdc121	Cacng7	Bicra	Brinp3	Cdc20b
Commd1	Cd80	Cadm1	Brd4	C1qtnf4	Cdkn1c
Coro1c	Cdk8	Card10	Bri3	Cacna1c	Cela1

Cpne2	Cela1	Casc3	Bsn	Cacng7	Cerk
Cpne5	Celf4	Ccdc22	Btbd3	Cacng8	Chsy3
Cpxm1	Celf6	Ccdc60	Cables1	Cadm1	Clca3a1
Crocc	Cfap53	Cd151	Cacna1c	Cap2	Clstn2
Ctif	Cfap97d2	Cdkn1c	Cacna1i	Cbln1	Clybl
Cux2	Chad	Chchd3	Cacna2d2	Ccdc30	Cpne7
Cyp26b1	Chd7	Chd9	Cadm1	Cdc42bpb	Crybb3
D630039A03Rik	Chst13	Chrd	Camta1	Cdh2	Ctnnd2
Dap	Cilp2	Clic4	Capn2	Cdh7	Cxxc5
Ddx3y	Ckb	Clip1	Capn9	Cebpa	Dctd
Dguok	Cldn15	Clip2	Carmil1	Celf4	Dlgap3
Diexf	Cntnap5a	Clmn	Cass4	Chchd3	Dll1
Dkk2	Commd1	Clmp	Cbfa2t3	Chd9	Dnm3
Dlg2	Cpne2	Col26a1	Ccdc110	Chn2	Dysf
Dmd	Crip3	Cped1	Ccdc177	Cit	Ebf1
Dot1l	Crlf1	Crb2	Ccdc180	Clcn4	Eif2s3y
Dst	Csk	Creb5	Ccnd2	Clybl	Eif4e3
Dusp5	Ctif	Csf2	Ccr9	Cmklr1	Eps15l1
Dym	Ctnna3	Ctdspl	Cd276	Cmss1	Erg28
Dync1i1	Cux2	Ctif	Cd93	Cnnm3	Extl1
Dzip1	D13Ertd608e	Cuedc1	Cdc14a	Col18a1	Fam160b2
Eci2	Dact1	Cul1	Cdca7l	Col27a1	Fam171a2
Eif4g3	Dennd1a	Cul9	Cdh22	Col6a2	Fam19a4
Eml4	Dgkh	Cux2	Cfap53	Commd1	Fam20b
Enoph1	Dio1	Cygb	Cflar	Coro2b	Fat4
Epcam	Dlc1	Cyp26c1	Chp2	Cpsf4l	Fbrs
Esrrg	Dlgap2	Cyp46a1	Chtf8	Cradd	Fig4
Esyt3	Dll1	D430019H16Rik	Cic	Crim1	Fktn
Etv4	Dnah10	Dag1	Cit	Csf2	Fpr3
Fam171a2	Dnajc7	Dcaf4	Cks1b	Csgalnact1	Fto
Fam32a	Dnm3	Ddah1	Clcn4	Cxxc5	Fut4
Fam53b	Dysf	Ddx3y	Cldn14	Cycs	Fut7
Fancd2os	Ebf2	Dennd2a	Clstn2	Cyp46a1	Gata5
Fbxo21	Edem1	Des	Cmip	Dazl	Gcn1l1
Fbxo28	Egln1	Dhdds	Cnbd2	Ddn	Gm10800
Fer1l6	Elmo1	Dip2c	Col11a1	Ddx3y	Gm13090
Fes	Elp4	Disc1	Commd1	Dip2a	Gm20388
Fgfr2	Epb41l1	Dnah6	Coro1c	Dlc1	Gm20481
Fignl2	Epha4	Dnajc18	Cpne5	Dll3	Gm20503
Foxn3	Etv6	Dnase1l2	Crb2	Dnah6	Gm20517
Gm10775	Evl	Dnmbp	Crb3	Dnajc12	Gm20696
Gm16867	Evpl	Dot1l	Creb3l3	Dnm3	Gm37013
Gm20388	Fam160a1	Dpp9	Creb5	Dnmt3a	Gm37388
Gm28048	Fancd2os	Dusp6	Crebbp	Drc1	Gm42416
Gm3264	Farp1	Dync1h1	Cry2	Dtx2	Gm45140
Gm37013	Fbxo30	E4f1	Cuedc1	Dym	Gm45711

Gm37388	Fbxo46	Ebf1	Cux2	Ect2l	Gm5475
Gm38393	Fezf2	Edaradd	Cyp26a1	Egr4	Gnas
Gm42416	Fgf12	Efhd2	Cyp26c1	Epas1	Gng10
Gm42878	Fktn	Elfn1	D13Ertd608e	Epb41l1	Gng7
Gnas	Fosl1	Elk3	D430019H16Rik	Epcam	Gpc6
Gramd1b	Foxn3	Enc1	Dab1	Epha6	Gphn
Grik3	Foxo6	Endou	Dab2ip	Ephb1	Gpx8
Grip1	Frmd6	Eng	Dcun1d4	Ephb2	Grk1
Gse1	Fut4	Erf	Ddn	Errfi1	Gse1
Gstm5	Fzd5	Erich2	Dedd	Esyt1	Gsx2
Has3	Gas7	Esrrg	Dgkz	Etv6	H13
Hdac5	Gata2	Etv6	Dip2b	Evx1	Hcn2
Hivep3	Gfap	Fam126a	Disc1	Faim2	Hdac4
Hlx	Gja4	Fam83h	Dlgap1	Fam102a	Hdac5
Hrk	Gli1	Fbf1	Dlgap2	Fam167a	Hivep3
Hs3st3b1	Gli2	Fbxo24	Dlgap4	Fam171a2	Hk2
Igdcc3	Gm10295	Fbxw19	Dmpk	Fam32a	Hs3st3a1
Igf2r	Gm10775	Fbxw9	Dmrt1	Fcrl5	Hsd17b11
Insyn1	Gm20388	Fezf2	Dmrt3	Fer1l6	Hsp90ab1
Itpk1	Gm28048	Fibp	Dmrta1	Fgfr1	Hspa1a
Jcad	Gm37240	Foxo6	Dnah7b	Fgfr2	Hydin
Kalrn	Gm9945	Frmd4a	Dnaja3	Filip1l	Igf2r
Kcna5	Gnas	Frmd4b	Dnm3	Foxn4	Irx4
Kcnk12	Gpld1	Frmd6	Dock9	Frmd7	Itpka
Kif1a	Gpsm1	Fry	Dok5	Frrs1	Itpr3
Klf14	Grb10	Fto	Dst	Fryl	Itsn1
L3mbtl3	Grid2	Gcn1l1	Dtnb	Frzb	Jak3
Lgr5	Grik3	Ggt5	Dync1i1	Fstl4	Jph2
Lhx4	Grip1	Glt1d1	Dzip1	Gata5	Kat2a
Lin7a	Grm2	Gm128	E2f7	Gata6	Kcnh6
Lingo1	Gse1	Gm14295	Efcab6	Gfap	Kcnk4
Lmo4	Hcn2	Gm14399	Egr3	Ggn	Kctd3
Lrrc18	Hcrtr2	Gm20388	Eif4e	Glyctk	Kdm6b
Map3k10	Hes7	Gm20708	Elf2	Gm10717	Kif6
Map7	Hexim1	Gm37013	Elmsan1	Gm10718	Kmt5a
Mapkapk5	Hip1	Gm37388	Emx2	Gm10800	Krt35
Marveld2	Hivep2	Gm42416	Endou	Gm14295	Lekr1
Marveld3	Hmg20b	Gm5617	Enox1	Gm14399	Lrfn1
Meltf	Hs3st1	Gnas	Eps8l1	Gm20388	Lrrc43
Mettl14	Hs3st3a1	Gpd1	Ern2	Gm20503	Lzts2
Mex3a	Hs6st3	Grap2	Errfi1	Gm28048	Mark1
Mfhas1	Hydin	Grb10	Esrrb	Gm45234	Mcts2
Mfsd12	Igf2r	Grin1	Etl4	Gm8281	Mecom
Mn1	Impdh1	Grin3b	Etnk2	Gnas	Meis1
Mthfsl	Iqcg	Gse1	Etv5	Gng10	Mettl7a3
Mylk	Itpk1	Gtf2i	Ext2	Gpr37l1	Mn1

Mzf1	Jph2	Hk3	Fat1	Grik4	Mpp2
Nefm	Kbtbd11	Htr1d	Fbf1	Grin1	Mrm2
Nfix	Kcnip1	Iffo2	Fbxo21	Gstt2	mt-Atp6
Nhsl1	Kctd15	Impact	Fbxo46	Hcn1	mt-Atp8
Nkpd1	L3mbtl1	Inpp5f	Fezf2	Hhipl1	mt-Co1
Nnat	Lhx6	Ipo13	Fkbp10	Hip1	mt-Co2
Nr1d1	Lrp8	Iqsec3	Fmnl1	Hk3	mt-Cytb
Nrp2	Lrrtm3	Irx1	Foxp4	Hlcs	Mtmr7
Nrxn2	Ltbp4	Isyna1	Frmd5	Hmga2	mt-Nd1
Olig1	Macf1	Itsn1	Fxn	Homer2	mt-Nd2
Pcdhga1	Magi1	Jak3	Fzd5	Hspg2	mt-Nd6
Pcdhga10	Map7d1	Kazn	Gabbr2	Htr2a	Mtnr1a
Pcdhga11	Mapk15	Kcnd3	Gapvd1	Hunk	Mtss1
Pcdhga12	Mapt	Kcng1	Gfpt2	Igf2r	Myrip
Pcdhga2	Meis2	Kcnh6	Gfra1	Il3ra	Mzf1
Pcdhga3	Mmp9	Kcnip3	Ggnbp1	Inava	Nacc2
Pcdhga4	Mn1	Kcnj11	Ggta1	Inf2	Nap1l3
Pcdhga5	Mtcl1	Kcns1	Gjb2	Itga9	Napsa
Pcdhga6	Myh9	Kdm4a	Glp1r	Itpr3	Nectin4
Pcdhga7	Myo18a	Kif13a	Gltpd2	Jcad	Neurl3
Pcdhga8	Myo1d	Ksr2	Gm14295	Jph3	Nhej1
Pcdhga9	Myt1l	Lca5l	Gm17018	Kalrn	Nt5dc3
Pcdhgb1	Neurl3	Lefty1	Gm20388	Kcnip1	Ntrk2
Pcdhgb2	Nfix	Lhfp	Gm28048	Kcnj4	Nudt1
Pcdhgb4	Nkiras2	Lhx2	Gm37013	Kcnk2	Pak4
Pcdhgb5	Nmral1	Lpp	Gm37388	Kcnq4	Pcdha1
Pcdhgb6	Nova2	Lrba	Gm42416	Kirrel3	Pcdha11
Pcdhgb7	Npdc1	Lrg1	Gm49322	Klhl29	Pcdha12
Pcdhgb8	Nptx2	Lrr1	Gnas	Krt80	Pcdha2
Pcdhgc3	Nrtn	Lrrc29	Gng12	Krtap17-1	Pcdha3
Pcdhgc4	Nrxn1	Lrrc32	Grasp	Large2	Pcdha4
Pcdhgc5	Nrxn2	Lrrc69	Grb10	Lca5l	Pcdha5
Pgrmc1	Nup62cl	Lsm4	Grid1	Lgi2	Pcdha6
Phyhip	Nyx	Mamstr	Gsc	Lin28a	Pcdha7
Pigf	Obscn	Man1c1	Gsg1l	Lingo1	Pcdha8
Pik3c2b	Oprd1	Map9	Haao	Lmf1	Pcdha9
Plagl1	Ostm1	Marveld2	Hhipl1	Lpp	Pde4d
Pld6	Otop2	Mb	Hk1	Lrp5	Pfn3
Plekhg4	Pax5	Mcu	Hpca	Lrrc1	Phactr4
Pnmal2	Pax6	Mdga1	Iffo2	Ltbp3	Phc2
Pnpt1	Phactr1	Med13l	Ifrd2	Map2k7	Pitpnc1
Podn	Phldb1	Meis2	Igf2r	Map3k14	Plpp1
Pou2f1	Phospho1	Mex3a	Il17ra	Mapk1ip1l	Pnpt1
Pou3f1	Pih1h3b	Mfsd12	Ildr1	Mapkap1	Pop5
Prdm16	Pik3c2b	Mmp11	Impact	Mcc	Pptc7
Prkca	Pik3r1	Morn1	Irs2	Me3	Prdm8

Prkce	Pla2r1	Mpl	Irx5	Mecom	Prex1
Prr12	Plaur	Mpo	Itga9	Meis1	Prickle2
Ptprn2	Plin1	Mtg1	Itm2c	Meis2	Prune2
Ralgapa2	Plxna1	Mtss1l	Itpkb	Mesp2	Ptch1
Rbfox3	Pou6f2	Mylk	Itpr1	Mgat5	Ptpn11
Rmdn1	Ppp1r13l	Myo18a	Jakmip1	Mgat5b	Ptprf
Rora	Prmt8	Myo18b	Jarid2	Mgll	Ptprg
Rpl5	Prorsd1	Myo3b	Kazn	Mmp11	Rab43
Rusc2	Proser2	Nav2	Kbtbd11	Mn1	Rasal3
Sdk1	Ralgds	Nbn	Kcnc1	Morn1	Rilpl1
Sec14l5	Rasal3	Nfix	Kdm2b	Mospd1	Rin1
Sesn1	Rbmxl2	Nkpd1	Kif13a	Mpo	Rnf26
Sgcd	Rgs22	Nkx2-3	Kpna1	Mpp2	Rtbdn
Sh2d3c	Rnf121	Nod1	Krtap17-1	Mrap	Scn1b
Sh3pxd2a	Rtn1	Nol4l	Lamb2	mt-Atp6	Selenoi
Shroom3	Ryr1	Nos3	Lats2	mt-Atp8	Sema4f
Six1	Scrt1	Nr2f1	Lbh	mt-Co1	Sik2
Slc19a2	Sdk2	Nsf	Ldlrad4	mt-Co2	Six1
Slc22a3	Sft2d1	Numa1	Lef1	mt-Cytb	Slc10a7
Slc25a13	Sgcd	Nxph4	Lhx9	mt-Nd1	Slc11a2
Slc39a11	Sh3gl3	Obscn	Lmtk2	mt-Nd2	Slc13a2
Slit3	Sh3pxd2b	Obsl1	Lrfn2	mt-Nd4	Slc16a12
Smad6	Shank3	Ocstamp	Lrp5	mt-Nd5	Slc26a10
Smurf1	Shisa3	Olfr279	Lrp8	Mtss1	Slc2a9
Snrnp70	Shisal1	Onecut3	Lrrc4b	Mtx1	Slc7a3
Snrpn	Sis	Otud7a	Lrrc8e	Muc1	Socs5
Snurf	Slc12a8	Oxsr1	Ltbp2	Mxi1	Sorl1
Sox15	Slc36a1	Oxt	Ltbp4	Myf5	Speg
Sox21	Slc41a3	P4htm	Macrod1	Myo1h	Spock1
Srcin1	Slc8a1	Pacrg	Mafg	Nav2	Ssbp4
Srgap1	Smad7	Panx2	Magi1	Ncam1	St6galnac3
Stat1	Smarcc1	Pax6	Mapk10	Nckap5l	Taf7l
Sycp2l	Smyd4	Pcdhga1	Mdk	Ncor2	Tbx15
Syt6	Snx4	Pcdhga2	Me3	Nedd4l	Tcf24
Tbkbp1	Sorcs2	Pcdhga3	Mecom	Nfatc2	Tdrd7
Tbx2	Sowaha	Pcdhga4	Meioc	Nfic	Tecr
Tcf4	Sox6	Pcdhga5	Mex3a	Nfix	Tenm3
Terf1	Sp9	Pcdhga6	Mgmt	Nhej1	Tenm4
Tet3	Spag16	Pcdhga7	Mical3	Nhsl1	Tfr2
Tfr2	Spidr	Pcdhga8	Mier2	Nkx2-2	Tiam1
Tle3	Stx1a	Pcdhgb1	Mkrn3	Notch4	Tjp2
Tmem125	Synj2	Pcdhgb2	Mlxipl	Noto	Tlx1
Tmem150c	Syt6	Pcdhgb4	Mn1	Nr2f1	Tmem132b
Tmem242	Tbc1d1	Pcgf3	Morn1	Nrp2	Tmem150c
Tmem268	Tbk1	Pcmtd1	Mpo	Nrxn2	Tmem164
Tmem54	Tbx4	Pcsk6	Mrc2	Ntn4	Tmem165

Tmem59l
Tnfaip2
Tnrc18
Tnxb
Trove2
Tspan17
Ttc28
Ttll11
Tuba8
Uck2
Upf1
Vstm2b
Vwf
Wdfy4
Wipf1
Zbtb7c
Zfp275
Zfp385a
Zfp444
Zfp462
Zfp536
Zrsr1

Tbxa2r
Tenm2
Tfap2c
Tfr2
Thtpa
Tmem132c
Tmem163
Tnik
Tpcn1
Tpgs1
Traip
Trak1
Trim17
Trub1
Tsc22d1
Tshz2
Tspan15
Tspan18
Tspoap1
Ube2a
Uchl1
Ush1g
Vps37b
Vrk3
Vwa8
Wnt3
Wnt3a
Wwox
Zfhx2
Zfp316
Zfp444
Zfp473
Zfp503
Zfp579
Zfp652
Zfp777
Zfp853
Zfpm1
Zrsr1
Zswim9

Pde10a
Pde4dip
Pde7b
Pfkfb4
Phc2
Phospho1
Pik3cd
Plekhg3
Plpp3
Plpp7
Ppfia4
Ppp2r1b
Ppp3cc
Prcd
Prickle1
Prkar1b
Prkce
Prr12
Prr5l
Prss44
Psmc3ip
Pter
Ptprn2
Ptprr
Ptprt
Pyy
Rai1
Ramp1
Rap1gap2
Rasip1
Rbfox3
Rbm7
Rhobtb1
Rims1
Rlf
Rnf121
Rnf138rt1
Rnf144b
Rnf150
Rxfp2
Rxra
Scyl3
Setbp1
Sft2d1
Sh2d3c
Sh3rf1

Msantd3
Mtf1
Mtus2
Mxra7
Myo18a
Myo1e
Ncor2
Nfatc1
Nfib
Nfkbil1
Nin
Nkx2-9
Nlrp5
Noto
Nova2
Npdc1
Npepl1
Nphs1
Npr3
Nr2f1
Nr2f2
Nxn
Nxph1
Oacyl
Olfm2
Onecut2
Ooep
Osbpl1a
Otx2
Oxr1
P3h4
Pacrg
Pacsin3
Panx2
Pax6
Pcdh10
Pcdhga1
Pcdhga10
Pcdhga11
Pcdhga12
Pcdhga2
Pcdhga3
Pcdhga4
Pcdhga5
Pcdhga6
Pcdhga7

Nxn
Nxph1
Nxph4
Odf2
Onecut3
Otx2
Pacrg
Pakap
Palm2
Peg10
Peg12
Phf21b
Phldb1
Pigk
Pik3cd
Pik3r1
Plch2
Pld5
Plekhg3
Pllp
Plpp1
Plpp7
Ppard
Ppm1h
Ppp1r3g
Ppp2r2c
Ppp3cc
Prdm16
Prex1
Prkcb
Prox1
Prrt4
Prss36
Ptges
Ptprg
Ptprr
Pttg1ip
Ranbp3l
Rex1bd
Rhoq
Rtn4rl2
Rusc2
Rxrg
S100a1
S100a13
Sdccag8

Tmem241
Tmem28
Tmem88
Tnik
Tor4a
Trappc9
Trub1
Tshz2
Tspoap1
Ttll8
Tubb5
Ubn2
Ubxn11
Usp2
Usp49
Usp50
Vdr
Vgll4
Wnt7b
Wnt8a
Wscd1
Zfp536
Zmynd8
Zp3r

Shank3	Pcdhga8	Sema6c
Shisa3	Pcdhga9	Serinc5
Shroom1	Pcdhgb1	Sgce
Sik3	Pcdhgb2	Sh2b2
Skida1	Pcdhgb4	Sh3pxd2a
Slc12a5	Pcdhgb5	Shisal1
Slc13a4	Pcdhgb6	Sirt1
Slc22a14	Pcdhgb7	Six1
Slc23a2	Pcdhgb8	Slc12a4
Slc25a37	Pcdhgc3	Slc14a2
Slc37a1	Pcsk6	Slc16a14
Slc4a1	Pdzd7	Slc20a2
Slc7a14	Pebp4	Slc25a13
Slc9a1	Pepd	Slc37a1
Slit3	Phactr1	Slc43a3
Smad3	Pipox	Slc7a3
Smim13	Pitpnm3	Slco3a1
Smtn	Plekha6	Smg6
Sobp	Plekha7	Smoc1
Sorcs2	Plekhh3	Sox6
Sp9	Pole4	Speg
Spats2l	Pou6f1	Spock1
Spsb1	Ppargc1b	Spred3
Sptbn4	Ppfia4	Sptb
Srrm3	Ppp1r13l	Srrm4
Suds3	Prag1	Ssbp3
Sugp2	Prdm11	St3gal3
Sulf2	Prdm16	St6galnac3
Sult2b1	Prkd2	St6galnac5
Syce3	Prr29	Stk39
T2	Prrc1	Stox2
Tacc1	Prss50	Suds3
Tbc1d16	Ptges	Supt5
Tbkbp1	Ptp4a2	Synpo
Tcte2	Ptprr	Tbxas1
Tead1	Pxn	Tcf4
Tenm4	Rab31	Tcf7l2
Tgfb1	Rai1	Tex2
Thpo	Rap1gap2	Tgfb1
Ticam2	Rarg	Tgfbr3l
Tmcc3	Rarres1	Thegl
Tmed7	Rbfox3	Tiam1
Tmem125	Rbmxl2	Tln2
Tmem151b	Reln	Tmem145
Tmem208	Rftn1	Tmem200c
Tmem63a	Rfx4	Tmem267

Tnrc18	Rims2	Tmem30c
Tor2a	Rnls	Tnks1bp1
Trim8	Rps9	Tnrc6b
Trp53i11	Rtn2	Tpm1
Trub1	Runx2	Traf3
Tsc22d1	Rxra	Tram2
Ttc16	Ryr1	Trp73
Ttc28	S1pr5	Tshz2
Ttc41	Scg2	Ttc23
Tubgcp6	Scn8a	Ttc4
Ubr2	Scrt2	Ttll6
Uroc1	Sdsl	Ubac1
Usp35	Sec16b	Unc5b
Usp46	Selenom	Usp15
Vav2	Sema4d	Usp35
Vps35l	Sfrp4	Usp6nl
Vstm2l	Sgsm1	Wbp1l
Vwf	Sh2d3c	Wipf3
Wdr66	Sh3gl3	Wnk4
Wdr86	Sh3pxd2a	Wwc1
Wnt7a	Shank3	Wwox
Wwc1	Shc1	Xkr7
Xndc1	Shh	Ypel2
Xntrpc	Six5	Ythdc2
Ypel2	Skap1	Zadh2
Zbtb20	Slc12a4	Zfand3
Zfp316	Slc25a21	Zfp36
Zfp365	Slc25a23	Zfpm1
Zfp503	Slc30a7	Zmat4
Zfp652	Slc32a1	Zmiz1
Zfpm1	Slc35e4	Zmynd8
Zmat4	Slc39a11	Zrsr1
	Slc44a5	
	Slc6a20a	
	Smad1	
	Smad7	
	Snd1	
	Sntg2	
	Snx18	
	Sobp	
	Socs2	
	Socs5	
	Sorcs2	
	Spata13	
	Speg	
	Spsb4	

Srcin1
Srgap3
Srrm4
Ssbp3
Stum
Sugp2
Supt5
Syt12
Tanc1
Tbx1
Tbx3
Tcf4
Tcf7l2
Thbd
Tjp2
Tmcc3
Tmem108
Tmem181a
Tmem63a
Tnfrsf1a
Tnrc6b
Tns1
Togaram2
Tomm40
Tpcn1
Traf3
Treh
Trim2
Ttbk1
Ttc34
Ttc7
Ttn
Tubgcp6
Ulk4
Unc5a
Unc5cl
Usp15
Utf1
Utp4
Vstm2l
Wap
Wbp1l
Wdr66
Wdr86
Wipf3
Wwc1

Xdh
Xkr6
Ypel1
Ypel4
Zfand3
Zfhx3
Zfp423
Zfp532
Zic1
Zic4
Zrsr1

Table S3. List of all 9 hypermethylated (A) and 7 hypomethylated (B) genes identified to be overlapping across the three timepoints in AL CCH mice compared to the AL Sham mice with their respective functions postulated or evidenced in the literature. The gene names have been appended with Alzheimer's Disease (AD), Mild Cognitive Impairment (MCI), Parkinsonism, Neurological diseases, Frontotemporal Dementia (FTD) according to their associations to the respective conditions in the literature. A total of 9 hypermethylated genes and 7 hypomethylated genes were identified to be overlapping across all three timepoints in the AL CCH mice and a total of 3 hypermethylated and 18 hypomethylated genes were identified to be overlapping across all three timepoints in the IF CCH mice.

A

Gene name	Function(s)
**Abcc4** (~AD)	Regulator of intracellular cyclic nucleotide levels; mediator of cAMP-dependent signal transduction to the nucleus.
**Gnas** (~AD)	Provides instructions for making stimulatory alpha subunit (G-protein→ signal transmitters/switches)
**Speg** (~MCI)	Marker for differentiated vascular smooth muscle cells (regulates growth and differentiation.
**Dnm3** (~parkinsonism)	Encodes GTP-binding proteins that associate with microtubules and are involved in vesicular transport.
**Actg1** (~AD)	Provides instruction for making of gamma-actin (→force transduction & transmission in muscle cells)
**Mn1** (~Neurological diseases)	Transcriptional co-regulator of RAR/RXR-mediated gene transcription
**Gm20388** (~AD)	Function remains unclear; probable link to LDL receptor class A domain (chol. metab)
**Igf2r** (~AD)	Clears Igf2 from cell surface to dampen signalling, transport lysosomal acid hydrolase from Golgi → lysosome
**Mecom** (~FTD)	Transcriptional regulator & oncoprotein; potential involvement in apoptosis, devt, cell differentiation and proliferation

B

Gene name	Function(s)
**Gse1** (~AD, FTD)	Encodes proline-rich protein which may be a subunit of HDAC
**Gnas** (~AD)	Provides instructions for making stimulatory alpha subunit (G-protein→ signal transmitters/switches)
**Nfix** (~AD)	Transcription factor; binds to palindromic promoters
**Ctif** (~AD)	Simultaneous editing and translation step; recognises premature termination codons
**Gm20388** (~AD)	Function remains unclear; probable link to LDL receptor class A domain (chol. metab)
**Cux2** (~AD, FTD)	Dendrite branching, spine devt, synapse formation in neuronal layers of cerebral cortex
**Actn1** (~AD)	Multiple roles in different cell types; Non-muscle cells→ binding actin to membrane

Table S4. Full list of differentially methylated genes across the three different timepoints under IF CCH with the breakdown of list of genes hyper and hypo-methylated respectively.

IF BCAS7D	IF BCAS15D	IF BCAS30D	IF BCAS7D	IF BCAS15D	IF BCAS30D
Acta1	Agap3	Abca4	A630023A22Rik	Abat	Aatk
Actn1	AI854703	Abcc4	Abcc12	Abcc4	Abca16
Adamts6	Akap9	Abi3	Abcc4	Abhd8	Abcc4
Adcy2	Angel2	Adcy5	Abi3	Acbd6	Acap2
Agap3	Ankrd13b	Adnp2	Abl1	Actg1	Acsf2
Ahnak2	Ankrd44	Adra1d	Ablim3	Adam12	Actg1
Alk	Antxr2	Aff3	AC149090.1	Adamts20	Actn1
Ank	Aqp6	Afmid	Acbd4	Ahdc1	Adamts14
Anp32a	Asic1	Agap2	Acot1	Ak7	Adamts3
Arhgap23	B3gat2	Akap6	Acsf3	Akap6	Adgrl4
Arid1b	B3gnt2	Alk	Acss1	Alox5ap	Agap1
Bag3	Bahd1	Anapc4	Actg1	Amotl2	Agap2
Bahcc1	Basp1	Anks1b	Actn1	Ank	Akr1d1
Baiap2	Bcor	Apba2	Adam12	Ank2	Alox5ap
Bcar3	Bin3	Appl2	Adamts17	Anks1b	Ank2
Bcor	Bmp2k	Arhgap21	Adamts2	App	Apbb1
Bri3	Bop1	Arhgap39	Adamtsl2	Arhgap27	Arhgap31
C3	Bri3	Arhgef28	Adamtsl5	Arhgdia	Arhgap9
Casz1	Cacna1a	B3gnt3	Adcy5	Arhgef28	Arhgef18
Cbarp	Cadm1	B4galnt4	Adgra2	Arx	Arid1b
Cdc42bpb	Casc1	Bach2	Adgrl4	Asap1	Arid2
Cdkn1b	Cbll1	Barhl1	Adra1b	Atf3	Arpp21
Chil1	Ccdc9b	Bcl2	Afap1	Atp9a	B3gat2
Chn1	Ccny	Bcor	Aff3	B4galt2	BC067074
Clstn2	Cdkn1c	Begain	Agap2	Bahcc1	Bckdhb
Clybl	Cep95	Brd2	Agap3	BC017158	Bcl11b
Cntfr	Cit	Cacna1a	Agbl5	Bcl6	Bcor
Col26a1	Commd1	Cacna1b	Agt	Bcl9l	Best2
Col2a1	Crip3	Cacna1c	Ahdc1	Bdh1	Bop1
Ctcfl	Ctdspl	Cacng7	Akap1	Bop1	Brd1
Ctdspl	Cx3cl1	Camkk2	Alox5ap	Bptf	Bst2
Dennd3	Cyp26b1	Camta1	Amn	Btbd9	C2cd4c
Diexf	Cyp26c1	Card6	Amotl1	C530008M17Rik	Cabin1
Dock8	Dclk2	Cbx6	Ankrd6	C77080	Cacna2d2
Dpysl2	Ddx5	Ccdc120	Anks1b	Cacnb4	Cadm1
Dusp8	Dusp4	Ccdc177	Anxa8	Cacng8	Camta1
Efna2	Dysf	Cdh22	Arhgap15	Camta1	Caskin2
Elfn2	E030030I06Rik	Cdk16	Arhgap22	Cav2	Cbln1
Enc1	Ebf1	Cdk8	Arhgap23	Cbx6	Ccdc120
Epha4	Elfn2	Celf4	Arhgap27	Ccdc88b	Cdc20b
Ephb1	Epha4	Celf5	Arhgap28	Ccl1	Cdh22

Epn1	Extl3	Chd9	Arhgap31	Ccnd1	Celf4
Esyt3	Fam129b	Chrd	Arhgap39	Cdkn1c	Cenpu
Fam129a	Fam171a2	Cib4	Arhgap9	Cdx4	Chad
Fam169b	Fam172a	Clasrp	Arhgef10	Celsr1	Chd5
Fam171a2	Fam19a5	Clpsl2	Arhgef10l	Cep128	Chsy3
Fblim1	Farp1	Col6a6	Arhgef17	Cers1	Cmip
Fbxo31	Fbxo10	Commd1	Arhgef2	Cfap46	Col26a1
Fignl2	Fbxw7	Coro2b	Arhgef40	Cfap61	Col4a2
Fkrp	Fgfr2	Cped1	Arrdc5	Chn2	Col9a1
Frrs1	Fig4	Crb2	Arx	Chst14	Comp
Fry	Fktn	Crybg3	Asap1	Clcn4	Cpne7
Gcnt4	Foxj3	Csf2	Asic1	Clip2	Crocc
Gm20388	Foxo6	Cux2	Atf7ip2	Clybl	Csf2ra
Gm20503	Frmd4a	Dact3	Atg7	Cmtm7	Ctif
Gm38393	Gadl1	Dbp	Atxn1	Col11a1	Cux2
Gnal	Gjb1	Dcdc2c	B3gnt7	Commd1	Cyld
Gnas	Glt1d1	Deaf1	Baiap3	Cpsf4l	Cyp24a1
Gnaz	Gm20388	Dst	BC067074	Cradd	Dbn1
Gng10	Gm20503	Dusp4	Bcar3	Crb2	Dcaf8
Grk5	Gm28048	Dusp6	Bcl7a	Crlf2	Dcbld2
Gse1	Gm3264	Efhd2	Bcor	Csmd1	Dclk1
Heca	Gm37240	Epha4	Begain	Cuedc1	Dgkh
Heg1	Gm9945	Ephb1	Bglap2	Cux2	Dixdc1
Hlcs	Gng10	Ephb4	Bicc1	Cxxc5	Dlgap1
Hmg20b	Grb10	Eras	Bin2	Cyp46a1	Dlgap2
Hs3st3a1	Grin2b	Exoc3l2	Brd1	Cyth1	Dlx1
Hs6st2	Grip1	Ext2	Bri3	D13Ertd608e	Dmtn
Igf2	Grm2	Fam32a	Brs3	Dbn1	Dnase2a
Jakmip1	Grtp1	Fam83h	Bsn	Ddx3y	Dnm3
Kalrn	Hcls1	Fbrsl1	Btbd17	Dgki	Dock9
Kank1	Hcrtr2	Fbxl13	Btrc	Dlgap2	Dok7
Kat14	Heg1	Fezf2	C1qtnf5	Dnm3	Ebf1
Kcnh7	Hic1	Fhod1	C2cd4c	Dnmt3a	Ebf2
Kcnip1	Hip1	Fmod	Cacna1c	Dtnb	Ece1
Klf6	Hivep2	Foxl1	Cacna1i	Dync1li2	Egln1
Klf7	Hydin	Foxn4	Camk2g	Dysf	Elfn2
Lef1	Igf1r	Foxo6	Camk2n1	Ece1	Eps15l1
Lhx4	Il1r1	Foxp4	Camkk1	Ect2l	Evx1
Lin7a	Impdh1	Frmd6	Camta1	Efna1	Fads1
Lmtk3	Irx3	Frmd8	Capn8	Ehbp1	Fam171a2
Lrba	Jph2	Frmpd1	Card11	Enc1	Fam189b
Lrfn1	Junb	Gabbr2	Casz1	Endou	Fam19a1
Lrp1	Kalrn	Gipc1	Ccdc177	Erf	Fam20b
Man1c1	Kcns3	Gli1	Ccdc30	Errfi1	Fam222a
Map7d1	Khsrp	Gm14295	Ccdc60	Esrp1	Fam53b
Mapt	Kiss1r	Gm20388	Ccnd2	Esyt1	Fgfr2

Marveld3	Kit	Gm20708	Ccnd3	Etl4	Fkbp7
Mast1	Larp1	Gm28048	Ccsap	Etv6	Flt4
Mb	Lmntd1	Gm37013	Cd276	Evpl	Fntb
Mbnl2	Lnx2	Gm37388	Cd93	Exph5	Foxp4
Mcu	Ltbp4	Gm42416	Cdc20b	Ext2	Frmd7
Mn1	Manf	Gm8108	Cdc42bpg	Faim2	Fst
Mthfsl	Map7d1	Gnas	Cdca7l	Fam19a4	Fstl4
mt-Nd1	Mapk15	Gramd1b	Cdh2	Fam32a	Fto
mt-Nd2	Mcoln3	Grin1	Cdh22	Fcgr3	Fut4
Mtss1	Mcu	Gse1	Cdh23	Fgf16	Fut7
Myo19	Mgll	Hk3	Cdh4	Fgfr2	Fyn
Nbeal2	Mn1	Hpn	Cdk19	Fmnl1	Fzd9
Nfatc2	Mrc2	Iars2	Cdkn1c	Fos	Gata2
Nfib	Mtcl1	Ildr1	Cers1	Foxn3	Gatm
Nfix	Nadk2	Inpp5f	Cfdp1	Frem2	Gcn1l1
Nhsl1	Nav2	Iqsec3	Chd9	Fry	Git2
Nkain2	Ndrg4	Islr2	Chn1	Fryl	Gm10800
Nkx2-2	Nectin4	Itpka	Chp2	Fstl4	Gm20388
Nlrp4e	Nek5	Jph3	Chrd	Gabbr2	Gm20503
Nmral1	Nipbl	Jph4	Chst8	Galm	Gm20517
Notch3	Nmral1	Kbtbd11	Chtf8	Galnt2	Gm38393
Nrxn1	Nol4l	Kcnip3	Cldn14	Gas7	Gm9857
Nxph4	Nr5a2	Kcnj14	Cldn4	Gm10800	Gng10
Otoa	Nrap	Kif18b	Clec3b	Gm1110	Gp1bb
Oxt	Nrg2	Kif26a	Cmip	Gm13090	Gpc1
Pak4	Otog	Kif5c	Cmklr1	Gm14295	Gpc6
Pak6	Pax8	Kifc1	Cmss1	Gm14399	Gpr153
Panx1	Pde4d	Kirrel3	Col11a1	Gm20388	Gpx8
Parvb	Phldb1	Klhdc7b	Commd1	Gm20671	Grik3
Pcnx2	Pip5k1c	Lingo2	Coro1a	Gm21863	Grik4
Pde10a	Pitpnm2	Lipo2	Coro1c	Gm28048	Grm8
Pdgfra	Plekhh3	Lipo3	Coro7	Gm43738	Gse1
Pdyn	Plxnd1	Lrrc32	Cpne5	Gm8281	Gys1
Peak1	Pnmal2	Lrrc69	Cradd	Gp1bb	H2-Q6
Pebp4	Polr1d	Lzts3	Crb2	Gpd1	Hcn1
Peg3	Pou6f2	Man1c1	Creb3l2	Gprc5b	Hcn3
Pet117	Ppfia4	Map4k1	Crtc3	Grik4	Henmt1
Pih1d1	Ppp1r26	Map7	Cry2	Grin2b	Hic1
Plekhg4	Ppp2r5b	Mapk11	Csf1r	Grk5	Hivep3
Porcn	Ppp4r1	Matn4	Ctdspl	Gse1	Hk1
Ppp2r2c	Prrt1	Mdga1	Cuedc1	Gtsf1	Hmg20b
Ppp3cc	Ptx3	Mfap3l	Cux2	H2-Q6	Hmga2
Prr12	Pxn	Mical3	Cxxc5	Hk3	Hnf4g
Ptch1	Rbfox3	Mpl	Cygb	Hpca	Hs3st3a1
Ptpn1	Rbm15b	Msl3	Cyp26b1	Hs2st1	Hs3st3b1
Rab19	Rbm33	Mtss1l	Cyp26c1	Hs3st3b1	Hsd17b11

Rasal1	Rbpms	Myh3	Cyp4x1	Hunk	Hsp90ab1
Rassf1	Rgl3	Mylk	Cyth4	Hyal1	Hspa12a
Rnf135	Rnf217	Neurl1a	D13Ertd608e	Ica1	Hspb1
Rnf39	Rtl1	Nkx2-3	D16Ertd472e	Igf2r	Hspg2
Rpl5	Runx1	Nlgn2	Ddr1	Igsf21	Igf2r
Rsph14	Runx3	Nos3	Dennd4a	Il3ra	Immp2l
Scube2	Rusc2	Npas3	Des	Inava	Inpp5j
Sec14l1	Sarm1	Npcd	Dgkh	Irf2	Insrr
Selenoi	Scrt1	Nr2f1	Dgkz	Irs1	Itgb5
Sema4f	Scx	Nr4a2	Dhh	Irs2	Itpkb
Sema6c	Sdk1	Nrp2	Dip2b	Islr2	Jak3
Serinc2	Sdk2	Nrxn1	Disc1	Itpk1	Kcnc4
Sgk1	Sgcd	Obscn	Dlg2	Itpr3	Kcnh6
Sgpl1	Sh3gl3	Obsl1	Dlgap2	Jmy	Kit
Sgpp2	Sh3pxd2a	Otog	Dlgap3	Jph3	Klf16
Sh3rf2	Sh3pxd2b	Otop2	Dmpk	Klf15	Lhx6
Shh	Shank2	Oxsr1	Dmrt3	Klhl29	Lmx1b
Slc19a2	Shroom4	Parp12	Dmrta1	Klk13	Lnx2
Slc8a2	Slc16a3	Pcdhga1	Dnaaf1	Lama1	Lpcat3
Smurf2	Slc2a12	Pcdhga10	Dnaic1	Lamb1	Lrp8
Smyd4	Slc35a1	Pcdhga11	Dnaja3	Lca5l	Lrrc8d
Snrpn	Slc38a10	Pcdhga12	Dnajc11	Lmf1	Lzts2
Snurf	Slc39a10	Pcdhga2	Dnajc18	Loxl1	Mad1l1
Sobp	Slc39a11	Pcdhga3	Dnmt3a	Lyzl4	Mapk15
Sox21	Slc4a8	Pcdhga4	Dock3	Lzts3	Mcph1
Sox7	Smad1	Pcdhga5	Dpf3	Mad1l1	Me3
Ssbp3	Smad6	Pcdhga6	Drd2	Mael	Med26
Syk	Smpx	Pcdhga7	Dscam	Maml2	Megf6
Taok3	Sorbs3	Pcdhga8	Dst	Maml3	Mfsd12
Tbx4	Sowaha	Pcdhga9	Dtx2	Map3k4	Mgat5b
Tcf15	Spata31d1d	Pcdhgb1	Dyrk3	Map3k7cl	Mmp2
Tecpr1	Speg	Pcdhgb2	Dysf	Mark1	Mmp24
Tenm3	Sptbn1	Pcdhgb4	E130114P18Rik	Marveld1	Mn1
Tenm4	Srrt	Pcdhgb5	E2f3	Mast4	Mrps27
Thegl	Strap	Pcdhgb6	Ece1	Mcoln2	mt-Atp6
Tmem125	Syt12	Pcdhgb7	Ect2l	Megf6	mt-Atp8
Tmem132c	Szt2	Pcdhgb8	Egln1	Mex3a	mt-Co1
Tnfaip1	Tacc2	Pcdhgc3	Egln2	mt-Cytb	mt-Co2
Trak1	Tbxa2r	Pcdhgc4	Egr3	Mtmr7	Mtf1
U2af2	Tcp11	Pcdhgc5	Eif4e	mt-Nd5	mt-Nd1
Ube3b	Tjp2	Pcolce	Elf2	Myl4	mt-Nd2
Usp29	Tmem132c	Pde11a	Elfn2	Myo15	mt-Nd6
Vti1a	Tmem268	Pde4dip	Elk3	Myo1e	Myo10
Vwf	Tox2	Pdx1	Emx2	Naa80	Myrfl
Wscd2	Tshz2	Peak1	En1	Nacc2	Mzf1
Zfp384	Ube2o	Pebp4	Epm2aip1	Ncoa3	Nacc2

Zfp536	Uchl1	Phospho1	Epn3	Ndst1	Nap1l3
	Uhrf1bp1	Pik3cd	Erg28	Nfix	Nbeal2
	Usp29	Plch2	Errfi1	Nipal3	Nbl1
	Utf1	Pmepa1	Esco1	Nkain1	Ncaph
	Veph1	Ppp2r3a	Esrrb	Nkx2-2	Necab2
	Vkorc1l1	Ppp3cc	Etv6	Notch1	Nectin4
	Vstm2l	Prdm16	Ext2	Npcd	Nfix
	Wdr95	Prr23a2	Eya2	Nphs1	Nhsl1
	Wnt3	Prr36	Ezh1	Nr2f1	Notch3
	Wnt7b	Psap	Fam102b	Nr4a2	Npffr1
	Zbtb16	Ptchd3	Fam110a	Nrg3	Nradd
	Zfp423	Pter	Fam171a2	Nt5c2	Ntrk2
	Zfp503	Rab31	Fam184a	Ntn4	Nxph4
	Zfp560	Rap1gap	Fam213a	Nup188	Olfm2
	Zfp777	Rassf3	Fam220a	Nxph1	Onecut2
	Zfp853	Rbfox3	Fam32a	Otop2	Panx1
	Zfpm1	Rbm19	Fam49a	Otx2	Papln
	Zrsr1	Rbms3	Fam78b	Pabpc4	Pars2
		Rbpjl	Fam81a	Papolb	Pbx1
		Rgs3	Fam83h	Parp1	Pcdh1
		Rnf135	Fbxl17	Peak1	Pde11a
		Rnf150	Fbxo21	Peg10	Pde4d
		Rnf26	Fbxo31	Peli2	Pear1
		Runx2	Fezf2	Phactr1	Pebp4
		Serinc3	Fgf4	Pih1d1	Peli2
		Set	Fgfr2	Pik3r1	Pepd
		Shank2	Fhod1	Pisd	Phactr3
		Sipa1l1	Flt1	Plekhg3	Pip5k1c
		Slc22a14	Fndc7	Plekhh2	Pitpnc1
		Slc23a2	Foxf1	Plpp7	Plagl1
		Slc25a37	Foxn4	Plxna4	Plcb4
		Slc2a12	Foxo6	Pmepa1	Plekhh3
		Slc39a11	Foxp4	Pnmal2	Plekho1
		Slc44a5	Frmd4b	Pop5	Pmepa1
		Slc9a5	Frmd6	Ppm1h	Pnpt1
		Sod3	Frzb	Ppp2r3d	Pop5
		Sox13	Fto	Ppp3ca	Ppp3ca
		Sox18	Furin	Prag1	Ppt2
		Sp9	Galm	Prex1	Prag1
		Spaca1	Galnt10	Prkag2	Prkca
		Speg	Gata2	Prkcb	Prorsd1
		Spock1	Gata6	Prn	Prox1
		Spsb1	Gfap	Prnp	Prrt1
		Sptb	Gfpt2	Prox1	Ptchd4
		Srgap1	Gfra2	Prss44	Ptger4
		St3gal4	Ggt1	Prune2	Ptpn11

Stat1	Ggt5	Psrc1	R3hdm2
Stn1	Gjb1	Ptp4a2	Rab40c
Sulf2	Gk	Ptprn	Rad23b
Supt3	Glg1	Ptpro	Ralgps1
Susd4	Gli2	Ptpru	Rasa3
Tbc1d16	Gli3	Pttg1ip	Rex1bd
Tbkbp1	Gm11032	Rab19	Rftn1
Tbx4	Gm14295	Rabac1	Rgs22
Thpo	Gm20388	Radil	Rimklb
Tln2	Gm26938	Rai1	Rnf220
Tm9sf2	Gm27021	Rasa3	Rspo1
Tmem125	Gm28048	Rcan1	Rtbdn
Tmem151b	Gm28308	Rfx4	Ruvbl2
Tmem59l	Gm37013	Rimklb	Scimp
Tmem80	Gm37240	Rlbp1	Sdk1
Tnk1	Gm37388	Rnf144b	Selenoi
Togaram2	Gm38393	Rnf157	Sema4f
Trp53i11	Gm42416	Rnf39	Sh2b2
Tsc22d4	Gnas	Rora	Sh2b3
Ttc7	Gne	Rusc1	Shank1
Ube3b	Gpalpp1	Rusc2	Shank3
Ush1g	Gpd1	Ryr1	Shh
Usp2	Gpr27	Sash1	Shisal1
Usp35	Gpsm1	Scarf2	Sik1
Vstm2l	Gpt	Scrt2	Sipa1l3
Wipf1	Gpx8	Sdccag8	Six1
Wnt10a	Grb10	Sgce	Six3
Wnt7b	Grb7	Sh3pxd2a	Ski
Zfp316	Grin1	Six1	Slc13a3
Zfp341	Grin3b	Six3	Slc25a48
Zfp365	Gse1	Slain2	Slc38a10
Zfp46	H13	Slc10a7	Slc7a1
Zfp560	Haao	Slc11a2	Smarcal1
Zfp652	Hdac5	Slc22a3	Smoc1
Zfpm1	Heg1	Slc37a1	Smpd5
Zrsr1	Hhipl1	Slc38a4	Snrpn
Zscan20	Hic1	Slc4a4	Snurf
	Hmgcl	Slc7a3	Socs5
	Hmgcr	Slit3	Spatc1
	Hnf1a	Smurf1	Speg
	Hnrnpul1	Sox13	Spry1
	Hoxa3	Sox6	Ssbp3
	Hoxa5	Speg	St8sia1
	Hsf5	Spock1	Sycp2l
	Hunk	Spx	Synrg
	Iffo2	Src	Tac1

Igf2r	Srgap1	Tamm41
Il6ra	Srrm4	Tbc1d16
Ildr2	St6galnac3	Tcf4
Impact	Stpg1	Terb1
Inava	Supt5	Tfr2
Inf2	Synj2	Tirap
Inhba	Tacc1	Tlx1
Inpp5a	Tanc1	Tmcc1
Ipcef1	Tbx18	Tmem119
Ipo5	Thegl	Tmem150c
Irs2	Tinagl1	Tmem164
Irx1	Tmc6	Tmem240
Irx3	Tmc8	Tmem267
Itga9	Tmem132b	Tmem28
Itgb3	Tmem242	Tnfaip2
Itgb4	Tnfrsf1a	Tnik
Itgb5	Tnrc18	Tns2
Itpkb	Tns2	Trappc10
Jak3	Tob2	Trappc9
Jph3	Tpcn1	Tspan7
Jup	Traf3	Ttc17
Katna1	Tram2	Ttc33
Kcna10	Trim26	Ttc7
Kcng2	Tshz2	Ubn2
Kcnh2	Tspan14	Umodl1
Kcnn4	Ttc21b	Unc5a
Kcnq1	Ttll11	Usp49
Kcp	Ucp3	Wdr27
Kdm4b	Ush1g	Wdr6
Kdr	Vash2	Wnt8a
Kifc3	Vasp	Xkr7
Kirrel3	Vps18	Zbtb2
Kntc1	Wbp1l	Zdhhc18
Krtap17-1	Wnt5b	Zfp521
Lca5l	Zbtb16	Zfp608
Lhx9	Zbtb20	Zfp697
Lima1	Zdhhc18	Zfp703
Lmf1	Zfp316	Zfp710
Lmna	Zfp385c	Zfp777
Lmntd1	Zfp444	Zmiz1
Loxhd1	Zfp503	Znrf1
Lpar3	Zfp536	
Lpcat3	Zfp697	
Lrp6	Zfp703	
Lrp8	Zrsr1	
Lrrc32		

Lrrc4
Lrrc8d
Ltbp4
Luzp2
Mad1l1
Maml2
Maml3
Mamstr
Man2a1
Map3k13
Map3k4
Mapk10
Mapk14
Mapk15
Mbnl2
Mcts2
Mdga1
Mdk
Med18
Med31
Mef2d
Mfrp
Mgat5b
Mindy4
Mlh1
Mllt1
Mn1
Mrap
Msi2
Msrb1
Mtcl1
Mthfd1
Myl4
Myo1e
Mypn
Mzf1
Nacc2
Nav1
Nav2
Nbeal2
Ncor2
Ndufc1
Necab2
Neurl3
Nfil3
Nim1k

Nos2
Notch2
Noto
Npepl1
Nr4a2
Nradd
Nrg1
Nrxn2
Nrxn3
Ntng2
Nufip1
Numa1
Nup210l
Olfm2
Onecut2
Opn4
Oprd1
Oprm1
Ost4
Otud7a
Pacsin3
Palld
Panx2
Pard6g
Parva
Pawr
Pax2
Pax6
Pcbp3
Pcdh9
Pcdhga1
Pcdhga10
Pcdhga11
Pcdhga12
Pcdhga2
Pcdhga3
Pcdhga4
Pcdhga5
Pcdhga6
Pcdhga7
Pcdhga8
Pcdhga9
Pcdhgb1
Pcdhgb2
Pcdhgb4
Pcdhgb5

Pcdhgb6
Pcdhgb7
Pcdhgb8
Pcdhgc3
Pde4c
Pdgfa
Pdgfd
Pdk1
Pdzd2
Pf4
Phactr3
Phf21b
Phlda3
Phldb1
Phospho1
Piezo2
Pigk
Pipox
Pkig
Plch2
Plek2
Plekha2
Plekhg4
Plin1
Plp2
Plpp3
Plpp4
Plpp7
Pnpla1
Pnpt1
Pou2f2
Ppard
Ppfia4
Ppp1r37
Ppp2r1b
Ppp2r5c
Prag1
Prcd
Prdm15
Prkca
Prkd2
Prkn
Prrc2b
Prrt4
Prss16
Prss50

Pstpip2
Ptger1
Ptgir
Ptgis
Ptp4a2
Ptpn1
Ptprn2
Pttg1ip
Pxk
Pyroxd2
Qrfp
Qtrt2
R3hdm2
Rab11fip4
Rab12
Rab31
Rai14
Rara
Rarg
Rarres1
Rasa3
Rasip1
Rbbp7
Rbfox3
Rbm19
Rex1bd
Rgs3
Rgs9
Rhobtb1
Ripor3
Rnf122
Rnf125
Rnf220
Rnf26
Rnmt
Rph3al
Runx2
Rxra
Sall1
Sbk1
Scarf2
Scd2
Sdc4
Sdk1
Sec11c
Selenoi

Selenom
Selp
Sema5a
Sergef
Sez6
Sh2b2
Sh3gl3
Sh3pxd2a
Shank3
Shb
Shisal1
Shroom1
Sipa1l1
Sirt4
Six5
Skap1
Ski
Slc26a2
Slc35e4
Slc44a5
Slc4a1
Slc9a5
Slit3
Smad1
Smad3
Smg6
Smtn
Snd1
Snrpn
Sntg2
Snurf
Snx18
Snx33
Sorbs3
Sorcs2
Sox6
Spef1
Speg
Srcin1
Srgap1
Srrm3
St6galnac2
St6galnac5
Stac3
Steap3
Stk24

Sugp2
Supt5
Syngap1
Syngr3
Tbc1d30
Tbk1
Tbx3
Tbxa2r
Tcf4
Tcf7
Tcf7l1
Tcf7l2
Tet1
Tex2
Thbd
Thpo
Thrap3
Tiam1
Tiam2
Tle2
Tm7sf3
Tm9sf3
Tmem119
Tmem132c
Tmem150c
Tmem159
Tmem181a
Tmem184a
Tmem191c
Tmem201
Tmem267
Tmem51
Tnfrsf1a
Tnks
Tns2
Tox2
Tpst2
Trappc9
Tsc22d4
Tshz1
Tshz2
Tspoap1
Ttbk1
Ttc28
Ttc34
Ttc7

Ubash3b
Unc5b
Unc79
Usf2
Ush2a
Utp4
Vac14
Vasn
Vav2
Vps8
Vwa5b1
Vwa5b2
Wasf2
Wdr66
Wipf1
Wipf3
Wnt10a
Wnt7a
Wnt8a
Wscd1
Wwox
Xkr6
Xkr7
Ylpm1
Ypel4
Zadh2
Zbtb20
Zbtb7c
Zdhhc18
Zfp407
Zfp423
Zfp462
Zfp503
Zfp608
Zfp652
Zfp710
Zfp853
Zfpm1
Zfyve21
Zic1
Zic4
Zkscan17
Zmynd8
Zrsr1

Table S5. List of all 3 hypermethylated (A) and 18 hypomethylated (B) genes identified to be overlapping across the three timepoints in IF CCH mice compared to the AL CCH mice with their respective functions postulated or evidenced in the literature. The gene names have been appended with Alzheimer's Disease (AD), Mild Cognitive Impairment (MCI), Frontotemporal Dementia (FTD), Parkinson's Disease (PD), Amyotrophic Lateral Sclerosis (ALS) and Vascular Dementia (VaD) according to their associations to the respective conditions in the literature.

A

Gene name	Function(s)
**Gm20388** (~AD)	Function remains unclear; probable link to LDL receptor class A domain (chol. metab)
**Ephor4** (~AD)	Negative regulator of neurotransmission and hippocampal synaptic plasticity
**Bcor** (~AD)	Corepressor and BCL6 repressor when overexpressed

B

Gene name	Function(s)
**Speg** (~MCI)	Marker for differentiated vascular smooth muscle cells (regulates growth and differentiation.
**Abcc4** (~AD)	Regulator of intracellular cyclic nucleotide levels; mediator of cAMP-dependent signal transduction to the nucleus.
**Cux2** (~AD, FTD)	Dendrite branching, spine devt, synapse formation in neuronal layers of cerebral cortex
**Igf2r** (~AD)	Clears Igf2 from cell surface to dampen signalling, transport lysosomal acid hydrolase from Golgi → lysosome
**Gm20388** (~AD)	Function remains unclear; probable link to LDL receptor class A domain (chol. metab)
**Gse1**(~AD,FTD)	Encodes proline-rich protein which may be a subunit of HDAC
**Actg1** (~AD)	Provides instruction for making of gamma-actin (→force transduction & transmission in muscle cells)
**Mad1l1** (~AD)	Component of mitotic spindle-assembly checkpoint, cell cycle control.
**Zdhhc18** (~PD)	Palmitoyltransferase catalysing addition of palmitate onto protein substrates.
**Camta1** (~ALS)	Transcriptional activator, involved in human episodic memory.
**Ece1** (~AD)	Type II integral membrane peptidase, involved in proteolysis of endothelin -1,2,3 to biologically active peptides.
**Nacc2** (~AD)	Transcriptional repressor through its association to nucleosome remodelling acetylase complex
**Dlgap2** (~AD)	Role in molecular synaptic organisation and neuronal cell signalling.
**Alox5ap** (~AD)	Required for leukotriene biosynthesis 5-lipoxyenase.
**Prag1** (~AD)	Acts as a scaffold protein, critical activator of Notch signalling.
**Fgfr2** (~AD)	Regulates cell proliferation and differentiation during development and tissue repair.
**Tns2** (~VaD)	Regulate cell proliferation and migration, important for cell to contract collagen gels.
**Rasa3** (~AD)	Critical regulator of Rap1, controlling adhesion properties and vascular lumen integrity.

Table S6. Full list of differentially methylated genes across the three different timepoints under AL CCH and IF CCH conditions respectively in the promoter region only.

AL BCAS7D	AL BCAS15D	AL BCAS30D	IF BCAS7D	IF BCAS15D	IF BCAS30D
Actg1	Abi2	Acin1	AC149090.1	Aqp6	Abca4
Anks1	Acsf2	Actg1	Actg1	Arhgdia	Acsf2
Asap1	Actg1	Actg1	Actg1	Atf3	Actg1
Atp6v1g2	Adamts1	Actg1	Adgrl4	B3gat2	Actg1
B230104I21Rik	Aen	Actg1	Asap1	Bcl9l	Adgrl4
Bahd1	Aen	Adam11	B3gnt7	Bmp2k	Arhgef18
Bcor	Ank3	Adgrb2	Baiap3	Bptf	Arid1b
Blcap	Ap1m2	AI464131	Brd1	C530008M17Rik	Arid2
Capn9	Atn1	Akr1b8	Camk2n1	C77080	Arpp21
Ccdc81	Atp4a	Arhgap8	Camk2n1	Cav2	Bcor
Ccnd2	Atp6v0a1	Arid2	Capn8	Cbll1	Brd1
Ccr9	Atp6v0a2	Atp11b	Ccdc30	Cdkn1c	Brd2
Cep57l1	Axin2	Bag3	Ccdc60	Cep95	Bst2
Chp2	Axin2	Banp	Ccnd2	Cfap46	Cacna1b
Chtf8	B3gat2	Baz2b	Cdh23	Clcn4	Ccdc120
Cic	B3gnt8	Bcor	Chd9	Commd1	Chad
Cks1b	Banp	Bok	Chil1	Cpsf4l	Chrd
Commd1	Banp	Brd1	Chp2	Cpsf4l	Chsy3
Commd1	Bcan	Catsperz	Chrd	Crip3	Clpsl2
Cpne5	Bcl11b	Ccdc60	Chtf8	Dbn1	Crb2
Crb2	Boll	Cd55b	Cntfr	Dclk2	Crocc
Crb3	C330007P06Rik	Chd9	Commd1	Ddx3y	Cyld
Creb3l3	Ccdc30	Chrd	Coro1a	Ddx5	Dbn1
Creb5	Celf6	Chsy3	Cpne5	Dnmt3a	Dbp
Crocc	Chad	Crb2	Crb2	Dysf	Dcaf8
Cyp26a1	Chst13	Ctdspl	Crtc3	E030030I06Rik	Dcbld2
Ddx3y	Ckb	Cul9	Ctcfl	Ehbp1	Deaf1
Dedd	Clcn4	Ddah1	Cyp4x1	Exph5	Dgkh
Dgkz	Cldn15	Ddx3y	Ddr1	Fam19a5	Dnase2a
Diexf	Col6a2	Dennd2a	Dgkz	Fbxw7	Dok7
Dkk2	Cpsf4l	Des	Diexf	Fcgr3	Dst
Dmrt3	Cpsf4l	Dll1	Dmrt3	Fktn	Dusp4
Dnaja3	Crip3	Dnah6	Dnaic1	Fry	Fam20b
Dst	Csk	Dnase1l2	Dnajc11	Fstl4	Foxn4
Dst	Cycs	Fam126a	Dnmt3a	Gm13090	Frmd7
Eif4g3	Ddx3y	Fam160b2	Dst	Gm21863	Fstl4
Eml4	Dennd1a	Fam20b	Emx2	Gm9945	Gatm
Emx2	Dgkh	Fbf1	Epm2aip1	Gng10	Git2
Esyt3	Dll1	Fbrs	Epn1	Gp1bb	Gm38393
Etv4	Dll3	Fbxw9	Esyt3	Grm2	Gm8108
Fbf1	Dnah6	Fktn	Ezh1	Hyal1	Gm9857

Fbxo21	Dnajc12	Frmd4b	Fam169b	Il3ra	Gnas
Ggta1	Dnmt3a	Gata5	Fam213a	Junb	Gnas
Gm10775	Dysf	Gm45711	Fblim1	Kalrn	Gng10
Gm17018	Ebf2	Gnas	Fezf2	Klf15	Gp1bb
Gnas	Epb41l1	Gnas	Fgf4	Klk13	Gramd1b
Gnas	Evl	Gnas	Foxf1	Lamb1	Grm8
Grid1	Fam32a	Gng10	Furin	Lnx2	Henmt1
Gstm5	Fancd2os	Grin3b	Gata2	Mad1l1	Hk1
Hpca	Fbxo30	Gtf2i	Ggt1	Mael	Hmg20b
Ifrd2	Fktn	Hivep3	Glg1	Map7d1	Hnf4g
Igf2r	Frmd7	Hk2	Gli2	Mcoln2	Hs3st3b1
Il17ra	Fstl4	Hsp90ab1	Gm38393	Megf6	Hsp90ab1
Kcnc1	Fut4	Inpp5f	Gnal	mt-Cytb	Inpp5f
Kif1a	Gli2	Ipo13	Gnas	Myl4	Inpp5j
Kpna1	Gm10717	Kcns1	Gnas	Myo15	Kifc1
Lamb2	Gm10775	Kdm4a	Gne	Naa80	Lzts2
Lmo4	Gm9945	Kmt5a	Gng10	Ndrg4	Mad1l1
Lrrc8e	Gnas	Krt35	Gng10	Nectin4	Msl3
Mapkapk5	Gnas	Lsm4	Grb10	Nfix	mt-Atp6
Marveld2	Gnas	Lzts2	Grin3b	Nol4l	mt-Atp8
Mdk	Gng10	Mamstr	Heg1	Otx2	mt-Co2
Meioc	Gpld1	Mcts2	Hic1	Parp1	mt-Nd1
Mettl14	Grb10	Mmp11	Hmg20b	Pde4d	mt-Nd2
Mkrn3	Gse1	Mmp11	Hmgcl	Peg10	mt-Nd6
Mthfsl	Hip1	mt-Atp6	Hmgcr	Pitpnm2	Ncaph
Nefm	Hmg20b	mt-Atp8	Ipcef1	Plpp7	Nectin4
Nfix	Il3ra	mt-Co2	Jup	Pop5	Nkx2-3
Nfkbil1	Kctd15	Mtg1	Kat14	Ppp2r3d	Npffr1
Nkx2-9	Krt80	mt-Nd1	Kcp	Ppp2r3d	Nradd
Npdc1	L3mbtl1	mt-Nd2	Kntc1	Ppp2r5b	Obsl1
Nr1d1	Large2	mt-Nd6	Lrrc4	Ppp4r1	Papln
Oacyl	Map7d1	Mtnr1a	Mamstr	Prss44	Parp12
Olig1	Meis2	Myo18a	Map7d1	Pxn	Pars2
P3h4	Mmp11	Nectin4	Mcts2	Rbm15b	Pcolce
Pacsin3	Mmp9	Nfix	Mdk	Rbm33	Pde4d
Panx2	mt-Atp6	Nkx2-3	Mef2d	Rlbp1	Phospho1
Pax6	mt-Atp8	Nod1	Mlh1	Rnf217	Plagl1
Pcdh10	mt-Co2	Nudt1	Mthfsl	Rnf39	Plcb4
Plagl1	mt-Cytb	Numa1	mt-Nd1	Rusc1	Pnpt1
Plekha7	mt-Nd1	Obsl1	mt-Nd2	Rusc2	Pop5
Pnpt1	mt-Nd2	Panx2	Myl4	Rusc2	Ppp2r3a
Podn	mt-Nd5	Pax6	Nbeal2	Sgce	Ppt2
Pole4	Mtx1	Pfn3	Ndufc1	Sh3pxd2a	Prrt1
Prdm16	Muc1	Phospho1	Nfatc2	Slc16a3	Psap
Prkca	Nfatc2	Plpp7	Nfix	Slc38a10	Rab40c
Prkd2	Nfic	Pnpt1	Noto	Slc7a3	Rnf135

Prr29	Nkiras2	Pop5	Nradd	Smpx	Rtbdn
Ptges	Nkx2-2	Prcd	Nrxn1	Sox13	Ruvbl2
Rarg	Npdc1	Prr5l	Nufip1	Sox6	Selenoi
Rbmxl2	Nrxn1	Prss44	Numa1	Speg	Sema4f
Rmdn1	Nrxn2	Psmc3ip	Pacsin3	Speg	Set
Rpl5	Nrxn2	Ptch1	Panx1	Sptbn1	Sh2b3
Rps9	Otx2	Ptprg	Pax6	Spx	Shank3
Rusc2	Pax5	Ptprt	Pdzd2	Srrt	Slc38a10
Rxra	Pax6	Rab43	Pf4	Stpg1	Slc9a5
S1pr5	Peg10	Rasal3	Phlda3	Supt5	Smoc1
Scg2	Phldb1	Rbm7	Phldb1	Synj2	Smpd5
Sesn1	Pih1h3b	Rhobtb1	Phospho1	Tbx18	Snrpn
Shc1	Pik3c2b	Rnf144b	Plekha2	Thegl	Snurf
Six1	Pik3r1	Rtbdn	Plekhg4	Tmc6	Sox13
Six5	Plin1	Rxra	Plin1	Tmc8	Spaca1
Slc12a4	Plpp7	Scyl3	Plpp7	Tmem268	Speg
Slc25a23	Ppp1r3g	Selenoi	Pnpt1	Tnrc18	Spsb1
Slc30a7	Ptges	Shisa3	Prcd	Traf3	St3gal4
Smurf1	Ranbp3l	Shroom1	Prdm15	Trim26	Stat1
Speg	Rasal3	Slc11a2	Prkca	Ush1g	Sycp2l
Speg	Rusc2	Slc16a12	Prkd2	Zbtb16	Tac1
Stat1	S100a1	Slc7a3	Prkd2	Zfp560	Tamm41
Supt5	Sgce	Smad3	Prrc2b	Zrsr1	Tbx4
Sycp2l	Shank3	Speg	Prss16	Zrsr1	Thpo
Terf1	Shisa3	Spsb1	Ptch1		Tlx1
Tle3	Sirt1	Suds3	Ptpn1		Tmcc1
Tmem268	Six1	Sult2b1	Pyroxd2		Tmem164
Tmem54	Slc12a4	Syce3	Qtrt2		Tmem267
Tmem59l	Slc43a3	Taf7l	Rab11fip4		Tmem59l
Tnfaip2	Slc7a3	Tbx15	Rara		Tnfaip2
Tomm40	Slco3a1	Tdrd7	Rarg		Tnk1
Treh	Smoc1	Tgfb1	Rbbp7		Togaram2
Trim2	Sox6	Thpo	Rgs3		Trp53i11
Ttc34	Sox6	Tlx1	Ripor3		Tsc22d4
Ttc7	Sp9	Tmem132b	Rnf135		Ube3b
Tuba8	Speg	Tmem164	Rnf39		Ubn2
Vstm2b	Spred3	Tmem208	Rpl5		Usp2
Wdr86	Ssbp3	Tmem88	Rxra		Usp35
Zfp275	Stx1a	Tor2a	Selp		Usp49
Zfp532	Suds3	Trim8	Serinc2		Zfp560
Zic4	Supt5	Tspoap1	Sgk1		Zfp608
Zrsr1	Tbx4	Ubn2	Shank3		Zfp710
Zrsr1	Tfap2c	Usp2	Shank3		Zrsr1
	Tgfbr3l	Usp35	Shroom1		Zscan20
	Thegl	Usp49	Sirt4		
	Tmem145	Vdr	Six5		

Tmem267	Vgll4	Slc9a5
Tmem267	Vps35l	Smurf2
Tpm1	Wdr86	Smyd4
Traf3	Xndc1	Snrpn
Traip	Xntrpc	Snurf
Tspoap1	Zfp536	Speg
Usp35		Speg
Vrk3		St6galnac2
Zrsr1		Stac3
Zrsr1		Supt5
		Syngap1
		Tcf15
		Tcf7
		Tecpr1
		Thbd
		Thbd
		Thegl
		Thpo
		Tle2
		Tm9sf3
		Tmem267
		Tspoap1
		Ttc34
		Usp29
		Zic4
		Zmynd8
		Zrsr1
		Zrsr1
		Zrsr1
